# Comprehensive analysis of optimal power flow using recent metaheuristic algorithms

**DOI:** 10.1038/s41598-024-58565-y

**Published:** 2024-06-11

**Authors:** Ahmed A. Zaki Diab, Ashraf M. Abdelhamid, Hamdy M. Sultan

**Affiliations:** 1 Department of Electrical Engineering, Faculty of Engineering, Minia University, Minia, 61111 Egypt; 2 Electronics and Communications Engineering Department, College of Engineering, Umm Al-Qura University, Al-Lith Branch, Saudi Arabia

**Keywords:** Metaheuristics, Optimal power flow, Fuel cost, Voltage profile, Voltage stability, Energy, Electrical and electronic engineering, Energy grids and networks

## Abstract

This paper provides six metaheuristic algorithms, namely Fast Cuckoo Search (FCS), Salp Swarm Algorithm (SSA), Dynamic control Cuckoo search (DCCS), Gradient-Based Optimizer (GBO), Northern Goshawk Optimization (NGO), Opposition Flow Direction Algorithm (OFDA) to efficiently solve the optimal power flow (OPF) issue. Under standard and conservative operating settings, the OPF problem is modeled utilizing a range of objectives, constraints, and formulations. Five case studies have been conducted using IEEE 30-bus and IEEE 118-bus standard test systems to evaluate the effectiveness and robustness of the proposed algorithms. A performance evaluation procedure is suggested to compare the optimization techniques' strength and resilience. A fresh comparison methodology is created to compare the proposed methodologies with other well-known methodologies. Compared to previously reported optimization algorithms in the literature, the obtained results show the potential of GBO to solve various OPF problems efficiently.

## Introduction

For power system operators, OPF is a crucial tool because it enables them to more effectively balance the supply and demand of electricity, lower the price of electricity production, and boost system dependability^1^. As it can assist in determining the ideal combination of generation resources and transmission facilities required to satisfy future demand growth, it is also used for long-term planning and design of energy systems^2^.

OPF is a method used in power systems engineering to allocate and use power generation resources as efficiently as possible to meet the demand for electricity while lowering costs and preserving system reliability. It entails resolving a mathematical optimization problem that accounts for a number of restrictions, such as the capacity of the transmission lines, the demand for electricity, and the generating limits^3,4^.

OPF's objective is to reduce energy production costs while meeting a variety of operational limitations, including voltage limits, generator capacity limits, and transmission line capacity limits. Mathematical programming approaches like linear programming, quadratic programming, or nonlinear programming can be used to address optimization problems, which often have many variables and restrictions^5,6^.

Numerous optimization approaches have been used to address the OPF issue over the course of the last few decades, and this has been the subject of extensive research. Numerous traditional deterministic optimization methods have had success in the past. According to the literature, most of these traditional methods use one of a number of techniques, such as gradient techniques, Newton's methodology, the Simplex methodology, sequential linear programming (SLP), sequential quadratic programming (SQP), and interior point methods (IPMs)^7^. Reference^7^ provides a summary of the typical optimization techniques that are most frequently employed to address the OPF issue. Although some of these deterministic methods have shown excellent convergence behaviour and are frequently employed in industry, they are not without drawbacks. One of their disadvantages is their inability to guarantee global optimality, which means they might converge to local optima. These methods were often designed under particular theoretical assumptions, such as convexity, differentiability, and continuity, which may not be appropriate for real OPF situations^7,8^. They are also not well-suited to handle binary or integer variables. Additionally, over the past 20 years, significant research in the field of heuristic optimization techniques has been done to address the OPF issue as a result of the fast development of recent computational intelligence tools^9^. To address the OPF issue, some of these techniques have been employed, including: Particle swarm optimization (PSO)^10^, Biogeography Based Optimization (BBO)^11,12^, artificial bee colony (ABC)^13,14^, Shuffle Frog Leaping Algorithm (SLFA)^15^, gravitational search algorithm (GSA)^16,17^, Grey wolf optimizer (GWO)^18^, Slime mould algorithm (SMA)^19^, Teaching Learning based Optimization (TLBO)^20^, modified pigeon-inspired algorithm (MPIO)^21^, backtracking search algorithm (BSA)^22^, Harmony Search (HS)^23^, Black Hole (BH)^24^, Harris Hawks Optimization (HHO)^25^, quasi-oppositional modified Jaya (QOMJaya)^26,27^, League Championship Algorithm (LCA)^28^, hybrid bat algorithm (HBA)^29^, and Group Search Optimization (GSO)^30^. These techniques are renowned for their capacity for quick searching of large solution spaces, their ability to avoid being constrained to local solutions, and their capability to take into account uncertainty in specific power system components. A survey of several optimization methods utilized to tackle the OPF issue is presented in^9,31^. Due to the diversity of objectives that can be taken into account when describing an OPF issue, no single method can be said to be the best when addressing all OPF problems. A new method that can effectively tackle some of the OPF difficulties is thus always needed.

In this paper, Fast Cuckoo Search (FCS)^32^, Salp Swarm Algorithm (SSA)^33^, Dynamic control Cuckoo search (DCCS)^34^, Gradient-Based Optimizer (GBO)^35^, Northern Goshawk Optimization (NGO)^36^, and Opposition Flow Direction Algorithm (OFDA)^37^ are utilized for tackling the OPF issue in the standard IEEE 30 Bus test system. A metaheuristic algorithm called Fast Cuckoo Search (FCS) was partly developed due to cuckoo bird behaviour. It uses heuristics and randomization to find the best answers^32^. Dynamic Control Cuckoo Search (DCCS) extends the standard cuckoo search method, which dynamically modifies its parameters while optimizing. With this strategy, DCCS can more quickly and effectively adapt to the shifting optimization environment^34^. Another metaheuristic algorithm that mimics the behaviour of salp swarms in the water is the Salp Swarm Algorithm (SSA). In order to obtain the best answers, SSA relies on the idea of social cooperation among the individuals in the swarm^33^. The Gradient-Based Optimizer (GBO) is a deterministic optimization method that seeks out the best answers by using gradient data^35^. The optimization method known as Northern Goshawk Optimization (NGO) was influenced by the way northern goshawks hunt^36^. To get the best answers, it combines local and global search techniques. These techniques have been developed to overcome some of the limitations of traditional optimization methods, such as their inability to find global solutions and handle uncertainties in the power system.

The following is a summary of this paper's significant contributions:

- Use of FCS, SSA, DCCS, GBO, NGO, and OFDA optimization methods for practical OPF situations.
- Putting into practice a comprehensive suite of tests to evaluate optimization algorithms employing various OPF issues, objective functions, and restrictions.
- Addressing the OPF issue while taking into account security restrictions for more difficult circumstances.
- The use of a novel comparison technique based on ideal and typical values.
- Incorporating non-parametric statistics for validating the proposed optimization technique.

The rest of the article is structured as follows. The OPF issue is stated in “Problem formulation” section. The suggested optimization methodologies are described in “Optimization algorithms” section. “Results and discussion” section presents application examples and outcomes. Finally, “Conclusion” section draws the findings.

## Problem formulation

OPF, as previously indicated, is a power flow issue that determines the ideal control variable adjustment for a specific load by minimizing a predetermined objective function, such as the generation price or transmission losses. Optimal power flow is a non-linear constrained optimization issue that takes the system's operational restrictions into account. It can be expressed as follows^38,39^:

1 $$\mathrm{Minimize \;\;F}({\text{x}},{\text{u}})$$

2 $$\mathrm{Subject\;\; to\;\; g}({\text{x}},{\text{u}}) = 0$$

3 $$\mathrm{and \;\; h}({\text{x}},{\text{u}})\le 0$$

Equation (1), in which ${\text{x}}$ signifies the vector of state variables, and ${\text{u}}$ signifies the vector of control variables, determines the objective function. The equality and inequality requirements are denoted by ${\text{g}}$ and $h$, respectively. The dependent variables are stated in Eq. (4), and the independent variables are shown in Eq. (5)^40,41^.

4 $${u}^{T} =\left[{P}_{{G}_{2}}.......{P}_{{G}_{NG}}, {V}_{{G}_{1}}........ {V}_{{G}_{NG}}, {Q}_{{C}_{1}}.........{Q}_{{C}_{NC}}, {T}_{1}........{T}_{NT}\right]$$

where ${{\text{P}}}_{{\text{G}}}$ represents the real power provided by the power plant, ${{\text{V}}}_{{\text{G}}}$ represents the level of the voltage at the generator buses, ${{\text{Q}}}_{{\text{C}}}$ represents the reactive power provided by the shunt compensators, and ${\text{T}}$ represents the position of the transformer's tapping adjuster. ${\text{NG}}$, ${\text{NC}}$, and ${\text{NT}}$ stand for the quantity of transformers, shunt capacitors, and generators, respectively^40,41^.

5 $${x}^{T} =\left[{P}_{{G}_{1}}, {V}_{{L}_{1}}........ {V}_{{L}_{NL}}, {Q}_{{G}_{1}}.........{Q}_{{G}_{NG}}, {S}_{{l}_{1}}........{S}_{{l}_{nl}}\right]$$

In the equation, the symbol ${{\text{P}}}_{{\text{G}}1}$ depicts the power at the slack bus, ${{\text{V}}}_{{\text{L}}}$ denotes the voltage values at the load bus, ${{\text{Q}}}_{{\text{G}}}$ represents the reactive power supplied by the generator, and ${{\text{S}}}_{{\text{l}}}$ symbolizes the flow of apparent power through the transmission line. The number of loads, generators, and transmission lines is denoted by the symbols ${\text{NL}}$, ${\text{NG}}$, and $nl$, respectively.

### System constraints

#### Equality constraint

These restrictions establish particular requirements that must be strictly met. They reflect relationships that must hold precisely and are frequently represented by equations. The power flow equations, which make sure that real and reactive power injections at each node in the power system are balanced, could be represented in an ORPD problem by equality constraints. For a workable solution, these equations would need to be completely satisfied^40,41^.

6 $${P}_{Gi} - {P}_{Di}-\left|{V}_{i}\right|\sum \limits_{j=1}^{{\text{NB}}}\left|{{\text{V}}}_{{\text{j}}}\right|\left[{G}_{{ij}}{\text{cos}}{\alpha }_{{ij}}+{B}_{{ij}}{\text{sin}}{\alpha }_{{ij}}\right]=0$$

7 $${Q}_{Gi}- {Q}_{Di} - \left|{V}_{i}\right|\sum\limits_{j=1}^{{\text{NB}}}\left|{{\text{V}}}_{{\text{j}}}\right|\left[{G}_{ij}{\text{sin}}{\alpha }_{ij}-{B}_{ij}{\text{cos}}{\alpha }_{ij}\right]=0$$

where ${P}_{Gi}$ and ${Q}_{Gi}$ denote the injection of real and reactive power at bus i. ${P}_{Di}$ and ${Q}_{Di}$ denote the actual and reactive power that the load at bus i draws. ${{\text{B}}}_{{\text{ij}}}$ is the branch's susceptibility between i and j buses and ${{\text{N}}}_{{\text{B}}}$ represents the total number of nodes.

#### Inequality constraints

The variables or parameters of the optimization problem are restricted or limited by inequality constraints. They express requirements that must be met within a set of constraints and are frequently represented by inequalities. In an ORPD situation, inequality constraints could be used to set restrictions on the thermal capacity of transmission lines, voltage magnitude limitations, and generator reactive power limitations. These restrictions limit the range of conceivable solutions and guarantee that the solution stays within allowable operational bounds.

##### Constraints for generator

All generators in the system should operate within the predetermined maximum and minimum tolerances for real power generation, reactive power generation, and bus voltage magnitude. The following can be used to indicate these variables' upper and lower boundaries^40,42^:

8 $${{\text{P}}}_{Gi}^{min}\le {P}_{Gi}\le {{\text{P}}}_{Gi}^{max} \quad \mathrm{For} \;\; i = 1,\dots \dots \dots ,NG$$

9 $${Q}_{Gi}^{min}\le {Q}_{Gi}\le {Q}_{Gi}^{max} \quad \mathrm{For} \;\;i = 1,\dots \dots \dots , NG$$

10 $${V}_{Gi}^{min}\le {V}_{Gi}\le {V}_{Gi}^{max} \quad \mathrm{For} \;\; i = 1, \dots \dots \dots , NG$$

where ${P}_{Gi}$ signifies the actual power produced from generator i, and ${{\text{P}}}_{Gi}^{min}$ and ${{\text{P}}}_{Gi}^{max}$ denote the actual power output's lower and upper bounds. ${Q}_{Gi}$ denotes the generator i's capacity to produce reactive power, and ${Q}_{Gi}^{min}$ and ${Q}_{Gi}^{max}$ denote the capacity's minimum and maximum levels, respectively. ${V}_{Gi}$ is the voltage magnitude at bus i, and ${V}_{Gi}^{min}$ and ${V}_{Gi}^{max}$ denote the voltage magnitude's lower and upper bounds, respectively.

##### Constraints for transformers

Transformers employ tap changers to modify the transformer turns ratio and, consequently, the voltage level. The number of tap positions and magnitude of voltage change that a tap changer can alter are both limited. These restrictions can be shown as^40,42^:

11 $${T}_{i}^{min}\le {T}_{i}\le {T}_{i}^{max}\quad \mathrm{For} \;\; i = 1, \dots \dots \dots , NT$$

where ${T}_{i}$ stands for the tap position of transformer i, and ${T}_{i}^{min}$ and ${T}_{i}^{max}$ stand for the tap position's lower and higher bounds, respectively.

##### Constraints for shunt capacitors

Reactive power compensation capabilities of some devices, such as capacitors or compensators, are constrained. The inequality restriction can be stated as follows, for instance, where ${Q}_{Ci}^{min}$ and ${Q}_{Ci}^{max}$ stand for the lower and upper bounds of reactive power compensation for a compensator i^40,42^:

12 $${Q}_{Ci}^{min}\le {Q}_{Ci}\le {Q}_{Ci}^{max} \quad \mathrm{For} \;\; i = 1, \dots \dots \dots ,NC$$

where ${Q}_{Ci}$ is the reactive power injected from compensator i.

##### Security constraints

To ensure the safe and reliable operation of the associated loads, load buses may have restrictions on their voltage levels. These restrictions can be expressed as^40,42^:

13 $${V}_{Li}^{min}\le {V}_{Li}\le {V}_{Li}^{max} \quad \mathrm{For} \;\; i = 1,\dots \dots \dots ,NL$$

where ${V}_{Li}$ signifies the voltage magnitude at load bus i, and ${V}_{Li}^{min}$ and ${V}_{Li}^{max}$ denote the lower and upper boundaries, respectively, for the load voltage level. To prevent overheating and prospective damage, transmission cables have thermal capacity restrictions. The inequality restriction, for instance, can be written as follows if ${S}_{TLi}^{max}$ represents the thermal limit of transmission line i^40,42^:

14 $${S}_{li}\le {S}_{li}^{max} \quad \mathrm{For } \;\; i = 1,\dots \dots \dots , nl$$

where ${S}_{TLi}$ is the complex power flow on the transmission line i.

### Objective function

It is important to note that control variables are bound by themselves. An objective function can incorporate quadratic penalty factor to account for the inequality constraints of dependent variables that include line loading, active power produced at slack bus, reactive power generated, and load bus voltage magnitude. In this case, the objective function is multiplied by a penalty term that equals the square of the disregard value of the dependent variable, and any impractical solution found is rejected. The following is a mathematical way to express the penalty function:

15 $$\mathrm{Penalty }= {\upgamma }_{{\text{P}}}{\left({P}_{{G}_{i}}-{P}_{{G}_{i}}^{lim}\right)}^{2}+{\upgamma }_{{\text{V}}}\sum_{{\text{i}}=1}^{{\text{NL}}}{\left({{\text{V}}}_{{{\text{L}}}_{{\text{i}}}}-{{\text{V}}}_{{{\text{L}}}_{{\text{i}}}}^{{\text{lim}}}\right)}^{2}+{\upgamma }_{{\text{Q}}}\sum_{{\text{i}}=1}^{{\text{NG}}}{\left({{\text{Q}}}_{{{\text{G}}}_{{\text{i}}}}-{{\text{Q}}}_{{{\text{G}}}_{{\text{i}}}}^{{\text{lim}}}\right)}^{2}+{\upgamma }_{{\text{S}}}\sum_{{\text{i}}=0}^{{\text{nl}}}{\left({S}_{{l}_{i}}-{S}_{{l}_{i}}^{lim}\right)}^{2}$$

where ${\upgamma }_{{\text{P}}}$, ${\upgamma }_{{\text{V}}}$, ${\upgamma }_{{\text{Q}}}$ and ${\upgamma }_{{\text{S}}}$ denote penalty terms and ${x}^{lim}$ signifies the limiting of the dependent variable $x$. When $x$ is greater than the maximum bound, ${x}^{lim}$ will be equal to the value of this one, and when $x$ is lower than the minimum bound ${x}^{lim}$ will be equal to this limit:

16 $${x}^{lim}=\left\{\begin{array}{ll}{x}^{max};& \quad x>{x}^{max}\\ {x}^{min};&\quad x<{x}^{min}\end{array}\right.$$

#### Case 1: Minimization of generation fuel cost

The goal of Optimal Power Flow (OPF) problems is to reduce the cost of generating while taking into account the limitations of the system. Both fixed expenses and variable costs make up the generation cost in most cases. The power generation equipment's fixed costs are related to the initial capital expenditure, whereas the variable costs are related to the equipment's use and upkeep. The variable costs are the most critical consideration when minimizing the generation cost in an OPF situation. The variable costs are typically described as a quadratic function of the power output and depend on how much electricity is produced by each generator. The OPF problem's objective function is expressed as follows:

17 $${f}_{i}={(a}_{i}+{b}_{i}{P}_{{G}_{i}}+{c}_{i}{P}_{{G}_{i}}^{2}) (\$/h)$$

where, for the ith generator, ${a}_{i}$, ${b}_{i}$ and ${c}_{i}$ stand for, respectively, the standard cost rate, the linear cost rate, and the quadratic cost rate. Consequently, the objective function below can be used to express the system's overall fuel cost for all generators.

18 $$F(x,u)=\sum_{i=1}^{NG}{f}_{i}(\$/h)+Penalty$$

#### Case 2: Voltage profile improvement

One of the most crucial and critical indicators of safety and service goodness is voltage magnitude at buses. Therefore, reducing the cost of fuel used for the entire generation process might provide a workable solution, but the voltage profile might not be suitable. Calculating the total voltage deviations of PQ buses is one method of evaluating the goodness of the voltage shape^43^. The following is how the sum of the voltage variations reported is expressed:

19 $$\mathrm{VD }=\sum_{{\text{i}}=1}^{{\text{NL}}}\left|{{\text{V}}}_{{{\text{L}}}_{{\text{i}}}}-1\right|$$

20 $$F(x,u) = \sum_{{\text{i}}=1}^{{\text{NL}}}\left|{{\text{V}}}_{{{\text{L}}}_{{\text{i}}}}-1\right|+{\text{Penalty}}$$

#### Case 3: Voltage profile enhancement with minimization of fuel cost

Here, lowering costs while simultaneously enhancing the voltage profile is the goal. As a result, we have a dual objective function in this case, as provided by:

21 $$F(x,u) =\left(\sum_{i=1}^{NG}{(a}_{i}+{b}_{i}{P}_{{G}_{i}}+{c}_{i}{P}_{{G}_{i}}^{2})\right)+\left({\upkappa }_{{\text{VD}}}\sum_{{\text{i}}=1}^{{\text{NL}}}\left|{{\text{V}}}_{{{\text{L}}}_{{\text{i}}}}-1\right|\right)+{\text{Penalty}}$$

where ${\upkappa }_{{\text{VD}}}$ denotes a weighting factor that needs to be properly determined. Each of the two sections of the objective function is assigned a weight (an importance) by this selection. After numerous experiments with trial and error, the study's chosen value for ${\upkappa }_{{\text{VD}}}$ is 500.

#### Case 4: Voltage stability improvement

Voltage stability becomes a necessity in reality since power systems are under a lot of stress. A voltage stability index (${{\text{L}}}_{{\text{index}}}$) has been created by Kessel and Glavitch^44^ regarding the viability of power flow equations for each bus. It ranges from 0 to 1, with 0 and 1 representing circumstances of no load and voltage breakdown. Alternatively, the ${{\text{L}}}_{{\text{index}}}$ value measured at a bus determines whether voltage collapse is likely to occur at that bus. Therefore, to improve the voltage stability of the grid, it is important to minimize the maximum ${{\text{L}}}_{{\text{index}}}$ or ${{\text{L}}}_{{\text{max}}}$.

22 $$F(x,u) ={{\text{L}}}_{{\text{max}}}+{\text{Penalty}}$$

#### Case 5: Voltage stability improvement with minimization of fuel cost

Therefore, the following dual objective function to simultaneously improve voltage stability represented by ${{\text{L}}}_{{\text{max}}}$ and minimize the total cost of generating fuel has been suggested as follows:

23 $$F(x,u) =\left(\sum_{i=1}^{NG}{a}_{i}+{b}_{i}{P}_{{G}_{i}}+{c}_{i}{P}_{{G}_{i}}^{2}\right)+{\upkappa }_{{{\text{L}}}_{{\text{max}}}}\times {{\text{L}}}_{{\text{max}}}+{\text{Penalty}}$$

where ${\upkappa }_{{{\text{L}}}_{{\text{max}}}}$ is a scaling factor used to balance out the values of the objective function and prevent one objective from taking precedence over another. The value of ${\upkappa }_{{{\text{L}}}_{{\text{max}}}}$ in this study is set at 5000.

#### Case 6: Minimization of active power losses

This target tries to reduce all real power losses in the power system by maximizing reactive power dispatch. Real power losses can be decreased to increase system performance and lower the cost of energy supply. The expression of the objective function for minimizing the transmission losses is given as:

24 $$F(x,u) ={{\text{P}}}_{{\text{loss}}}+{\text{Penalty}}$$

#### Case 7: Minimization of Reactive transmission power losses

A secondary goal of an OPF issue is to reduce reactive power losses in the lines of the system in addition to lowering the cost of generation. Reactive power is the energy transferred from the generator to the loads in order to keep the system's voltage stable. Reactive components, such as capacitors and inductors, contribute to reactive power losses in the lines. Maximizing the generators' production of reactive power and strategically placing reactive compensating devices such as shunt capacitors and reactors can reduce these losses to a minimum. An OPF problem's objective function that includes minimizing reactive power losses is written as follows:

25 $$F(x,u) ={{\text{Q}}}_{{\text{loss}}}+{\text{Penalty}}$$

## Optimization algorithms

The implementation of the optimization algorithms for solving the OPF problem can be outlined as follows:

1. Input Data Collection: The input data for system components such as lines, branches, generators, loads, and constraints are computed.
2. Optimization Technique Parameters: Parameters such as the number of individuals, iterations, and population size are determined.
3. Initialization of Individuals: Individuals from the solution set are randomly distributed within the solution space according to each algorithm's methodology.
4. Main Optimization Loop: The optimization method is executed using the initial objective function value, and constraints are checked against predefined limits.
5. Recording Best Solutions: The best value for each individual in the solution set is recorded and designated as the current better solution, comparing it with neighboring solutions.
6. Position Update: Based on each algorithm's updated position strategies, new solutions are generated. If a new solution outperforms the previous one, position adjustments are made. Otherwise, the current position remains the optimal solution.
7. Final Solution: The process continues until termination requirements are met, such as reaching a predefined objective function value or maximum iteration limit, typical in OPF problems.

### Fast Cuckoo Search (FCS)

Cuckoo Search (CS) algorithm is a contemporary metaheuristic algorithm inspired by nature and gained extensive utilization in addressing challenging optimization problems. CS draws its inspiration from the brood parasitism behavior observed in cuckoo birds^45^. It employs a well-balanced combination of a local random walk and global random walks, which are governed by a switching coefficient called ${p}_{a}$. The local walk is expressed as follow:

26 $${x}_{i}^{t+1}={x}_{i}^{t}+\alpha s\otimes H\left({p}_{a}-\in \right)\otimes \left({x}_{j}^{t}-{x}_{k}^{t}\right)$$

${x}_{j}^{t}$ and ${x}_{k}^{t}$ represent two distinct candidate solutions that are chosen randomly through a process of random permutation. ${\text{H}}({\text{u}})$ refers to a Heaviside relation, while $\in$ represents an arbitrary number obtained according to uniform distribution. The variable ${\text{s}}$ represents the step size. In this context, the symbol "$\otimes$" denotes the entry-wise operation between two vectors. The global walk is executed using a specialized form of random walk known as Lévy Flights. The initial population is selected within predefined parameter bounds, which define the range of values within the domain.

#### Cuckoo breeding behavior

Certain species of cuckoos, such as Ani and Guria cuckoos, engage in a behavior known as brood parasitism, where they lay their eggs in communal nests. They may even destroy the host birds' eggs to increase the chances of their eggs hatching. By doing so, they enlist the host birds to raise their offspring, allowing the cuckoos to allocate more time and energy to laying additional eggs instead of parental care. The hosts can be individuals of the same species or different species. If a cuckoo chooses a nest of another individual of the same species to lay its eggs, it is referred to as “intra-specific brood parasitism”^45^.

Host birds have developed strategies to detect foreign eggs. If they identify an egg as not their own, they may either destroy it or abandon the nest to create a new one in another place. Some cuckoo species have evolved the ability to mimic the color and pattern of certain selected ones. They also select the timing and location of egg-laying intelligently. They lay their eggs in old nests, in which the host birds have recently laid eggs that will take longer to hatch. This leads to increased competition for food, causing the host bird chicks to frequently starve in the presence of cuckoo chicks. This behavior reduces competition, ensuring the survival of cuckoo chicks by decreasing the number of competing host chicks in the nest. In many cases, a cuckoo chick eliminates the eggs or kills the newly hatched host chicks. It repeats such behavior until it remains the sole occupant of the nest.

#### Cuckoo search via Lévy flights

In the case of a minimization problem, the fitness value can be inversely proportional to the value of the objective function. Alternatively, fitness functions can be formulated in a manner similar to genetic algorithms, where the principle of the "fittest chromosome (solution) survives" is applied. In the CS, each solution is likened to an egg in a nest, and a new solution represents an egg. The goal is to replace inadequate solutions with fresh ones that could be better. An updated solution, denoted as ${\text{x}}(\mathrm{t }+ 1)$, is produced by choosing a cuckoo, i, through the utilization of Lévy flights. Equation (27) illustrates the production of the updated solution.

27 $${x}_{i}^{t+1}={x}_{i}^{t}+\alpha \otimes Levy\left(\lambda \right)$$

Here, $\mathrm{\alpha }$ represents a positive value known as the step size, which varies depending on the specific problem at hand. In many cases, $\mathrm{\alpha }= 1$ is commonly employed. The Lévy flight, on the other hand, entails a random walk where the length of each random step is determined by a Lévy distribution. The Lévy distribution can be described by: Lévy $\sim \mathrm{ u }=\mathrm{ t}-\uplambda$(where $1 <\uplambda \le 3$). This distribution possesses an infinite variance and mean values.

#### Fast cuckoo search algorithm

The standard CS algorithm relies solely on random walks for search, which does not guarantee fast convergence^32^. Moreover, the replacement of old nests with lower-quality solutions is performed randomly, further diminishing the algorithm's convergence speed. In the proposed algorithm, a different approach is introduced where the global best solution directs the substitution of outdated nests. This method improves control over the step size while also quickening convergence. The updated equation for replacing old nests is formulated as:

28 $${nest}_{new}={nest}_{old}+rand\left({best}_{nest}-{nest}_{old}\right)\otimes K, \quad if \;\; K>{p}_{a}$$

**Figure 1:**
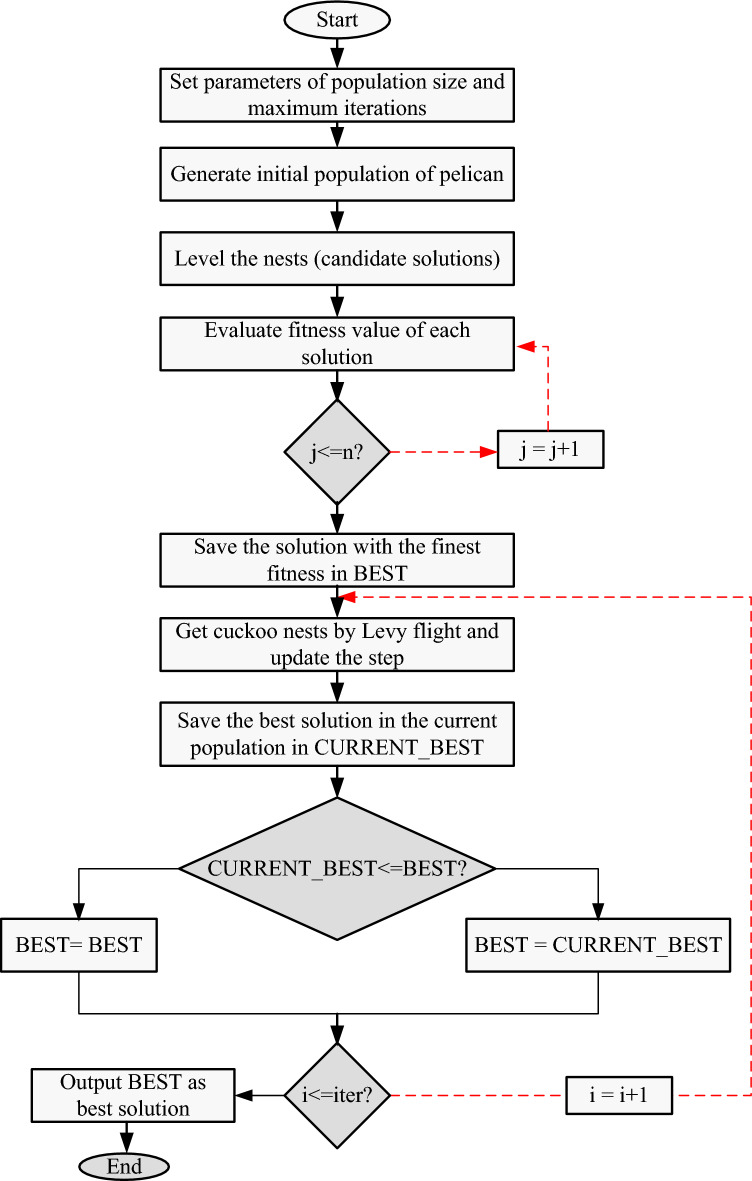
Flowchart of FCS optimization algorithm.

The equation involves the variables ${nest}_{old}$ and ${best}_{nest}$, which represent permutation matrices produced from the old nest and the global best one. The variable ${nest}_{new}$ denotes the new nest created in the present iteration^32^. The proposed methodology aims to enhance the convergence characteristics and is therefore called the Fast Cuckoo Search (FCS) algorithm. In the case of FCS, the best nest up until the last iteration is employed. This characteristic of FCS algorithm maintains selection pressure regarding superior solutions, ensuring better outcomes. Furthermore, this improvement in the CSA avoids population overcrowding with highly fit solutions. Flowchart of the FCS algorithm is presented in Fig. 1.

### Dynamic control Cuckoo Search (DCCS)

Inspired by the breeding manner of cuckoos, the original CS technique is a global optimization methodology that mimics the natural process of cuckoos searching for nests and laying eggs by incorporating the Levy flight mechanism observed in birds. Professor Yang and Deb suggested three optimum algorithmic states^34^:

Each generation, a cuckoo lays a single egg and chooses a nest at random for incubation. Until a better nest is found, future reproduction takes place in the best nest at the moment. The number of available nests remains constant for every generation, and there is a probability, ${\text{Pa}}$, that the egg is discovered by the host. In the event that the egg is found, the host may choose to either leave the egg or the complete nest and look for a good place to establish a new nesting. Here, ${\text{Pa}}$ denotes the probability of the host bird recognizing the egg as not its own offspring. ${\text{Pa}}$ is commonly set to 0.25 in research.

The conventional CS approach simulates the searching mechanism of cuckoo for nests by generating a candidate population, choosing optimal choices, and performing random migrations. Building upon the aforementioned ideal states, the algorithm determines the search route and position of the cuckoos with reference to (29) and after that, generates candidate populations.

29 $${x}_{i}^{t+1}={x}_{i}^{t}+\alpha \otimes L\left(\beta \right)$$

Among these variables: ${x}_{i}^{t+1}$ and ${x}_{i}^{t}$ implies to the position vectors of the ith bird nest position in the (t + 1)th and tth generations, respectively. ${x}_{i}^{t}$ represents the vector components of the bird nest's position, with ${\text{d}}$ representing the dimensionality of each nest. The index ${\text{j}}$ signifies any specific dimension, ranging from 1 to d. The variable t denotes the iteration number of the algorithm, starting from $\mathrm{t }= 1$ and ending at ${\text{tc}}$ when the algorithm converges. The value of ${\text{tc}}$ varies depending on the convergence of the algorithm. The parameter $\alpha$ is a constant factor known as the step-size factor, which controls the range of random search. Its value is positive and can vary depending on the specific situation. In the equations, the symbol $\otimes$ represents point-to-point multiplication, while $L\left(\beta \right)$ represents the random optimization route following the Levy flight mechanism. The term $\alpha \otimes L\left(\beta \right)$ denotes the step size of the Levy flight, representing the distance that a cuckoo needs to discover from the ith to the (i + 1)th generation in a randomly distributed way based on the Levy flight. The relationship between the Levy flight's random optimization path and the iteration time, ${\text{t}},$ follows a Levy distribution as given in (30). $\beta$ represents the exponential parameter, and $\mu$ denotes arbitrary number taken from a normal distribution. The expression illustrates how the CS algorithm's optimization route alternates between frequent small jumps and infrequent lengthy jumps. This approach enables the algorithm to explore a larger search area and facilitates escaping from local optima.

30 $$Levy\left(\beta \right)\sim \mu ={t}^{\beta },+\left(1<\beta \le 3\right)$$

To execute the CS algorithm, and to simulate the flight jump route that determines the step size. The step-size calculation method is demonstrated as follow.

31 $$Levy\left(\beta \right)=\frac{\mu }{{\left|v\right|}^{\frac{1}{\beta }}}$$

Among these variables: $\mu$ and $v$ are random numbers drawn from a normal distribution, $\beta$ is a parameter representing skewness, typically set to $\beta$ = 1.5.

In the context of an actual optimization issue, the position of the nest, ${x}_{i}^{t}$, denotes the feasible solution area for all variables in the problem (with d dimensions). The fitness values associated with each nest correspond to the objective function value for different variable values. During the evolution approach, after modifying the bird nest's position using (29), a random number, denoted as r and ranging from 0 to 1, is compared with the probability ${p}_{a}$. If r is greater than Pa, the position ${x}_{i}^{t+1}$ is randomly changed; otherwise, it remains unchanged. A set of bird nest positions with improved fitness is ultimately retained and represented as ${x}_{i}^{t+1}$.

By analyzing the original CS method and existing research findings, it is evident that the step-size factor, denoted as "$\alpha$" reduced linearly with advance in the simulation process. This linear decrease enhances the algorithm's convergence speed and improves its local exploration capabilities. Moreover, the step-size factor goes down as the fitness values vary, leading to improved convergence accuracy and solution quality. However, in the original CS methodology, the step-size factor continuously and randomly variations without considering the algorithm's progress, which results in slow convergence in the final stages. Additionally, the parameter skewness, represented by "$\beta$" in Eq. (31), significantly influences the production of the step-size.

To address these limitations, this study introduces the concept of the iteration ratio and fitness ratio to dynamically adapt the parameter skewness "$\beta$" and, consequently, the step-size. This dynamic adaptation is achieved by incorporating Eq. (32), which includes a dynamic balance factor denoted as "$\mathrm{\vartheta }$." The balance factor ensures a proper weighting of the iteration ratio and fitness ratio within the algorithm, balancing the trade-off between convergence speed and accuracy.

32 $${\beta }_{t}={\beta }_{min}+\vartheta \left(1-\frac{t}{m}\right)\left(1-\vartheta \right)\frac{{f}_{min}}{{f}_{max}}, \beta \in \left[1, 2\right]$$

**Figure 2:**
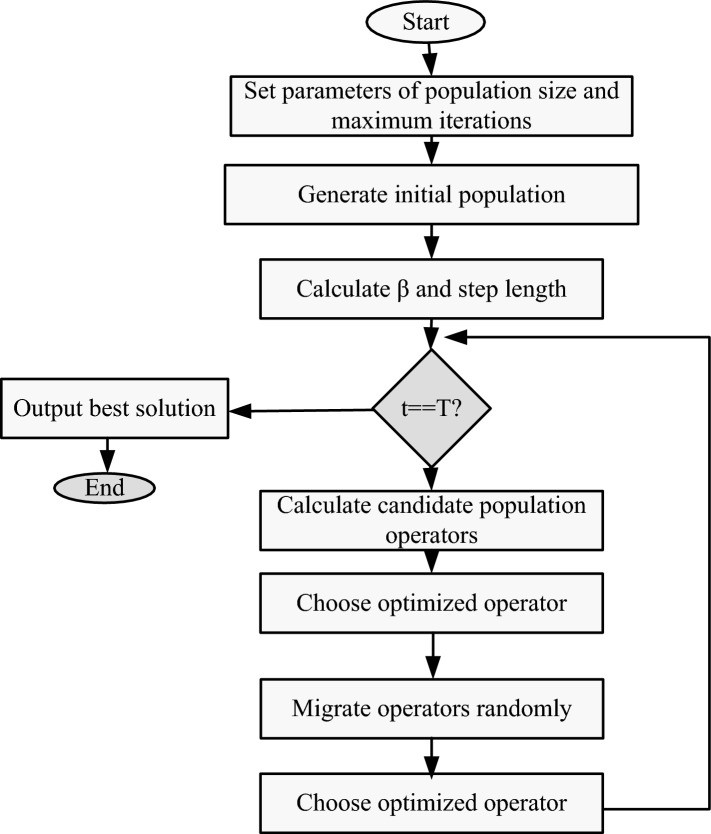
Flowchart of DCCS algorithm.

In the proposed method, several parameters are introduced to enhance the step-size adaptation in the CS method. The minimum parameter skewness value, denoted as "${\beta }_{min}$" serves as a lower bound for the skewness parameter. A dynamic balance coefficient, represented by "$\vartheta$" is applied manage the proportion of the iteration ratio and fitness ratio in the step-size calculation. This factor is constrained to the range (0,1). With advances in the optimization program, the step-size decreases continuously in the algorithm. However, to prevent premature convergence and improve convergence accuracy, the introduction of the iteration ratio ($\frac{t}{m}$) and the fitness ratio ($\frac{{f}_{min}}{{f}_{max}}$) plays a crucial role. The iteration ratio reduces the step-size during the algorithm's progression, thereby accelerating convergence. On the other hand, the fitness ratio ensures that the step-size decreases as the program approaches the optimal solution. By dynamically adjusting these two ratios through the balance factor "$\vartheta$" the algorithm avoids blind and rapid convergence with increasing iterations and mitigates the risk of premature convergence and falling into local optima. Consequently, this approach improves both the convergence characteristics and solution quality of the CS technique. The flowchart of the DCCS algorithm is presented in Fig. 2.

### Salp Swarm Algorithm

**Figure 3:**
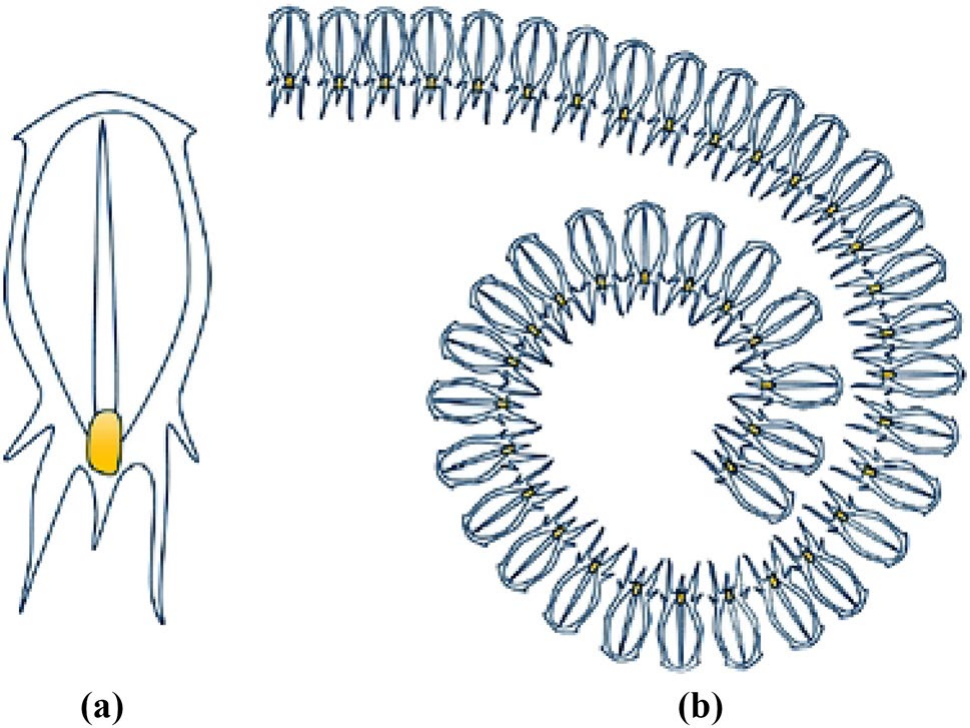
(**a**) single salp and (**b**) swarm of salps.

The Salp Swarm Algorithm (SSA) is an optimization technique that draws inspiration from the movement of salps, small marine animals that expand and contract their bodies to move (see Fig. 3). First proposed in 2017 by Mirjalili et al., the SSA is a meta-heuristic algorithm that aims to solve complex engineering problems^33^. The SSA involves a population of simulated salps, where each salp represents a potential solution to the optimization problem. These virtual salps move in the search space by following a set of equations that imitate the movement patterns of real salps. During each iteration of the algorithm, the fitness of each salp is assessed, and the most promising solutions are selected for the next generation. The SSA utilizes several parameters to control the behavior of the salps, including the salp step size and the attraction and repulsion coefficient. In the modeling process, salps are divided into two groups: the leader and the followers. The leader, located at the front of the swarm, guides the group in their quest for food and prey, while the remaining salps are considered followers who trail behind the leader. The salp chain model aims to efficiently explore and exploit the space around both stationary and mobile food sources, including identifying the positions of local and global optimal solutions^33^.

Suppose there is a specific system that requires optimization, where ${\text{N}}$ represents the number of variables to be optimized, ${\text{X}}$ corresponds to the position of a particular salp, and ${\text{M}}$ represents the objective, or source of food, that the salp swarm aims to achieve. The leader's position in the search process is updated using the following equation^33^:

33 $${x}_{i}^{1} =\left\{\begin{array}{ll}{y}_{i}+{r}_{1}\left(\left({ub}_{i}-{lb}_{i}\right){r}_{2}+{lb}_{i}\right)& \quad {r}_{3}\ge 0\\ {y}_{i}-{r}_{1}\left(\left({ub}_{i}-{lb}_{i}\right){r}_{2}+{lb}_{i}\right)& \quad {r}_{3}<0\end{array}\right.$$

In the above equation, ${x}_{i}^{1}$ and ${y}_{i}$ represent the position of the first salp and the position of the food source, respectively, in the ith dimension. The values of $lb$ and $ub$ correspond to the lower and upper bounds of the ith dimension. The variables ${r}_{1}$, ${r}_{2}$, and ${r}_{3}$ denote randomly generated numbers.

Of the three random numbers mentioned, ${r}_{1}$ is the most important, as it helps to maintain a balance between exploration and exploitation during the search process. The expression for ${r}_{1}$ is as follows:

34 $${r}_{1}= 2{e}^{-{\left(\frac{4l}{L}\right)}^{2}}$$

The equation for ${r}_{1}$ incorporates the maximum number of iterations, denoted by $L$, the current iteration represented by $l$, and two randomly generated numbers in the range of [0,1]. Newton's law of motion is used to update the positions of the followers, and the equation for this update is as follows:

35 $${x}_{i}^{j} =\frac{1}{2}\lambda {t}^{2}+{\delta }_{0}t$$

where $j \ge 2$ and $x_{i}^{j}$ signifies the location of the *jth* salp in the *ith* dimension, *t* is the time, ${\delta }_{0}$ is an initial speed, and $\uplambda =\frac{{\delta }_{final}}{{\delta }_{0}}$ , where $\updelta =\frac{{\text{x}}-{{\text{x}}}_{0}}{{\text{t}}}$.

**Figure 4:**
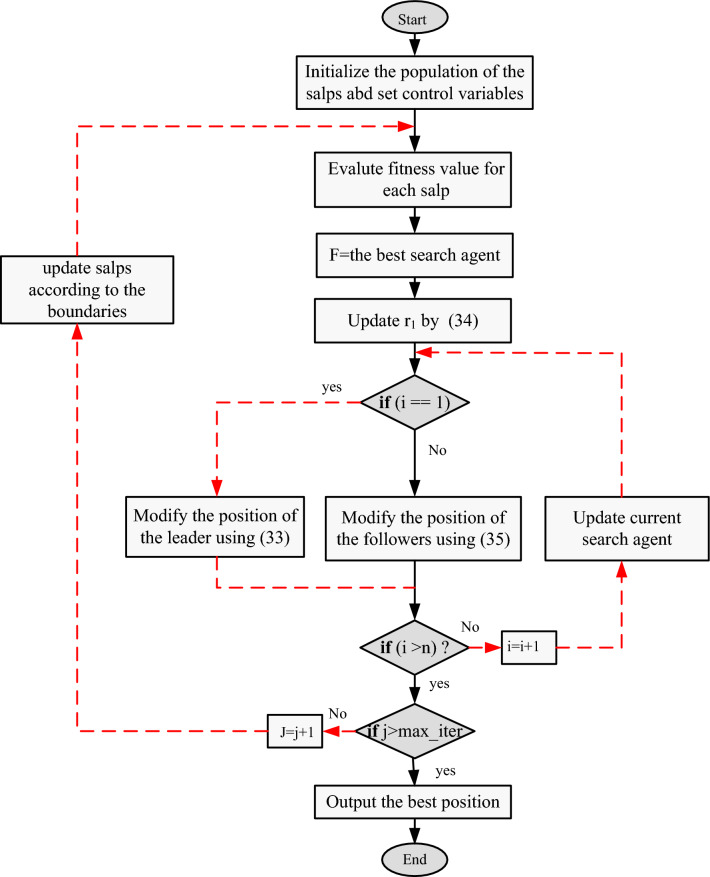
Flowchart of SSA.

In optimization models, the time interval t is equivalent to the iteration, and the initial speed ${\delta }_{0}$ is set to 0. With this in mind, the equation for updating the positions of the followers can be expressed as follows:

36 $${x}_{i}^{j} =\frac{1}{2}\left({x}_{i}^{j}+{x}_{i}^{j-1}\right)$$

where ${\text{j}}\ge 2$. The aforementioned equation indicates that the followers update their position based on their own position and the position of the salp preceding them. To ensure that the salps remain within the defined search area, a constraint equation is used to bring any salps that go beyond the predefined boundaries back into the search space. Figure 4 illustrates a flowchart of the Salp Swarm Algorithm (SSA).

37 $${x}_{i}^{j} =\left\{\begin{array}{ll}{l}^{j}& \quad if \;\; {x}_{i}^{j}\le {l}^{j}\\ {u}^{j}&\quad if \;\;{x}_{i}^{j}\le {u}^{j}\\ {x}_{i}^{j}&\quad otherwise\end{array}\right.$$

### Gradient based optimizer (GBO)

Gradient-based optimizer (GBO) is a metaheuristic optimization method that uses gradient information to direct the search process in optimization problems, as proposed by Ahmadianfar et al. in 2020^35^. The algorithm begins the search with a starting point and iteratively changes it depending on the gradient data of the goal function. The algorithm calculates the gradient of the objective function at the current solution for each iteration and utilizes it to update the solution by moving in the direction of the negative gradient. Each iteration's step size is determined by the learning rate parameter, and a random perturbation step is added to prevent getting stuck in local optima.

The gradient-based Newton's technique^35^ is the source of inspiration for the optimization algorithm GBO. The GBO algorithm consists of a set of vectors used to search the solution space and two main operators: the gradient search rule (GSR) and the local escaping operator (LEO). The GSR operator makes use of a gradient-based strategy to boost the algorithm's capacity for search space exploration and quicken the rate of convergence to a better solution. However, the LEO operator is made to assist the algorithm in escaping local optima and extending its search to additional areas of the solution space.

The GSR model's mathematical formulation is as follows:

38 $$\mathrm{GSR }=\mathrm{ rand}.{\upsigma }_{1}\frac{2 \cdot \Delta {\text{x}}.{{\text{x}}}_{{\text{n}}}}{({{\text{x}}}_{{\text{worst}}}-{{\text{x}}}_{{\text{best}}}+\upvarepsilon )}$$

The term "${\text{rand}}$" refers to a normally distributed random number, while "$\upvarepsilon$" represents a small value between 0 and 0.1. "${{\text{x}}}_{{\text{best}}}$" and "${{\text{x}}}_{{\text{worst}}}$" indicate the most favorable and unfavorable solutions obtained, respectively. "${\upsigma }_{1}$" is a coefficient used for balancing, which is mathematically defined as:

39 $${\upsigma }_{1}=2\cdot {\text{rand}}\cdot \mathrm{\alpha }-\mathrm{\alpha }$$

40 $$\mathrm{\alpha }=\left|\upbeta \cdot {\text{sin}}\left(\frac{3\uppi }{2}+{\text{sin}}\left(\upbeta \cdot \frac{3\uppi }{2}\right)\right)\right|$$

41 $$\upbeta ={\upbeta }_{{\text{min}}}+\left({\upbeta }_{{\text{max}}}-{\upbeta }_{{\text{min}}}\right)\times {\left(1-{\left(\frac{{\text{m}}}{{\text{M}}}\right)}^{3}\right)}^{2}$$

The values of ${\upbeta }_{{\text{min}}}$ and ${\upbeta }_{{\text{max}}}$, which are fixed at 0.2 and 1.2 respectively, along with the current iteration number '${\text{m}}$' and the total number of iterations '${\text{M}}$', are used to improve the utilization of the nearby region of '${{\text{x}}}_{{\text{n}}}$'. Additionally, a direction of movement (${\text{DM}}$) is included to aid in this enhancement, and it is defined as follows:

42 $$\mathrm{DM }=\mathrm{ rand}.{\upsigma }_{2}({{\text{x}}}_{{\text{best}}}-{{\text{x}}}_{{\text{n}}})$$

43 $${\upsigma }_{2}=2\cdot {\text{rand}}\cdot \mathrm{\alpha }-\mathrm{\alpha }$$

Using this method, the new position of the agent can be represented as:

44 $${{\text{x}}}_{{\text{n}}+1}= {{\text{x}}}_{{\text{n}}}-{\text{GSR}}+{\text{DM}}$$

**Figure 5:**
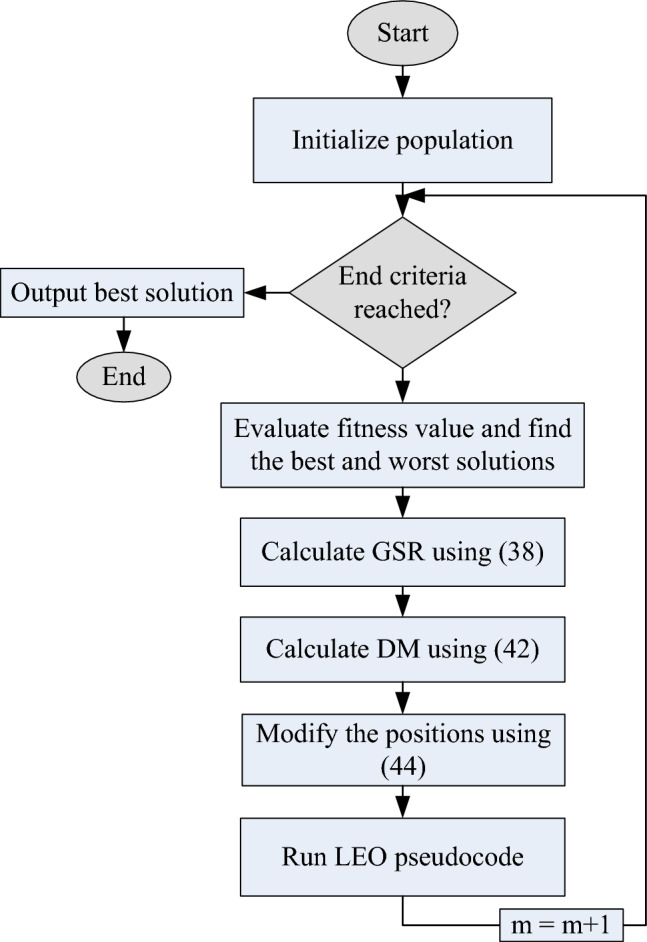
Flowchart of GBO.

The Local Event Operator (LEO) enables the GBO to escape from local optima. This step utilizes the positions created by the GBO, and the following pseudocode describes how it operates^35^:
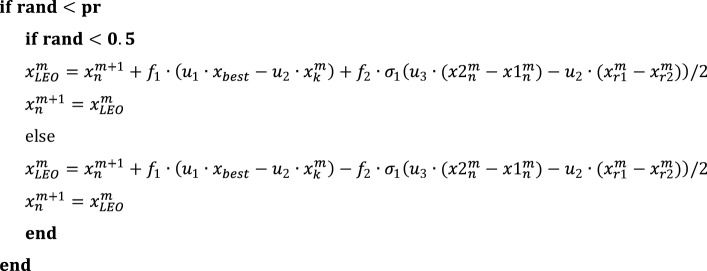


The given statement describes various variables and their definitions in the context of GBO algorithm. Two solutions generated by GBO are represented as ${x1}_{n}^{m}$ and ${x2}_{n}^{m}$ for a population of ${\text{m}}$ elements and ${\text{n}}$ optimization variables. Additionally, two random solutions are denoted by ${x}_{r1}^{m}$ and ${x}_{r2}^{m}$. The probability is represented as "${\text{pr}}$". The variables ${f}_{1}$ and ${f}_{2}$ are random numbers with different distributions. The former is a uniform random number between − 1 and 1, while the latter is a random number from a normal distribution with a mean of 0 and a standard deviation of 1.

45 $${{\text{u}}}_{1}= \left\{\begin{array}{ll}2\cdot {\text{rand}}& \quad {\upmu }_{1}<0.5\\ 1& \quad {\text{otherwise}}\end{array}\right.$$

46 $${{\text{u}}}_{2}, {{\text{u}}}_{3}= \left\{\begin{array}{ll}{\text{rand}}& \quad {\upmu }_{1}<0.5\\ 1& \quad {\text{otherwise}}\end{array}\right.$$

where ${{\text{u}}}_{1}$ is a number in the [0, 1] range and ${\text{rand}}$ is a random number between [0, 1]. Figure 5 depicts the operating system of the GBO. GBO is superior to other optimization methods in a number of ways. It simply needs to compute the gradient of the objective function, which can be done quickly for many different function types, making it computationally efficient. It is also simple to construct because it simply calls for fundamental operations like addition and multiplication. Finally, on a set of benchmark functions, GBO has demonstrated higher performance compared to other optimization algorithms in terms of convergence speed and solution quality.

### Northern Goshawk Optimization (NGO)

NGO is an optimization algorithm that is inspired by the hunting behavior of the Northern Goshawk bird. This algorithm was developed in 2016 by Seyedali Mirjalili and Andrew Lewis^36^. The Northern Goshawk bird is known for its speed, agility, and precision when hunting, and the NGO algorithm emulates this behavior by combining exploration and exploitation techniques to identify the optimal solution for a given problem.

The NGO algorithm starts by randomly initializing a population of candidate solutions, which are referred to as individuals. Each individual is represented as a vector of variables that can be adjusted to explore the solution space. The algorithm then evaluates the fitness of each individual by using an objective function. The search and attack operators are two critical components of the NGO algorithm. The search operator randomly adjusts an individual's variables to explore the solution space, while the attack operator selects the best individual and modifies its variables to exploit promising spots of the solution search area. The NGO algorithm also includes a memory mechanism known as the memory pool. This memory pool stores the best individuals found so far and guides the search and attack operators towards promising locations of the solution space.

The population-based NGO algorithm utilizes the searching behavior of Northern Goshawk birds as its guiding principle. Each member represents a candidate solution to the problem and is composed of a set of variable values. Mathematically, these members can be represented as vectors, and together, they constitute the population matrix of the method. The initialization of the population involves random placement of its members within the search space. The population matrix for the NGO technique is defined according to a specific formula as follow^36^:

47 $$X=\left[\begin{array}{c}{X}_{1}\\ \vdots \\ \begin{array}{c}{X}_{i}\\ \vdots \\ {X}_{N}\end{array}\end{array}\right]=\left[\begin{array}{ccc}{X}_{\mathrm{1,1}}& \dots & \begin{array}{ccc}{X}_{1,d}& \cdots & {X}_{1,m}\end{array}\\ \vdots & \ddots & \begin{array}{ccc}\vdots & \ddots & \vdots \end{array}\\ \begin{array}{c}{X}_{i,1}\\ \vdots \\ {X}_{N,1}\end{array}& \begin{array}{c}\cdots \\ \ddots \\ \cdots \end{array}& \begin{array}{c}\begin{array}{ccc}{X}_{i,d}& \cdots & {X}_{i,m}\end{array}\\ \begin{array}{ccc}\vdots & \ddots & \vdots \end{array}\\ \begin{array}{ccc}{X}_{N,d}& \cdots & {X}_{N,m}\end{array}\end{array}\end{array}\right]$$

The population of Northern Goshawks in the NGO algorithm is represented by variable $X$. Each member of the population is denoted by ${X}_{i}$ and is a candidate solution to the problem. The values of the jth variable determined by the ith candidate solution are represented as ${X}_{i,j}$. ${\text{N}}$ represents the number of population members, while m represents the number of variables in the problem.

Each individual in the population is a potential remedy to the issue, as was already said. As a result, each member of the population can be used to evaluate the problem's objective function. You can use (48) to represent these values for the objective function as a vector.

48 $$F(X)=\left[\begin{array}{c}{F}_{1}=F({X}_{1})\\ \vdots \\ \begin{array}{c}{F}_{i}=F({X}_{i})\\ \vdots \\ {F}_{N}=F({X}_{N})\end{array}\end{array}\right]$$

where ${F}_{i}$ is the objective function value obtained by the ith suggested solution and $F$ is the vector of achieved objective function values.

#### Phase 1: Prey identification (exploration)

During the initial phase of hunting, the Northern Goshawk selects its prey randomly and then swiftly attacks it. This approach enhances the exploration capability of the NGO algorithm by allowing for random selection of solutions from the search area. This process facilitates global search of the search area with the objective of defining the optimum region. The principles of this phase are mathematically represented as follow:

49 $${P}_{i}={X}_{k}, i =1, 2, ...., N, k =1, 2,....i-1, i+1,.....,N$$

50 $${X}_{i,j}^{new, P1}=\left\{\begin{array}{ll}{x}_{i,j}+r\left({p}_{i,j}-I{x}_{i,j}\right)& \quad {F}_{{P}_{i}}<{F}_{i}\\ {x}_{i,j}+r\left({{x}_{i,j} -p}_{i,j}\right)&\quad {F}_{{P}_{i}}\ge {F}_{i}\end{array}\right.$$

51 $${X}_{i}=\left\{\begin{array}{ll}{X}_{i}^{new, P1}& \quad {F}_{i}^{new, P1}<{F}_{i}\\ {X}_{i}& \quad {F}_{i}^{new, P1}\ge {F}_{i}\end{array}\right.$$

where Pi denotes the position of the prey for the ith Northern Goshawk, while ${F}_{P}$ represents the objective function value of the goshawk. The value of ${\text{k}}$ is a random natural number within the range of [1, N], and ${X}_{i}^{new, P1}$ is the new state of the ith proposed solution, with ${X}_{i,j}^{new, P1}$ denoting its jth dimension. ${F}_{i}^{new, P1}$ represents the objective function value of the proposed solution after the first phase of NGO. Additionally, the variables r and I are random numbers, with r within the range of [0, 1], and I either equal to 1 or 2.

#### Phase 2: Chase and escape operation (exploitation)

After the Northern Goshawk successfully attacks its prey, the prey will try to flee. The Northern Goshawk will continue to pursue the prey using a "tail and chase" strategy. Due to the Northern Goshawk's remarkable speed, it is able to pursue its prey in almost any situation until it captures it. Simulating this hunting behavior enhances the algorithm's ability to exploit local search spaces. In the proposed NGO algorithm, the hunting behavior is restricted to an attack position with a radius R. Figure 4 shows the chase process between the Northern Goshawk and the prey. Equations (52) to (54) mathematically model the concepts of this second phase.

52 $${X}_{i,j}^{new, P2}={x}_{i,j}+R(2r-1){x}_{i,j}$$

53 $$R =0.02\left(1-\frac{t}{T}\right)$$

54 $${X}_{i}=\left\{\begin{array}{ll}{X}_{i}^{new, P2}& \quad {F}_{i}^{new, P2}<{F}_{i}\\ {X}_{i}& \quad {F}_{i}^{new, P2}\ge {F}_{i}\end{array}\right.$$

This equation provides a representation of the variables employed to depict the new state and objective function value of the ith proposed solution in the second phase of the NGO algorithm. More specifically, ${X}_{i}^{new, P2}$ signifies the revised state of the proposed solution,${X}_{i,j}^{new, P2}$ specifies the adjusted value of the jth dimension of the solution, and ${F}_{i}^{new, P2}$ denotes the objective function value as per the second phase of the NGO algorithm. The present iteration and the highest possible amount of iterations are denoted, respectively, by the variables t and T.

**Figure 6:**
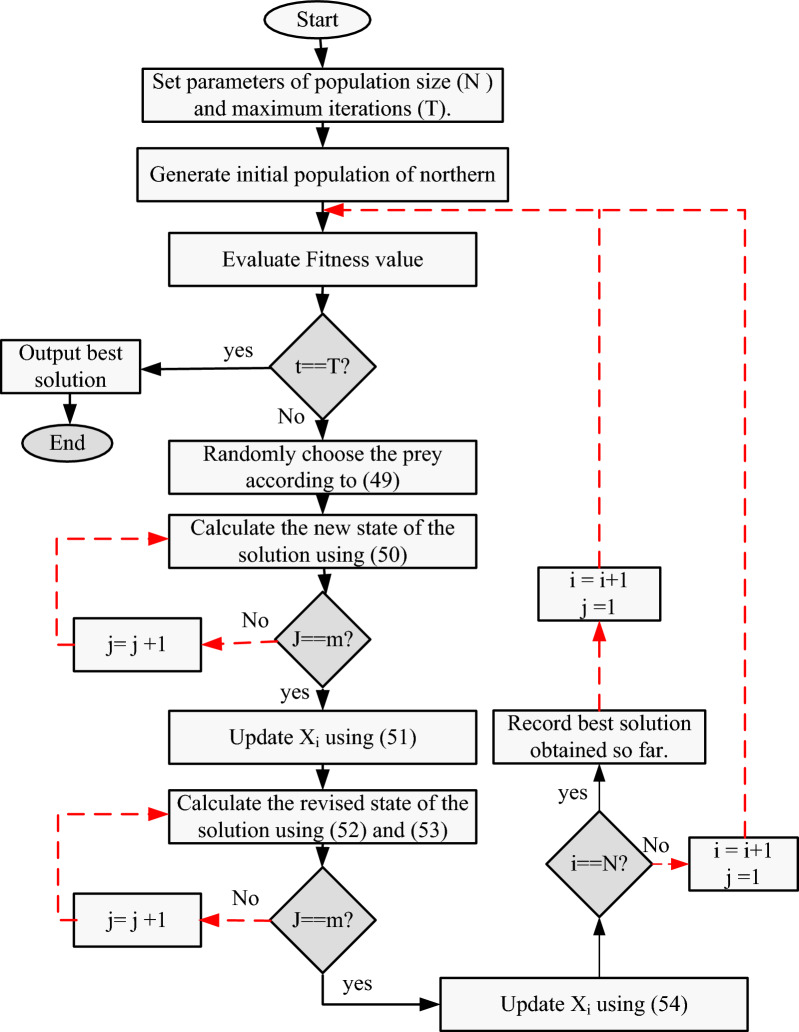
Flowchart of the NGO algorithm.

After updating all population members using the first and second stages of the NGO, and following one iteration of the method, the population members' new values, the objective function, and the best suggested solution are established. Up until the algorithm reaches its last iteration, this process is repeated. Once the full NGO method has been implemented, the best proposed solution that was discovered during the algorithm's iterations is regarded as a quasi-optimal solution for the specific optimization problem. The flowchart in Fig. 6 outlines the different stages of the NGO technique.

### Opposition-based flow directional algorithm (OFDA)

Liu and Lampinen created the Flow Direction Algorithm (FDA) in 2003 as an optimization technique^37^. It is a metaheuristic algorithm that takes its cues from how water flows through a landscape, always heading towards the lowest point. The FDA starts by generating a set of potential solutions, which are represented as points in a search region, and then assesses their effectiveness using an objective function. The algorithm then uses a series of steps to gradually improve the quality of the results.

The flow direction operator, which mimics water flowing towards the global minimum while moving downhill, is the FDA's main operation. Each point in the search space has its objective function's slope calculated by the algorithm, which then directs the points in that direction. The points are moved through this process repeatedly until they reach a local minimum. The FDA uses a diversity maintenance system to guarantee population diversity. It ensures that the population comprises a diversity of solutions that address different regions of the search space by randomly selecting individuals from the population and using mutation and crossover operators to create new solutions.

The FDA is a topic discussed in reference^46^. It takes inspiration from the flow of water in a drainage basin, which moves towards the outlet point with the lowest height. The direction of flow is influenced by neighboring flows and their slopes. In the FDA, each flow position, represented by ${Flow}_{X}$ and its corresponding height Flow fitness $f\left({Flow}_{X}\right)$, acts as a search agent for the parameter α flow. This parameter is initialized within the boundaries $[{\text{ub}},\mathrm{ lb}]$ in the drainage basin. The FDA estimates new flow positions in two ways. The first method assumes that a flow generates a β neighbor flow, ${{\text{Neighbor}}}_{X}$ (refer to Eq. (3) in^46^), while moving towards the drainage basin, and then updates its location, ${Flow}_{newX}$ (refer to Eq. (8) in^46^), based on the best neighbor flow. The second way updates the flow positions, ${Flow}_{newX}$ (refer to Eq. (9) in^46^), by assuming that the present flow encounters a random flow and changes its path. Finally, the flow's position is updated if it is better than the previous one, expressed as ${Flow}_{X(i)}$.

55 $${Flow}_{X(i)}=\left\{\begin{array}{ll}{Flow}_{newX(i)}& \quad f\left({Flow}_{newX(i)}\right)<f\left({Flow}_{X(i)}\right)\\ {Flow}_{X(i)}& \quad otherwise\end{array}, \forall i \in \left[1, \alpha \right]\right.$$

The height of ${Flow}_{newX(i)}$ is denoted by $f\left({Flow}_{newX(i)}\right)$. The algorithm updates the flow position iteratively until it converges to the optimal solution or reaches the maximum iteration, ${{\text{Max}}}_{I{\text{ter}}}$. The FDA has shown exceptional performance on benchmark functions and has yielded better results for real-world engineering design problems. Further details about the FDA can be found in reference^46^.

As previously mentioned, the FDA algorithm updates its solutions in the search space based on random neighbor flows or other random flows. However, this approach may lead the flow to a local optimal solution, which could be a trap. To avoid this issue, the opposition-based learning (OBL) method can be utilized^47^. OBL helps in the search process in both directions. Let, ${Flow}_{X(i)}$ be a flow in the d-dimensional search space with a range of $\left[LB, UB\right]$, where

56 $$\begin{aligned}{Flow}_{X(i)} &=\left\{{Flow}_{X(i, 1)}, {Flow}_{X(i, 2)},...., {Flow}_{X(i, d)}\right\} \\ {\text{LB}} &= \left\{{\text{lb}}(1),\mathrm{ lb}(2),......,\mathrm{ lb}({\text{d}})\right\} \\ {\text{UB}} &= \left\{{\text{ub}}(1),\mathrm{ ub}(2),......,\mathrm{ ub}({\text{d}})\right\}\end{aligned}$$

The opposite flow can then be determined as:

57 $${OFlow}_{X(i)}=\left\{{OFlow}_{X(i, 1)}, {OFlow}_{X(i, 2)},...., {OFlow}_{X(i, d)}\right\}$$

58 $${OFlow}_{X(i,j)} ={\text{ub}}({\text{j}}) +{\text{lb}}({\text{j}}) -{Flow}_{X(i,j)}, , \forall i \in \left[1, \alpha \right] and , \forall j \in \left[1, d\right]$$

The opposition-based flow directional algorithm (OFDA) employs a selection mechanism, which is described below, to update its solutions in the search space.

59 $${Flow}_{X(i)}=\left\{\begin{array}{ll}{OFlow}_{X(i)}& \quad f\left({OFlow}_{X(i)}\right)<f\left({Flow}_{X(i)}\right)\\ {Flow}_{X(i)}& \quad otherwise\end{array}, \forall i \in \left[1, \alpha \right]\right.$$

**Figure 7:**
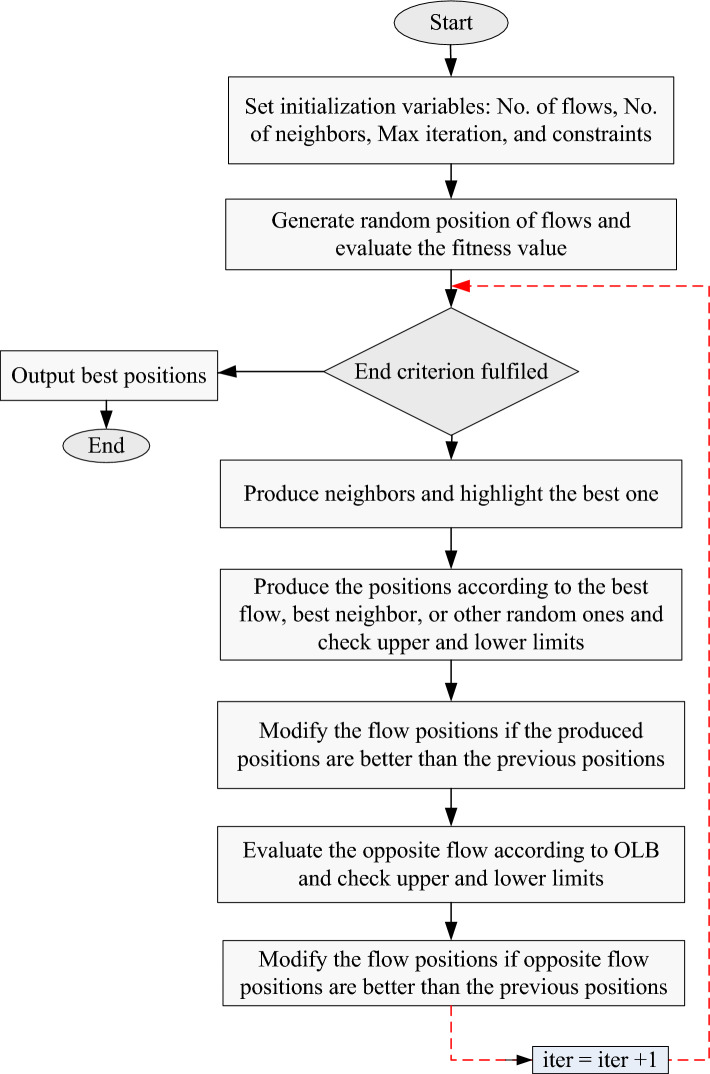
Flowchart of OFDA.

Based on its opposing flow, the aforementioned equation modifies the flow position in OFDA. $f\left({OFlow}_{X(i)}\right)$ denotes the height of ${OFlow}_{X(i)}$. The deployment of OFDA is shown in Fig. 7, which explains this update procedure.

## Results and discussion

**Figure 8:**
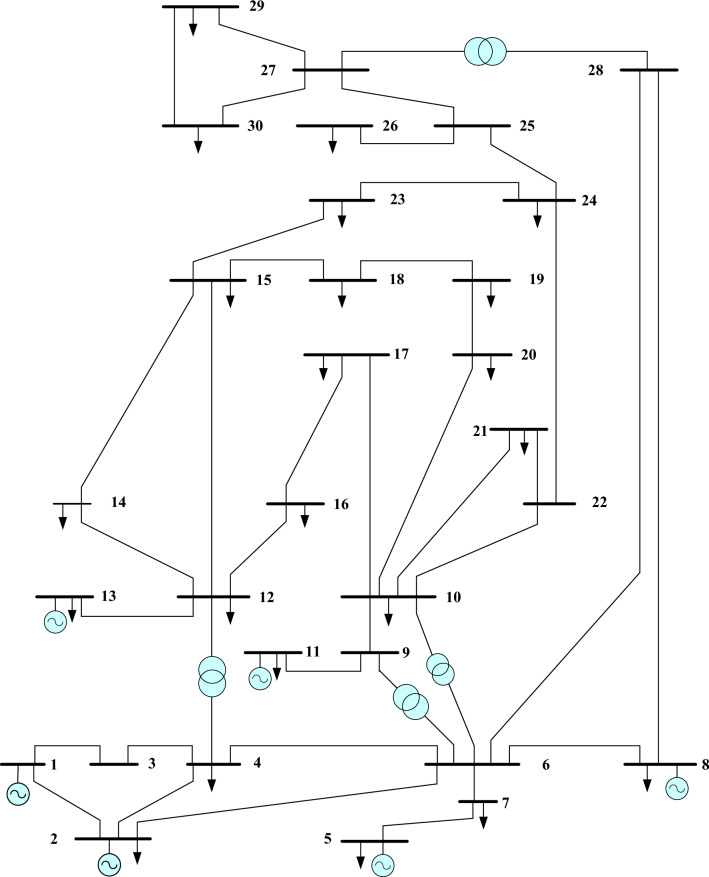
Single line diagram of the IEEE 30 Bus system.

The OPF problem is solved by implementing the suggested FCS, SSA, DCCS, GBO, NGO, and OFDA algorithms. In this study, the IEEE 30 Bus test system has been used to examine 7 different case studies. The produced programs for this paper were created in MATLAB and used on an i5 computer running at 2.20 GHz and 4.00 GB of RAM. Using all suggested techniques, the optimal power flow program is implemented 30 times for each case. The maximum number of iterations is adjusted at 100 iterations for all algorithms and the population size equals to 40 agents. The single line diagram of the IEEE 30 Bus system is presented in Fig. 8. The primary characteristics of the IEEE 30-bus test system are listed in Table 1 and its total power capacity is 435.0 MW and the reader can get specific information about this test system from^48^.

**Table 1:** The fundamental features of the IEEE 30-bus test system.

Features	Value	Description
No. of buses	30	^48^
No. of branches	41	^48^
No. of generators	6	Buses: 1, 2, 5, 8, 11 and 13
No. of shunt compensators	9	Buses: 10, 12, 15, 17, 20, 21, 23, 24 and 29
No. of transformers	4	Branches: 11, 12, 15 and 36
No. of control variables	24	–

The application of the different algorithms for solving the OPF problem of the IEEE 30 Bus system has occurred by considering the searching variable can be described as follows:

$$x= [P1, P2, P5. P8, P11, P13, V1, V2, V5, V8, V11, V13, T11, T12, T15, T36, QC10, QC12, QC15, QC17, QC20, QC21, QC23, QC24, QC27]$$

And the objective function is one of those presented in “Problem formulation” section considering the system constraints.

### Case-1

**Figure 9:**
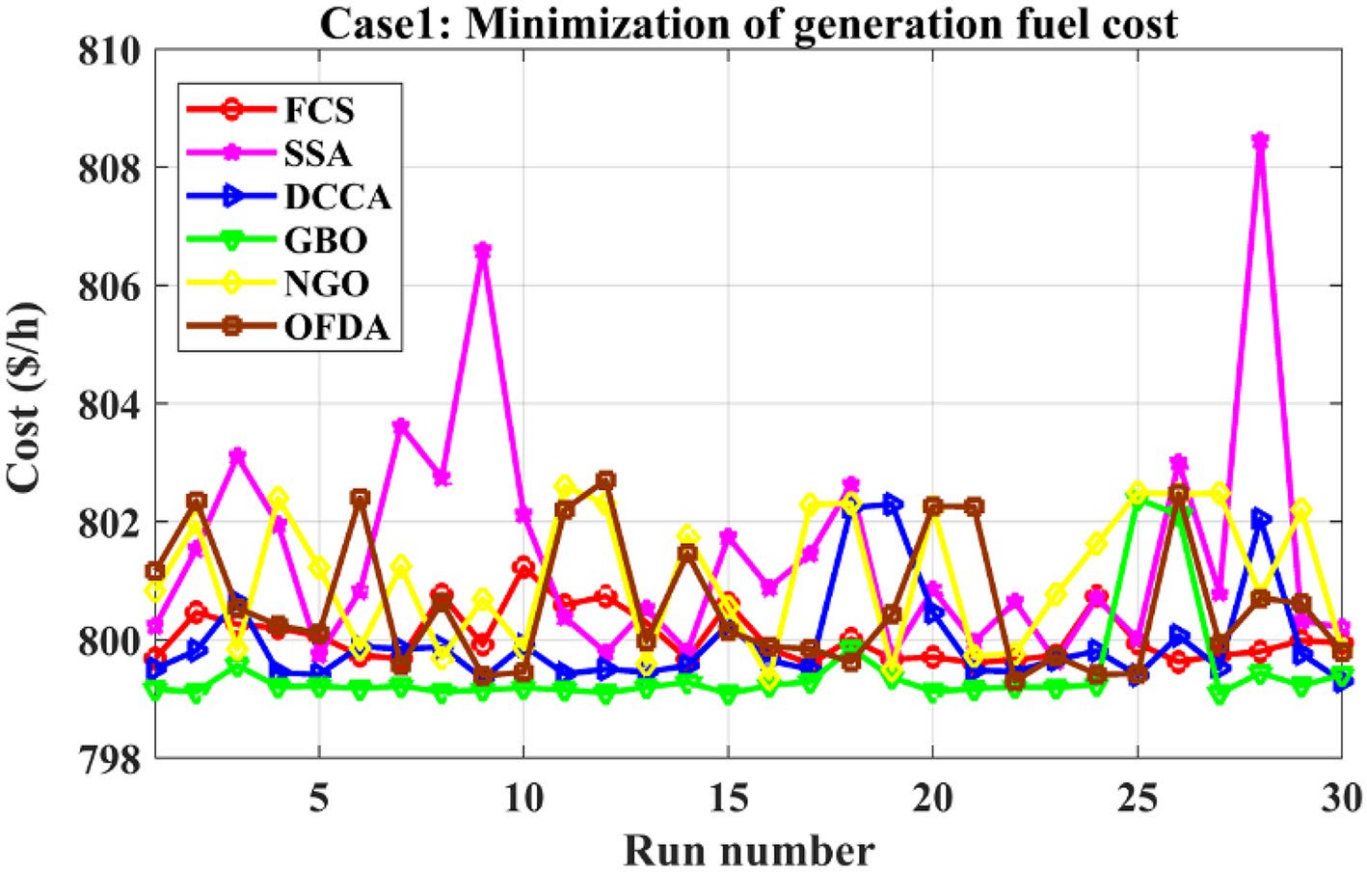
Variation of the objective function over the 30 runs for case 1.

**Figure 10:**
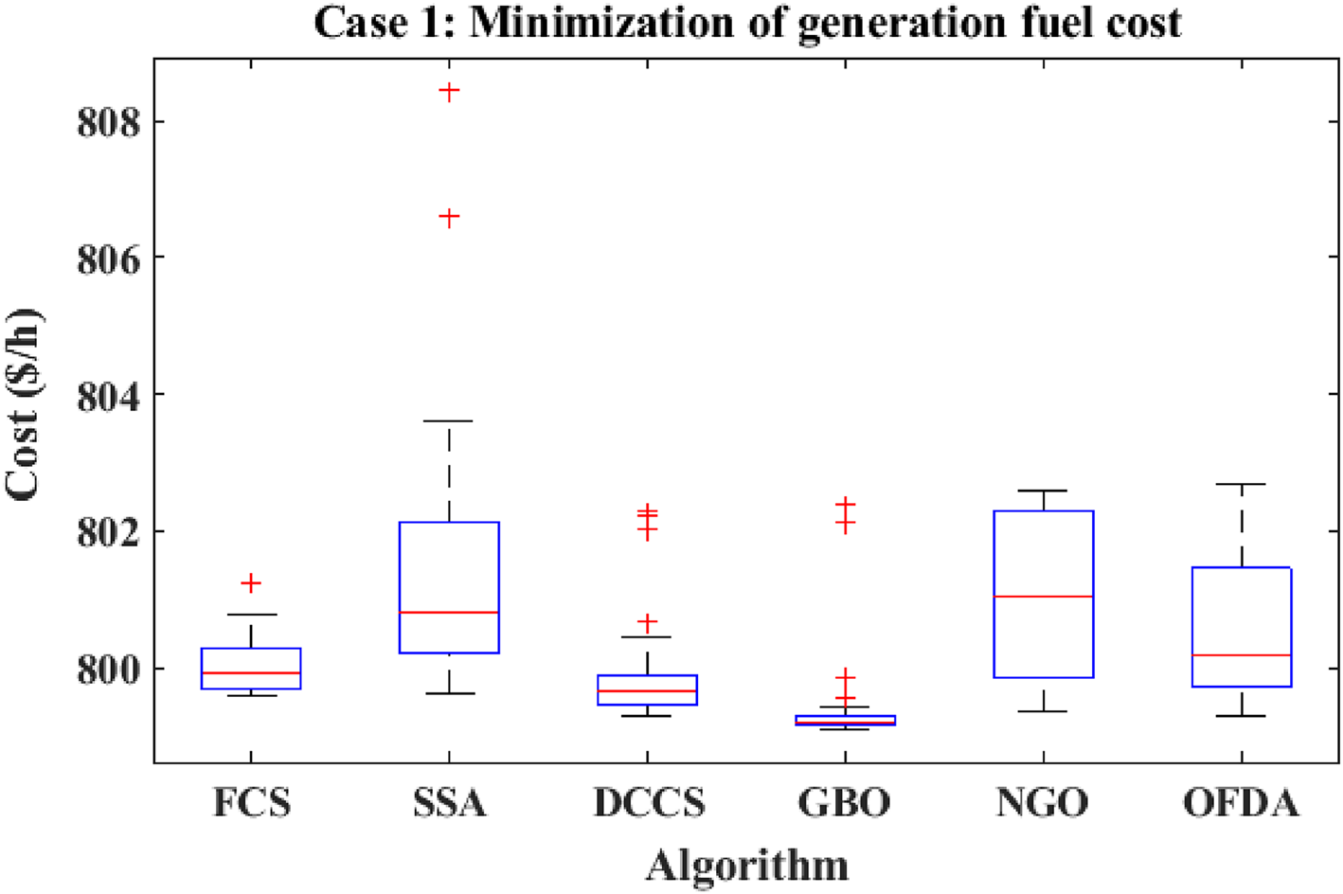
Boxplot for the results of the objective function of case 1.

**Figure 11:**
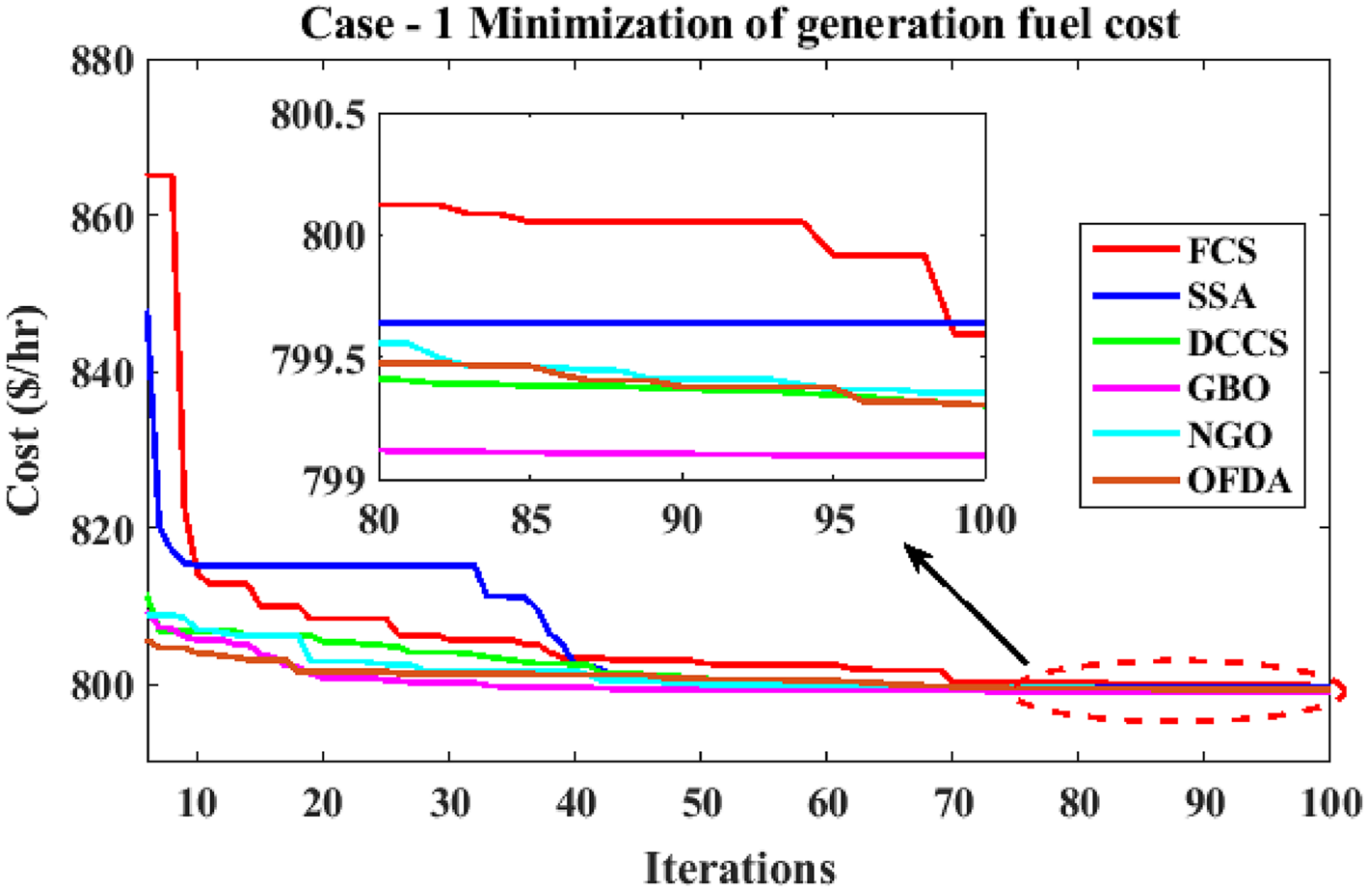
Variation of the generation fuel cost for case 1.

**Figure 12:**
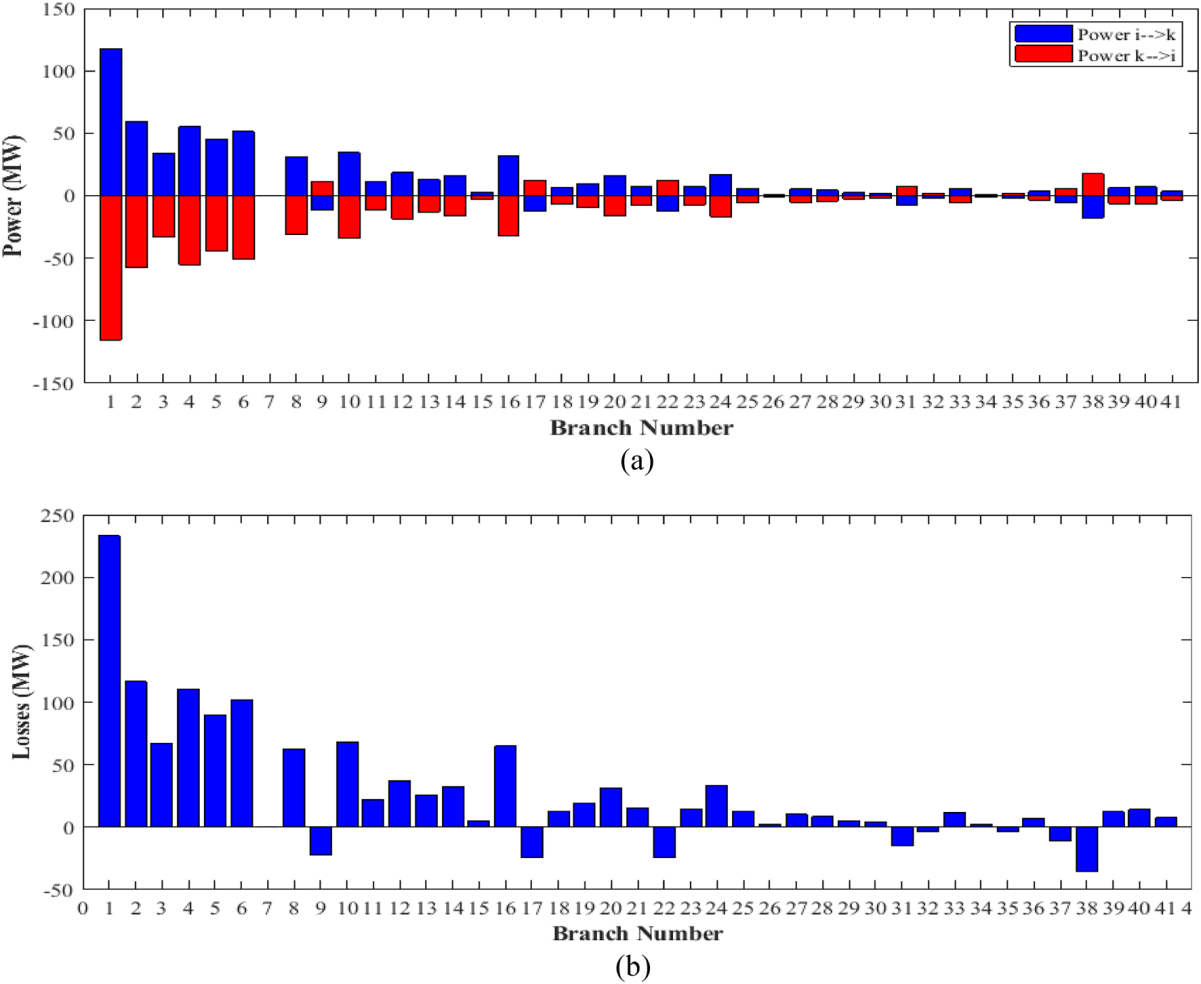
Active power flow and losses in the branches of the system for case 1.

**Figure 13:**
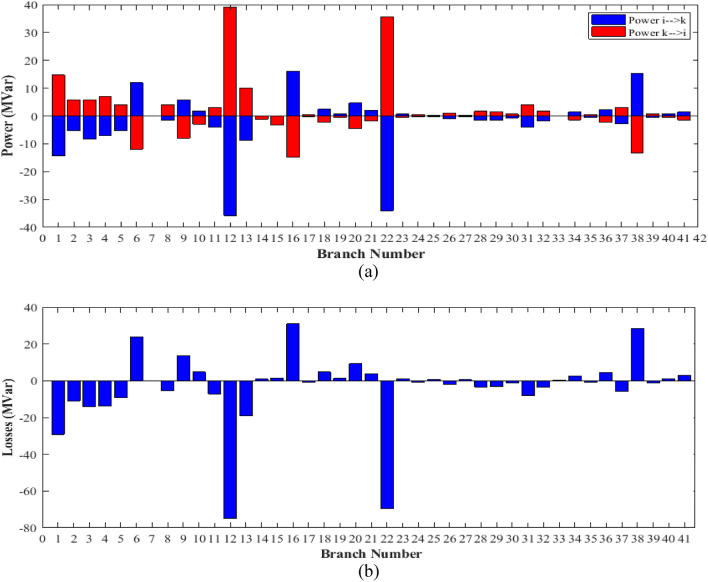
Reactive power flow and losses in the branches of the system for case 1.

**Figure 14:**
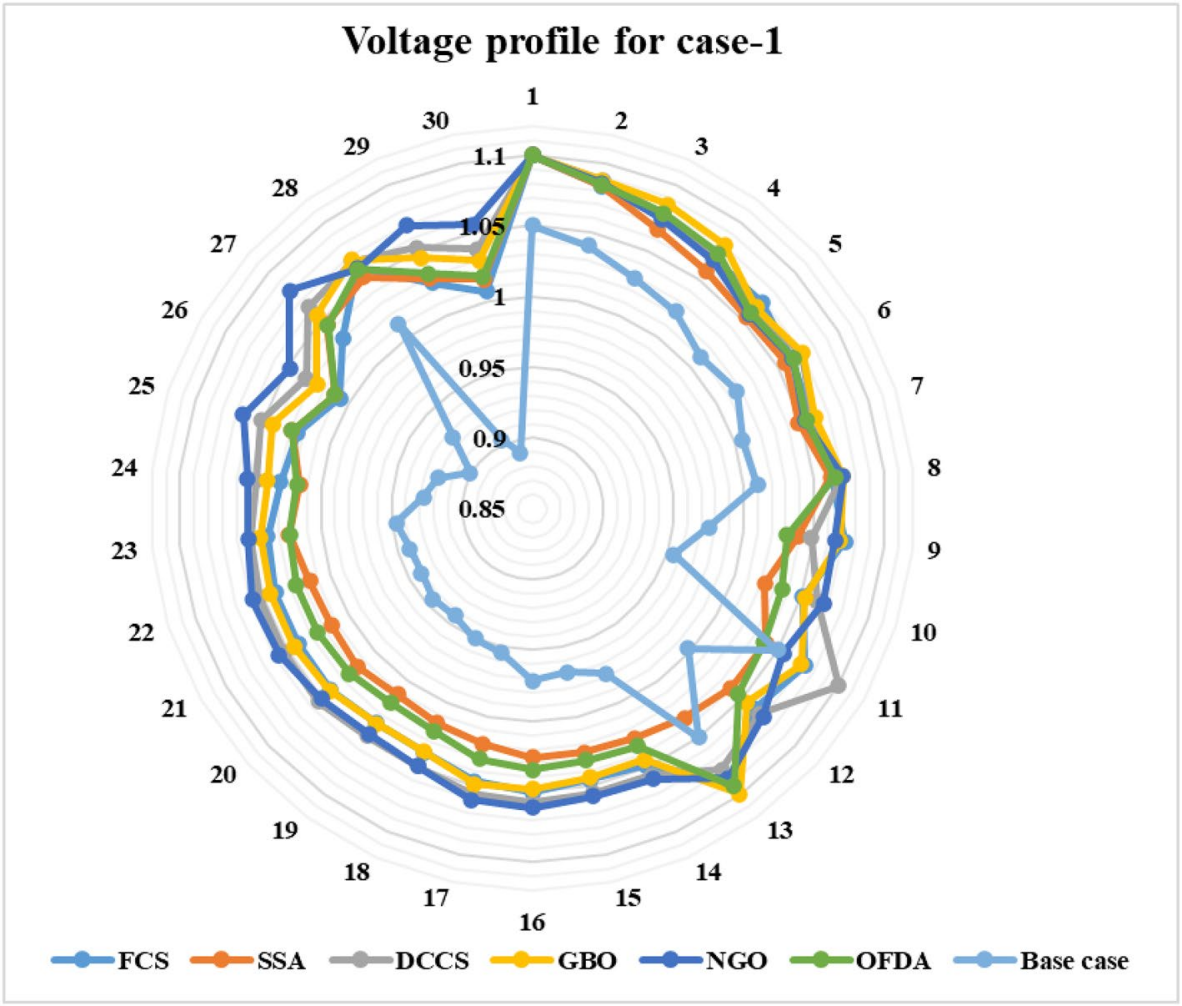
Voltage profile improvement for case 1.

The proposed FCS, SSA, DCCS, GBO, NGO, and OFDA optimization methodologies have been implemented for 30 individual runs to address the optimization problem of OPF incorporating minimization of the fuel generation cost from the six generators of the system. The obtained results of the best value of the objective function in each run is recorded an presented in the graph shown in Fig. 9. Statistical study including the best and worst values of the fuel cost as well as the mean, standard deviation, and the root mean square error based on the recorded values of the cost function during the 30 individual runs has been conducted and the results are listed in Table 2. The elapsed time. Friedman's ANOVA Table and Wilcoxon signed rank test have been performed to evaluate the optimization algorithms. The results of Friedman's ANOVA Table show the p-value of 0.0015989 for columns has been obtained. With a p-value of 0.0015989, Friedman's ANOVA test rejects the null hypothesis at a standard 5% significance level. The values of meanranks are [3.5000 4.6667 2.5000 1 4.6667 4.6667] for [FCSSSADCCSGBONGOOFDA] respectively, which confirm the robustness of the GBO algorithm. Moreover, the Wilcoxon signed rank test results have been shown in Table 2. The results prove that the algorithms with the given p-values reject the null hypothesis with a rank h of 1. A boxplot based on the 30 values for the first case study obtained from each algorithm is provided in Fig. 10. The reader can notice that the GBO optimization technique provided promising results among the six proposed methods. The stability of the algorithm is proved by the narrow range in which the objective function is varied during the 30 runs of the optimization program. The minimum value of the fuel cost obtained from GBO is 799.0938 $/h compared to 799.5921 $/h for FCS, 799.6411 $/h for SSA, 799.3019 $/h for DCCS, 799.3542 $/h for NGO, and 799.3041 $/h for OFDA. The good convergence characteristics of the GBO is presented in the graph of the convergence curves of the proposed algorithms presented in Fig. 11. The findings of the optimization process regarding the values of the control variables, fuel cost, voltage deviation, voltage stability index $${\text{Lmax}}$$, active power losses, and reactive power losses for case 1 compared to the base case are provided in Table 3. The total fuel generation cost based on GBO algorithm has been reduced by 11.404% from the base case compared with 11.348% for FCS, 11.343% for SSA, 11.381% for DCCS, 11.375% for NGO, and 11.3805% for OFDA. The active power flow in the 41 branches of the IEEE 30 Bus system is presented in Fig. 12a and the power losses in each branch is sketched in Fig. 12b. Similarly, the reactive power flow is presented in Fig. 13a and the reactive power losses in each branch is sketched in Fig. 13b. The impact of the optimization process on the voltage profile of the PQ buses of the system is presented in Fig. 14. Finally, the active and reactive power balance based on the results of the six proposed algorithms is provided in Table 4.

**Table 2:** Statistical study for case 1.

	Min.	Max.	Mean	SD	RMSE	P	h	Elapsed Time
FCS	799.5921	801.2312	800.0527	44.0055	0.6320	0.03125	1	41.90178
SSA	799.6411	808.4531	801.4684	200.8181	2.6902	0.03125	1	23.06625
DCCS	799.3019	802.2953	799.9549	82.6775	1.0427	0.03125	1	44.01723
GBO	799.0938	802.3854	799.4441	78.0080	0.8432	0.03125	1	20.108
NGO	799.3542	802.5991	801.0752	115.3513	2.0611	0.03125	1	11.04517
OFDA	799.3041	802.7016	800.6034	112.2498	1.7047	0.03125	1	72.19611

**Table 3:** Optimized values of the control variable for case 1.

	0	Min	Max	FCS	SSA	DCCS	GBO	NGO	OFDA
P1	99.223	50	200	178.089	177.714	176.094	176.835	179.474	177.865
P2	80	20	80	49.7415	48.7566	49.0451	48.8974	47.2824	47.3489
P5	50	15	50	21.9470	21.4155	21.0541	20.9864	20.6418	20.8235
P8	20	10	35	19.3329	19.5943	22.1354	21.3621	21.7137	21.7796
P11	20	10	30	10.8316	12.8999	11.6990	12.0124	11.2290	12.1230
P13	20	12	40	12.4048	12.0000	12.0004	12.0192	12.0000	12.2852
V1	1.05	0.95	1.1	1.1000	1.1000	1.1000	1.1000	1.1000	1.0997
V2	1.04	0.95	1.1	1.0832	1.0839	1.0876	1.0871	1.0852	1.0843
V5	1.01	0.95	1.1	1.0683	1.0531	1.0597	1.0627	1.0559	1.0573
V8	1.01	0.95	1.1	1.0628	1.0618	1.0696	1.0707	1.0706	1.0647
V11	1.05	0.95	1.1	1.0721	1.0407	1.0999	1.0695	1.0545	1.0388
V13	1.05	0.95	1.1	1.0876	1.0328	1.0778	1.0993	1.0855	1.0920
T11	1.078	0.9	1.1	0.9533	0.9895	1.0998	0.9731	0.9885	1.0455
T12	1.069	0.9	1.1	1.0306	1.0819	0.9000	1.0557	0.9179	0.9332
T15	1.032	0.9	1.1	1.0290	0.9802	1.0053	1.0997	1.0012	1.0764
T36	1.068	0.9	1.1	1.0316	0.9776	0.9882	0.9993	0.9603	0.9922
QC10	0	0	5	0.0000	2.4346	4.9479	4.7751	5.0000	4.1630
QC12	0	0	5	5.0000	2.4042	1.6721	4.5987	1.3774	0.6812
QC15	0	0	5	2.2338	3.9215	4.9557	2.8771	5.0000	4.8785
QC17	0	0	5	2.5591	3.4281	1.8720	4.9941	0.8881	1.0800
QC20	0	0	5	4.0152	4.0575	4.9131	4.2086	0.0000	1.8685
QC21	0	0	5	4.2844	2.7144	3.2527	4.9122	2.3643	2.0540
QC23	0	0	5	2.5331	4.4800	1.7569	4.6539	0.0000	0.5745
QC24	0	0	5	3.1387	4.7520	4.9638	4.7523	5.0000	1.6675
QC27	0	0	5	5.0000	0.8398	2.7234	3.1399	2.6404	2.5830
Fuel cost ($/h)	**901.951**	**–**	**–**	**799.592**	**799.641**	**799.301**	**799.093**	**799.354**	**799.304**
Active power losses (MW)	5.8219	**–**	**–**	8.7456	8.7521	8.6288	8.6028	8.7701	8.7487
Reactive power losses (MVar)	− 4.6066	**–**	**–**	0.7157	0.5970	1.1977	3.4600	6.4295	2.7367
Voltage deviation	1.1496	**–**	**–**	0.1276	0.1274	0.1234	0.1175	0.1184	0.1221
Lmax	0.17233	**–**	**–**	1.0738	0.9689	1.3371	1.7705	1.8149	1.5129

Significant values are in bold.

**Table 4:** Active and reactive power balance for case 1.

Case 1	Active power balance			Reactive power balance				
Method	Load (MW)	Generation (MW)	Loss (MW)	Load (MVar)	Generation (MVar)	Compensation (MVar)	Charging (MVar)	Loss (MVar)
FCS	283.40	292.3475	8.9480	126.20	97.1791	28.7642	3.5673	3.3105
SSA	283.40	292.3812	8.9814	126.20	97.3457	29.0319	0.8354	1.0130
DCCS	283.40	292.0282	8.6288	126.20	98.7926	31.0575	− 2.4524	1.1977
GBO	283.40	292.1129	8.7136	126.20	88.5646	38.9119	1.2094	2.4858
NGO	283.40	292.3409	8.9414	126.20	103.3868	22.2703	4.4139	3.8711
OFDA	283.40	292.2261	8.8266	126.20	108.7985	19.5503	− 1.2632	0.8856
Base case	283.40	289.2225	5.8225	126.20	121.5936	0.0000	0.0000	− 4.6063

### Case-2

**Figure 15:**
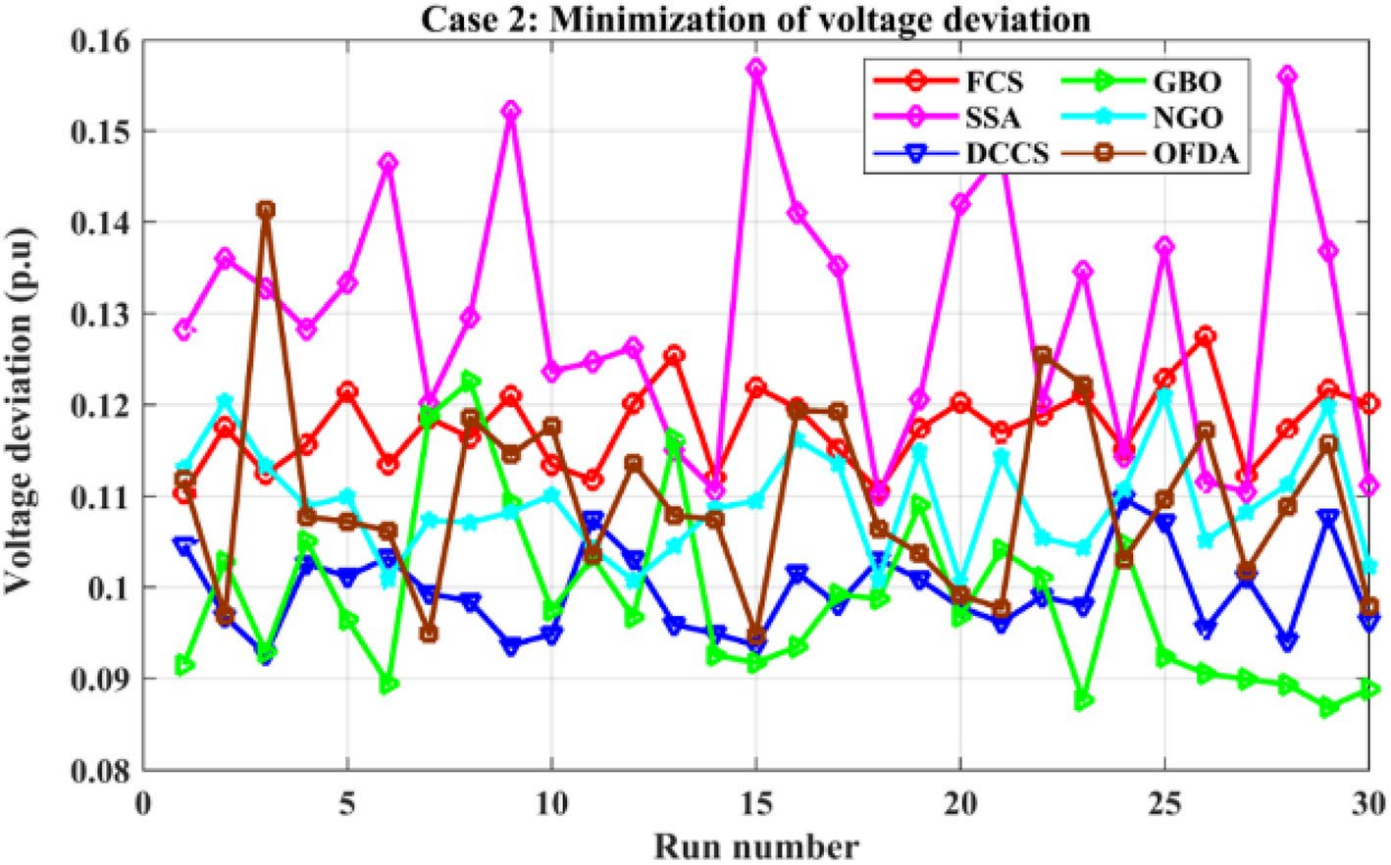
Variation of the objective function over the 30 runs for case 2.

**Figure 16:**
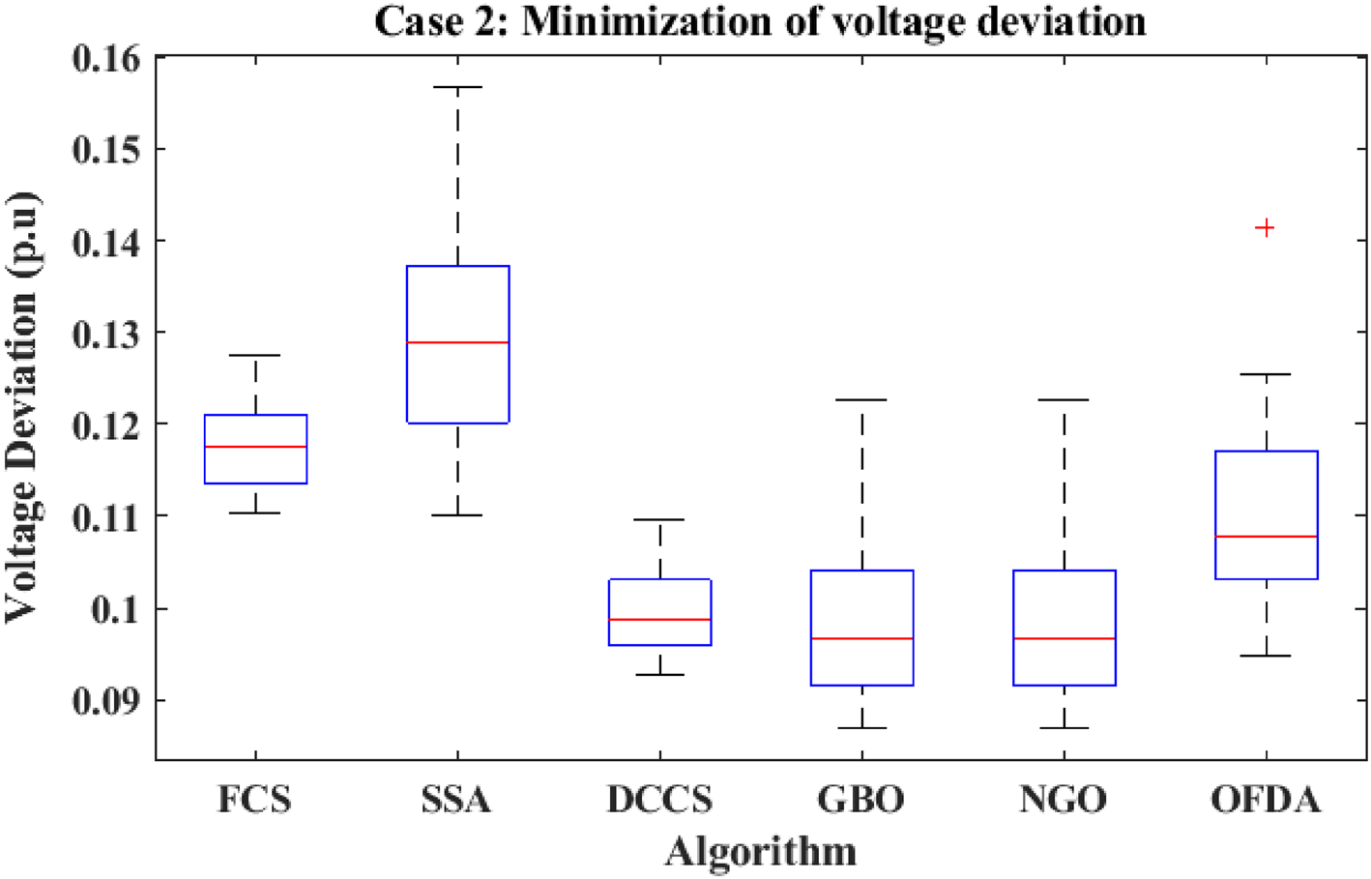
Boxplot for the results of the objective function of case 2.

**Figure 17:**
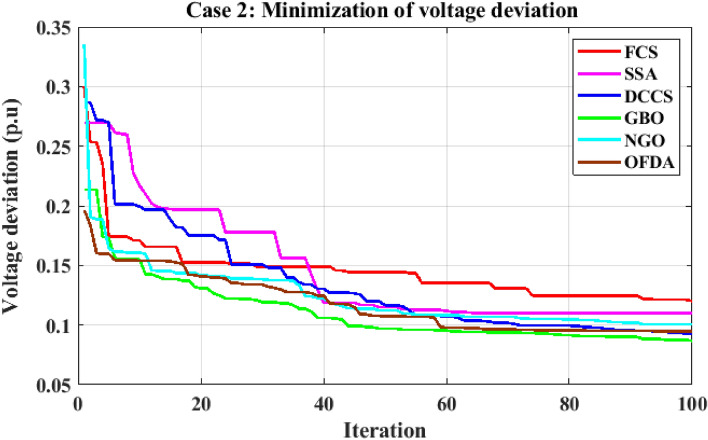
Variation of the total voltage deviation of case 2.

**Figure 18:**
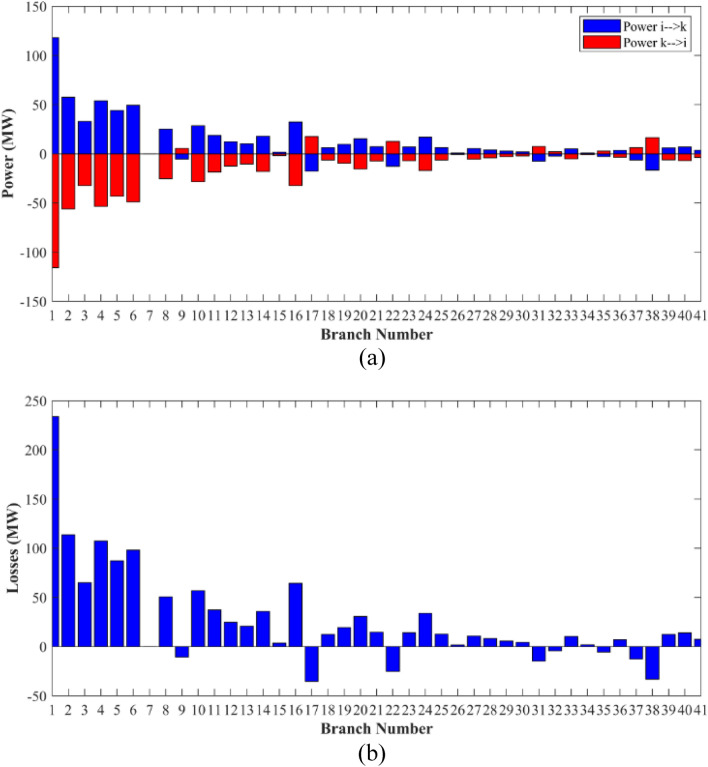
Active power flow and losses in the branches of the system for case 2.

**Figure 19:**
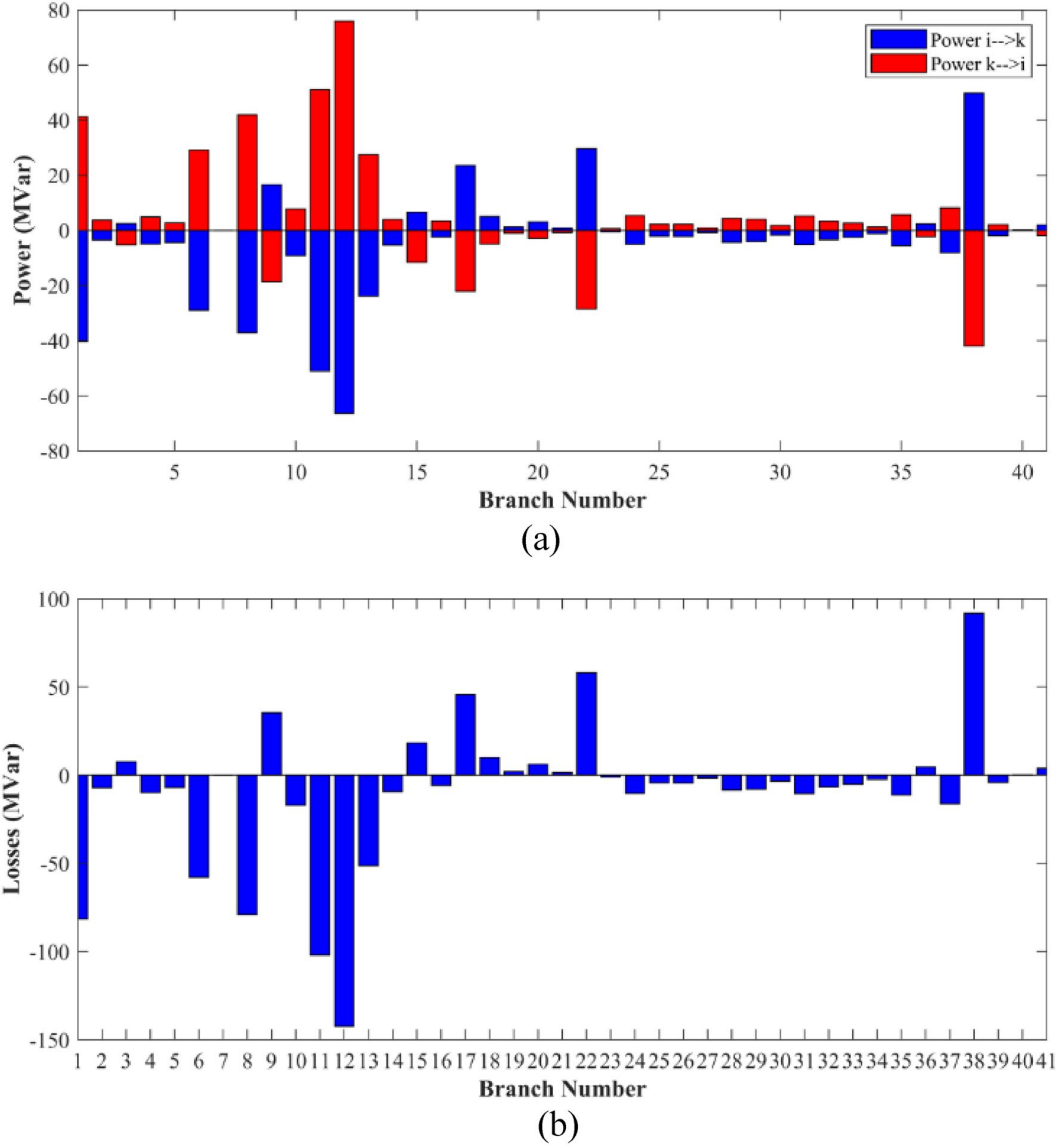
Reactive power flow and losses in the branches of the system for case 2.

**Figure 20:**
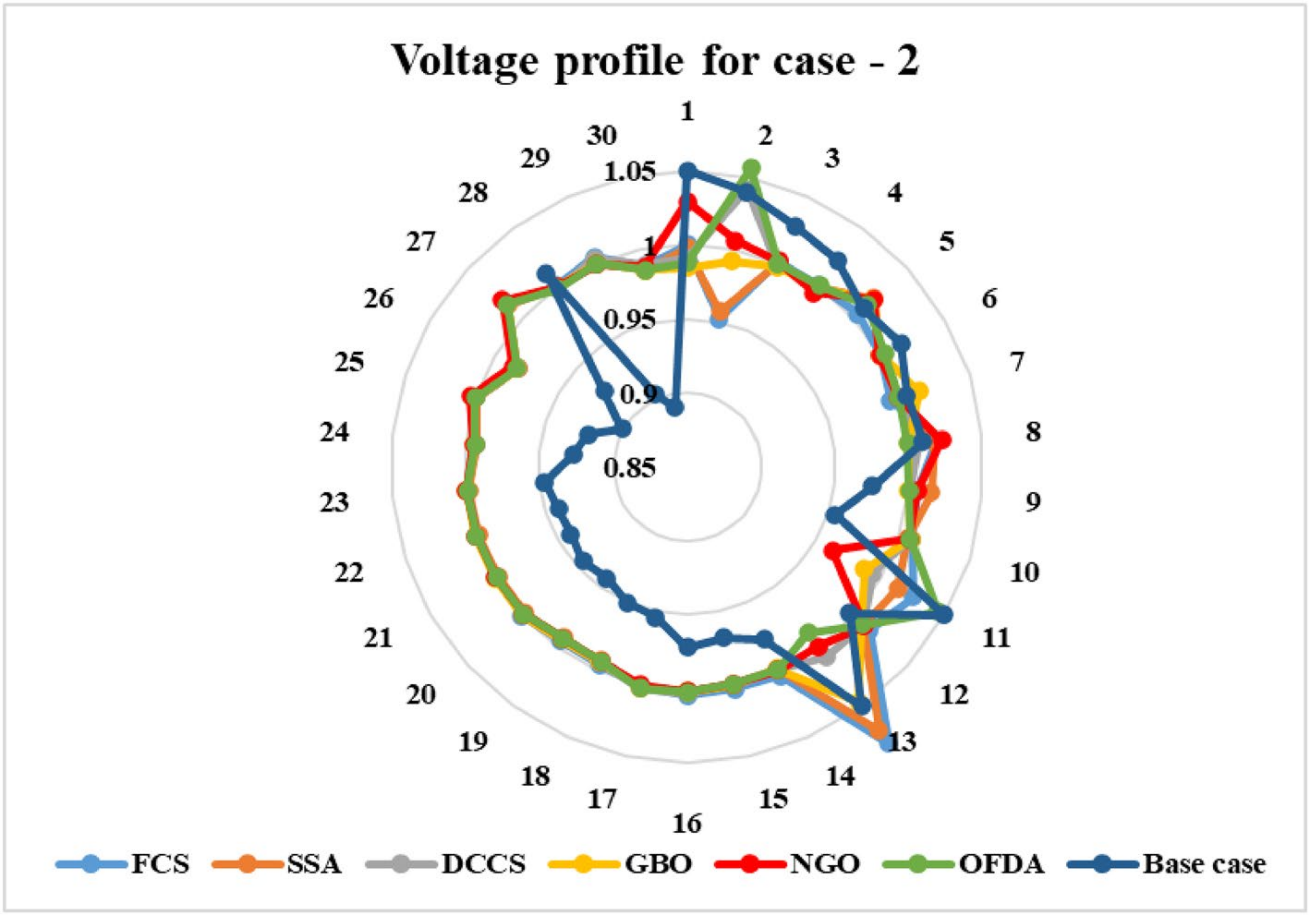
Voltage profile improvement for case 2.

The proposed algorithms have been implemented for 30 individual runs to address the optimization problem of OPF incorporating the objective function of case 2. The obtained results of the best value of the voltage deviation in each run is recorded an presented in the graph shown in Fig. 15. Statistical study has been conducted and the results are listed in Table 5. A boxplot based on the 30 values of the total voltage deviation is sketched in Fig. 16. Also, in this case, the GBO optimization technique provided the best performance compared with the others. The minimum value of the total voltage deviation obtained from GBO is 0.0.08682 p.u compared to 0.11033 p.u for FCS, 0.11010 p.u for SSA, 0.09262 p.u for DCCS, 0.10048 p.u for NGO, and 0.09474 p.u for OFDA. The variation of the total voltage deviation over the 100 iterations of the best runs for all algorithms presented in Fig. 17. The results of the optimization process for case 2 compared to the base case are provided in Table 6. The active power flow in the 41 branches is presented in Fig. 18a and the power losses in each branch is sketched in Fig. 18b. Similarly, the reactive power flow is presented in Fig. 19a and the reactive power losses in each branch is sketched in Fig. 19b. The impact of the optimization process on the voltage profile of the PQ buses of the system is presented in Fig. 20. Finally the active and reactive power balance based on the results of the six proposed algorithms is provided in Table 7.

**Table 5:** Statistical study for case 2.

	Min.	Max.	Mean	SD	RMSE
FCS	0.11033	0.12752	0.11761	0.43936	0.00847
SSA	0.11010	0.15682	0.12976	1.39103	0.02396
DCCS	0.09262	0.10972	0.09962	0.46813	0.00838
GBO	0.086821	0.122564	0.098646	0.009434	0.099081
NGO	0.10048	0.12095	0.10919	0.58323	0.01043
OFDA	0.09474	0.14133	0.10972	1.03139	0.01808

**Table 6:** Optimization results for case 2.

	0	Min	Max	FCS	SSA	DCCS	GBO	NGO	OFDA
P1	99.2230	50	200	155.326	121.401	169.815	130.079	114.530	100.407
P2	80	20	80	23.3078	41.7280	56.3157	74.0568	79.9904	67.9528
P5	50	15	50	15.8271	49.4738	18.6378	22.7177	39.1229	43.1018
P8	20	10	35	31.3588	32.1339	22.8388	28.0304	26.3953	31.5723
P11	20	10	30	28.8960	18.7324	15.4396	25.4183	18.5095	26.1240
P13	20	12	40	40.0000	28.3541	13.3247	12.0328	12.0000	23.5674
V1	1.05	0.95	1.1	0.9998	0.9978	0.9906	0.98482	1.0289	0.9879
V2	1.04	0.95	1.1	0.9520	0.9569	1.0422	0.99224	1.0064	1.0563
V5	1.01	0.95	1.1	1.0044	1.0195	1.0168	1.01780	1.0190	1.0133
V8	1.01	0.95	1.1	1.0188	1.0178	1.0073	1.01460	1.0228	0.9994
V11	1.05	0.95	1.1	1.0257	1.0141	0.9940	0.98800	0.9630	1.0452
V13	1.05	0.95	1.1	1.0808	1.0701	1.0088	1.04645	0.9997	0.9882
T11	1.078	0.9	1.1	1.0174	0.9645	1.0052	1.00188	0.9548	1.0634
T12	1.069	0.9	1.1	0.9000	0.9851	0.9001	0.91261	0.9145	0.9001
T15	1.032	0.9	1.1	1.1000	1.0928	0.9698	1.05714	0.9403	0.9431
T36	1.068	0.9	1.1	0.9695	0.9658	0.9620	0.96555	0.9538	0.9631
QC10	0	0	5	3.7859	2.4814	3.4653	4.99996	0.6215	1.0362
QC12	0	0	5	2.5615	0.1425	0.9453	4.99988	0.0236	1.7656
QC15	0	0	5	3.7009	4.9165	3.2202	4.99961	2.0516	4.0645
QC17	0	0	5	0.0000	4.5412	2.3189	1.95423	0.0534	3.4869
QC20	0	0	5	4.9377	4.8470	4.9870	4.99998	4.9772	4.9744
QC21	0	0	5	1.9060	4.9878	3.2708	4.99140	4.9704	4.2112
QC23	0	0	5	3.7751	4.2073	4.9120	4.99992	4.9787	4.7092
QC24	0	0	5	4.1808	4.9882	5.0000	4.92126	4.9839	4.9977
QC27	0	0	5	3.8861	2.3566	2.3729	2.04973	0.4564	2.0986
Fuel cost ($/h)	901.9516	–	–	860.583	887.197	817.439	833.940	860.282	888.502
Active power losses (MW)	5.8219	–	–	11.4017	8.4948	13.0723	9.0257	7.2256	9.3931
Reactive power losses (MVar)	− 4.6066	–	–	20.6685	10.2808	20.4528	10.8586	3.6694	7.7366
Voltage deviation	**1.1496**	–	–	**0.1201**	**0.1112**	**0.0963**	**0.0888**	**0.1023**	**0.0980**
Lmax	0.17233	–	–	0.1367	0.1366	0.1367	0.1361	0.1393	0.1373

Significant values are in bold.

**Table 7:** Active and reactive power balance for case 2.

Case 2	Active power balance			Reactive power balance				
Method	Load (MW)	Generation (MW)	Loss (MW)	Load (MVar)	Generation (MVar)	Compensation (MVar)	Charging (MVar)	Loss (MVar)
FCS	283.40	294.7157	11.4017	126.2000	118.3478	28.7341	− 0.2133	20.6685
SSA	283.40	291.8237	8.4948	126.2000	99.9259	33.4685	3.0864	10.2808
DCCS	283.40	296.3721	13.0723	126.2000	112.7062	30.4924	3.4543	20.4528
GBO	283.40	292.3360	9.0257	126.2000	95.4843	38.9160	2.6583	10.8586
NGO	283.40	290.5490	7.2256	126.2000	103.7794	23.1167	2.9733	3.6694
OFDA	283.40	294.7157	11.4017	126.2000	118.3478	28.7341	− 0.2133	20.6685
Base case	283.40	289.2225	5.8225	126.20	121.5936	0.0000	0.0000	− 4.6063

### Case-3

**Figure 21:**
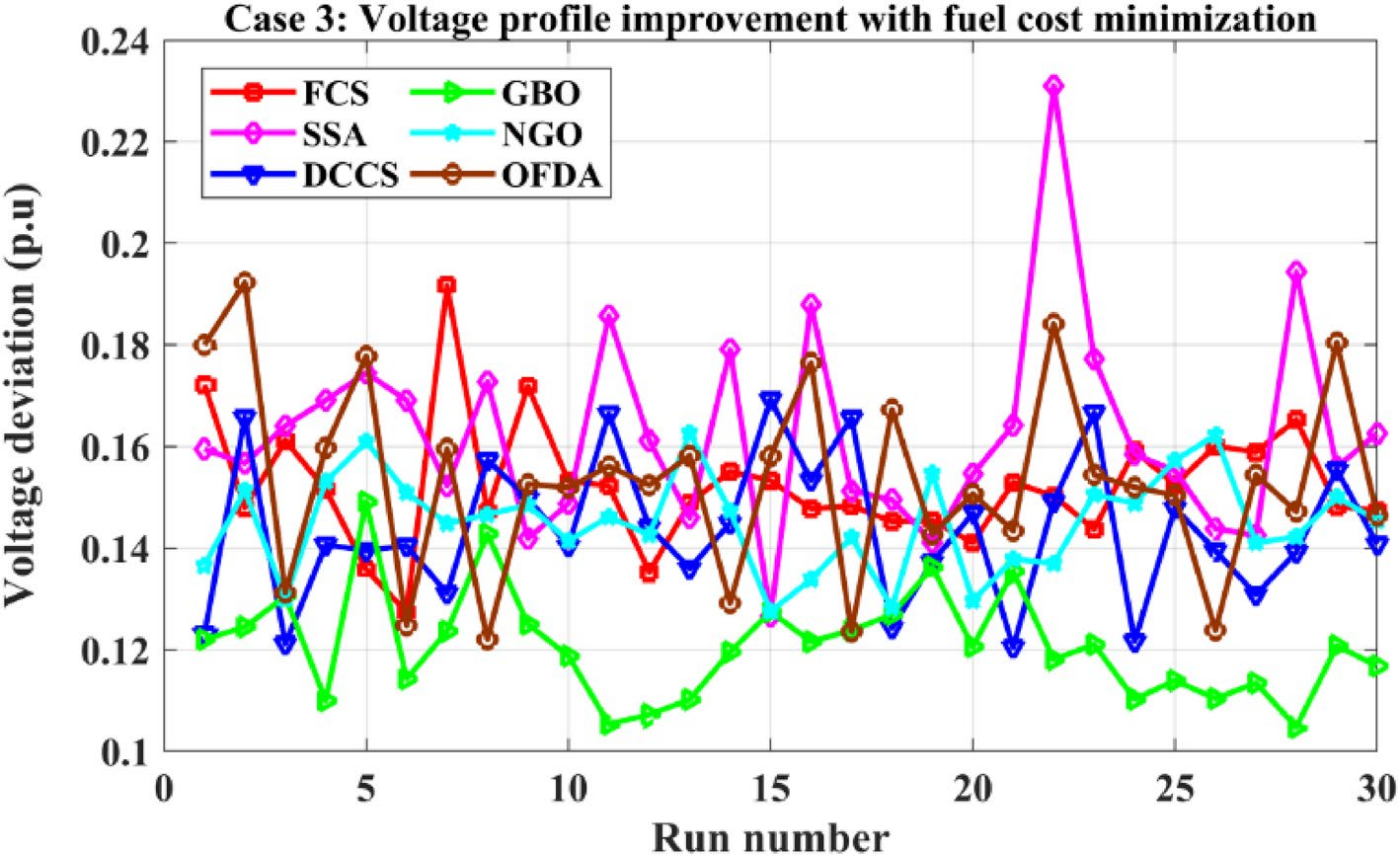
Variation of the objective function over the 30 runs for case 3.

**Figure 22:**
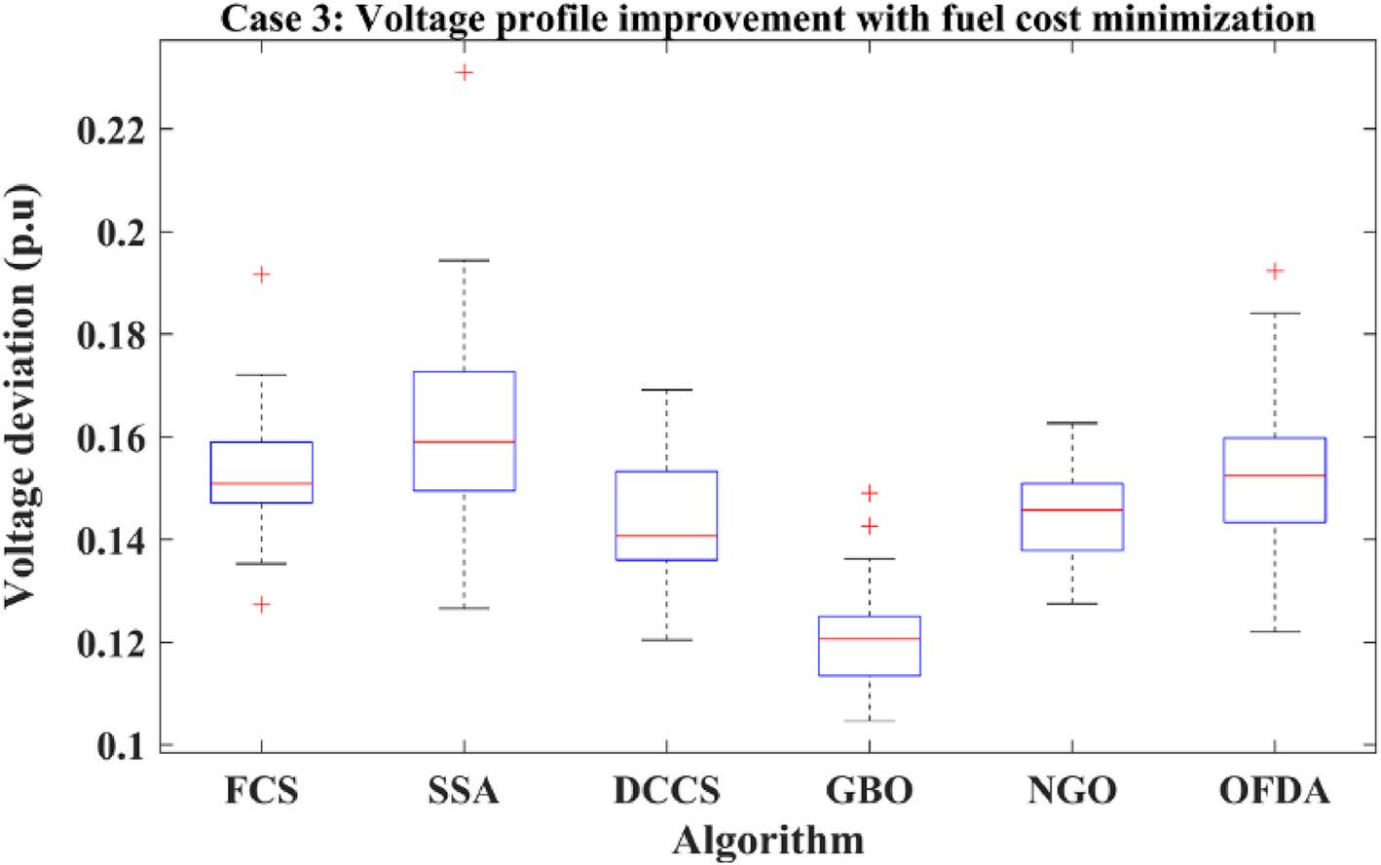
Boxplot for the results of the objective function of case 3.

**Figure 23:**
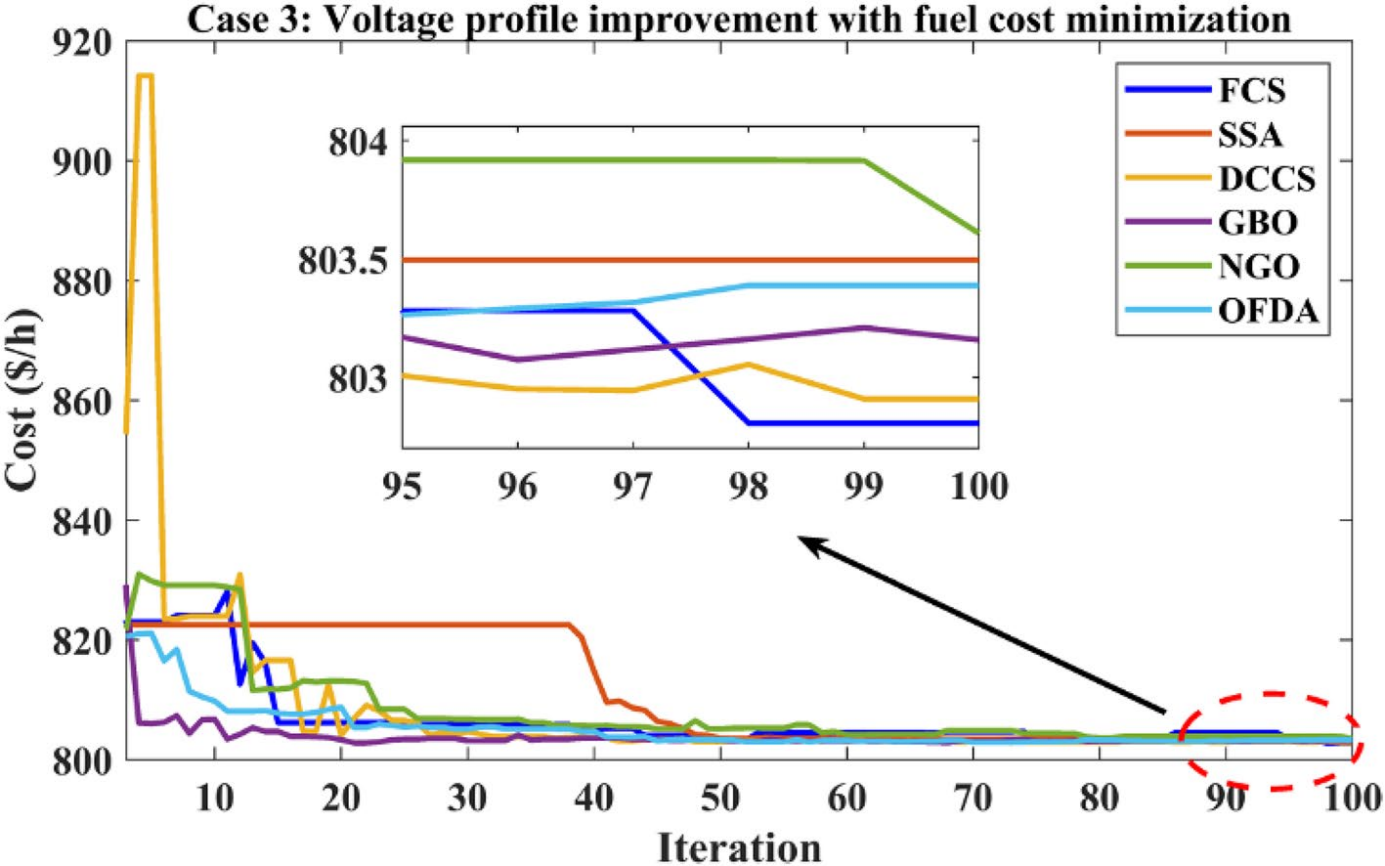
Variation of the total fuel cost of case 3.

**Figure 24:**
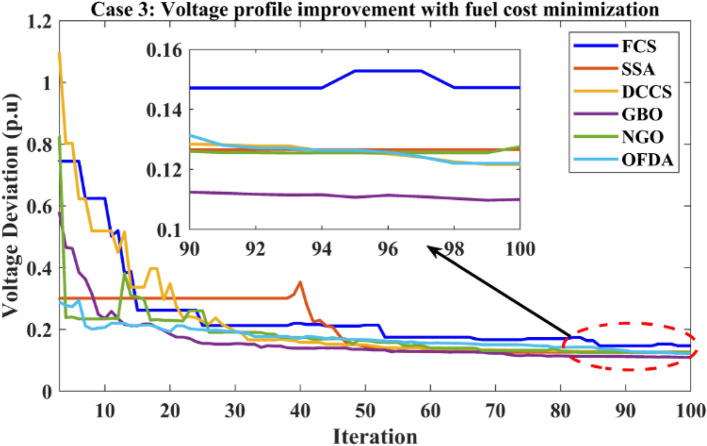
Variation of the total voltage deviation of case 3.

**Figure 25:**
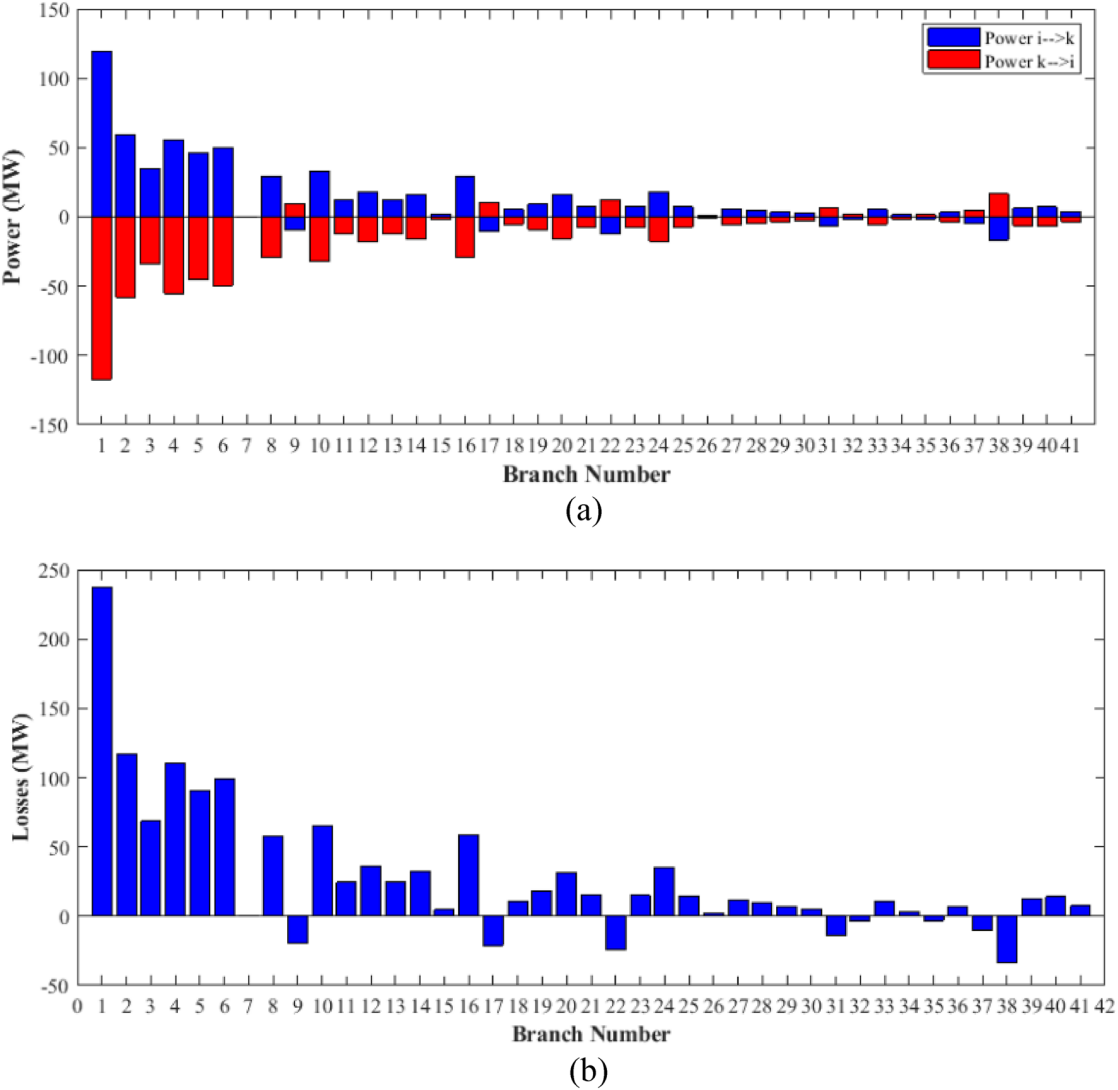
Active power flow and losses in the branches of the system for case 3.

**Figure 26:**
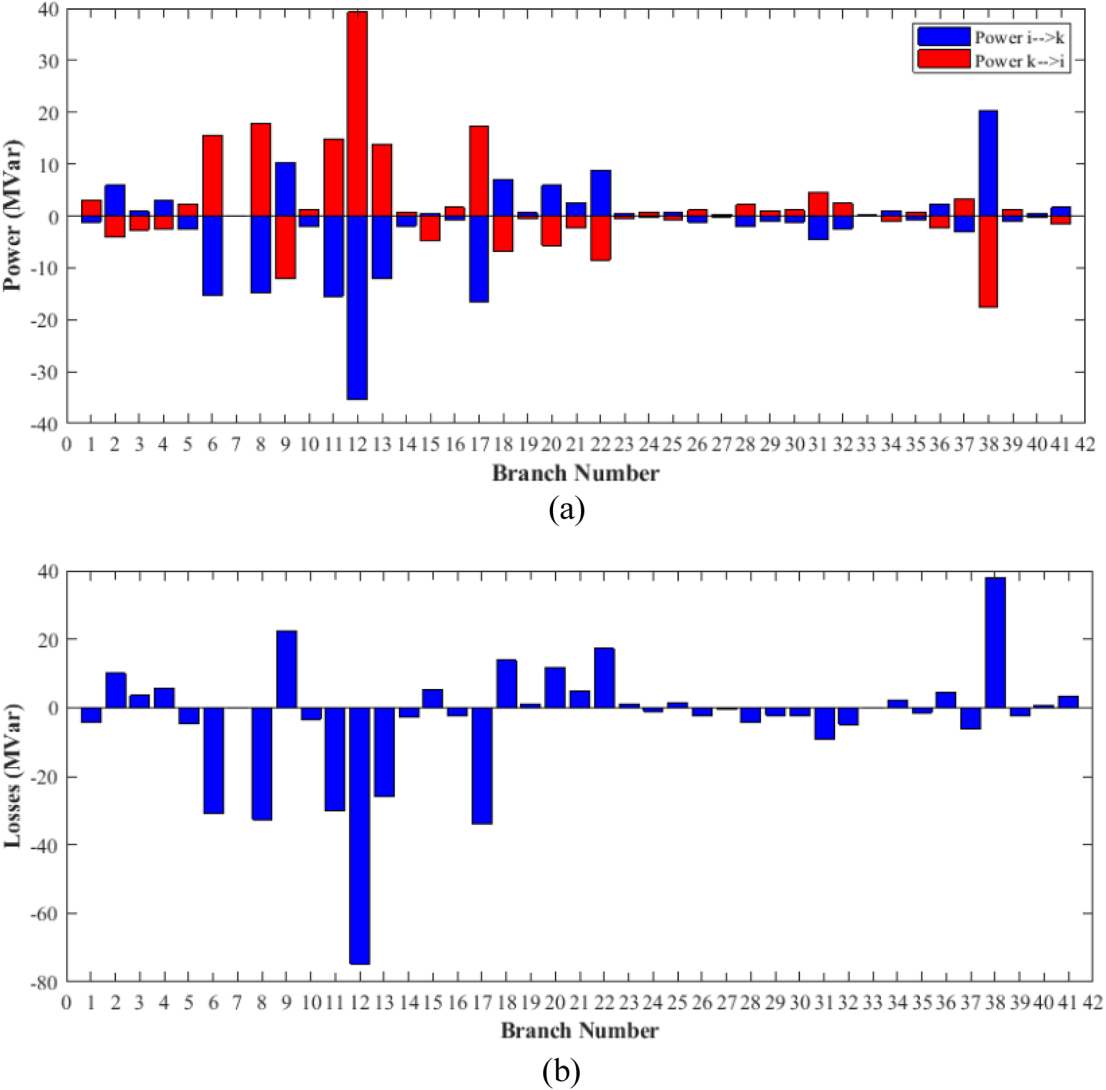
Reactive power flow and losses in the branches of the system for case 3.

**Figure 27:**
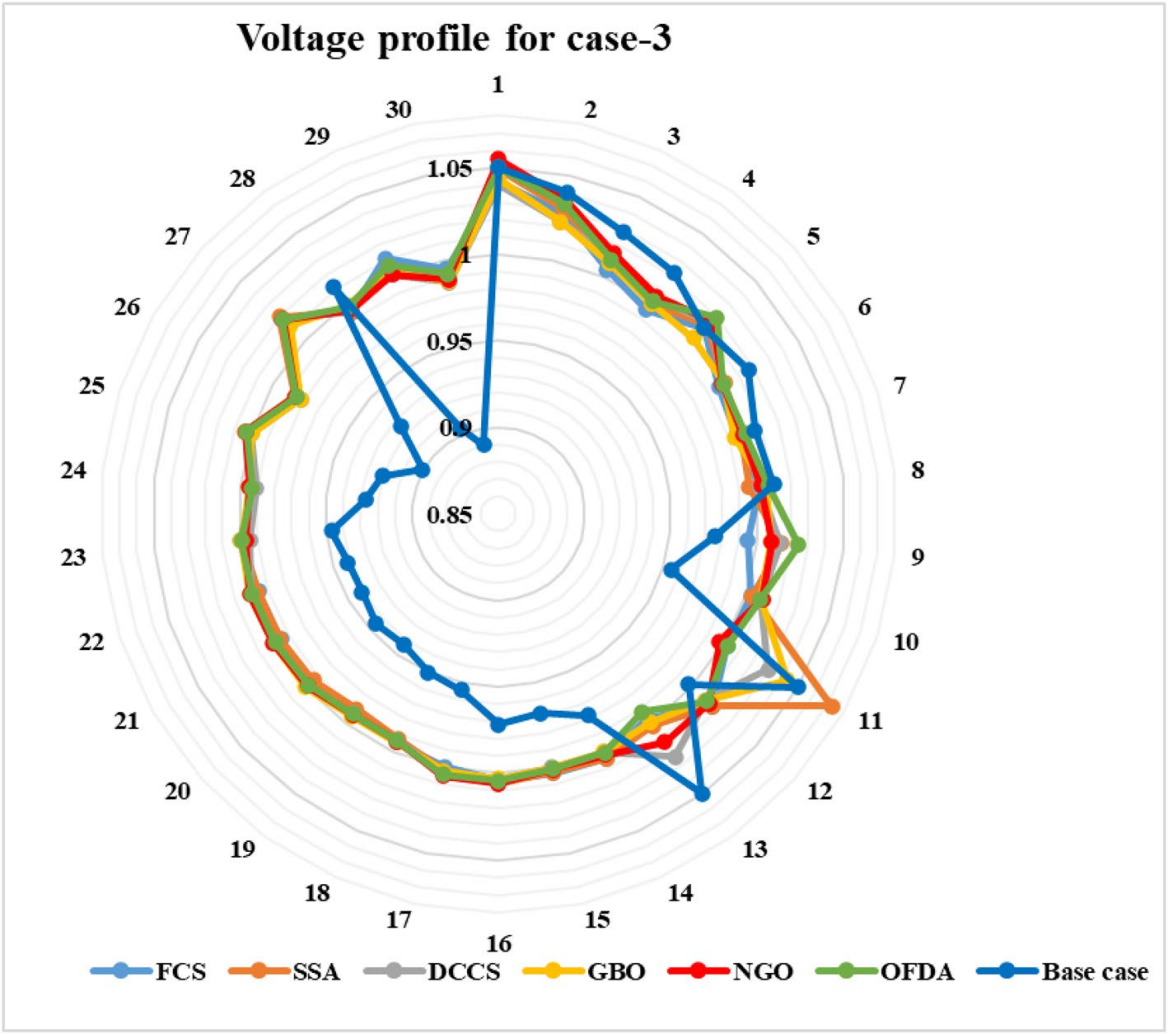
Voltage profile improvement for case 3.

The proposed algorithms have been implemented for 30 individual runs to address the optimization problem of OPF, incorporating the objective function of case 3. The obtained results of the best value of the voltage deviation in each run are recorded an presented in the graph shown in Fig. 21. Statistical study has been conducted and the results are listed in Table 8. A boxplot based on the 30 values of the total voltage deviation is sketched in Fig. 22. In this case, the GBO optimization technique provided the best performance compared with the others. The minimum value of the total voltage deviation while minimizing the total fuel cost obtained from GBO is 0.10474 p.u compared to 0.12739 p.u for FCS, 0.12657 p.u for SSA, 0.12045 p.u for DCCS, 0.12751 p.u for NGO, and 0.12203 p.u for OFDA. The variation of the best value of the objective function over the 100 iterations of the best runs for all algorithms is presented in Fig. 23 while the variation of the voltage deviation is provided in Fig. 24. The results of the optimization process for case 3 compared to the base case are provided in Table 9. The active power flow in the 41 branches is presented in Fig. 25a and the power losses in each branch is sketched in Fig. 25b. Similarly, the reactive power flow is presented in Fig. 26a and the reactive power losses in each branch is sketched in Fig. 26b. The impact of the optimization process on the voltage profile of the PQ buses of the system is presented in Fig. 27. Finally the active and reactive power balance based on the results of the six proposed algorithms is provided in Table 10.

**Table 8:** Statistical study for case 3.

	Min.	Max.	Mean	SD	RMSE
FCS	0.12739	0.19165	0.15236	1.22381	0.02772
SSA	0.12657	0.23102	0.16254	2.01004	0.04104
DCCS	0.12045	0.16919	0.14362	1.44017	0.02716
GBO	0.10474	0.14910	0.12080	1.06211	0.01915
NGO	0.12752	0.16262	0.14507	0.97341	0.01999
OFDA	0.12203	0.19228	0.15343	1.88407	0.03646

**Table 9:** Optimization results for case 3.

	0	Min	Max	FCS	SSA	DCCS	GBO	NGO	OFDA
P1	99.2230	50	200	171.483	175.698	176.605	179.119	175.450	178.79
P2	80	20	80	55.9090	47.9608	47.5101	48.4494	46.7762	45.156
P5	50	15	50	20.0307	20.5598	20.3638	23.0386	22.9869	22.522
P8	20	10	35	17.3454	14.1852	22.8659	20.0901	19.9036	15.091
P11	20	10	30	13.6272	18.5351	13.7879	10.4416	15.6350	18.779
P13	20	12	40	14.8893	16.1998	12.0233	12.1414	12.1670	12.954
V1	1.05	0.95	1.1	1.0413	1.0486	1.0403	1.0434	1.0549	1.0499
V2	1.04	0.95	1.1	1.0275	1.0304	1.0223	1.0225	1.0359	1.0333
V5	1.01	0.95	1.1	1.0103	1.0120	1.0143	1.0018	1.0165	1.0196
V8	1.01	0.95	1.1	1.0014	0.9957	1.0018	1.0054	1.0025	1.0065
V11	1.05	0.95	1.1	1.0025	1.0723	1.0299	1.0426	0.9977	1.0029
V13	1.05	0.95	1.1	0.9958	1.0015	1.0236	0.9991	1.0133	0.9914
T11	1.078	0.9	1.1	1.0256	1.0221	0.9923	1.0275	0.9818	0.9292
T12	1.069	0.9	1.1	0.9037	0.9997	0.9333	0.9129	0.9017	0.9866
T15	1.032	0.9	1.1	0.9205	0.9311	0.9918	0.9485	0.9611	0.9407
T36	1.068	0.9	1.1	0.9599	0.9443	0.9594	0.9743	0.9528	0.9601
QC10	0	0	5	1.5799	1.8378	3.3097	4.4807	2.1883	3.7426
QC12	0	0	5	2.3572	1.1675	1.1658	1.5634	0.4280	4.1159
QC15	0	0	5	2.5782	3.2175	4.5819	2.0494	2.6327	3.0353
QC17	0	0	5	2.0938	4.8324	0.9510	0.0000	2.1443	2.7324
QC20	0	0	5	4.9488	2.5396	3.9085	4.8233	3.1462	3.4091
QC21	0	0	5	4.1980	4.4215	4.7850	3.0436	2.0596	1.6588
QC23	0	0	5	4.5061	3.5784	1.7364	4.9046	1.7978	4.7188
QC24	0	0	5	3.8301	3.2977	2.4056	4.9966	4.9605	3.1955
QC27	0	0	5	4.3161	0.8129	2.2228	3.8675	1.1975	3.1485
Fuel cost ($/h)	**901.951**	**–**	**–**	**802.806**	**803.496**	**802.908**	**803.158**	**803.608**	**803.38**
Active power losses (MW)	5.8219	**–**	**–**	9.5239	9.9269	9.4940	9.4560	9.7492	9.5590
Reactive power losses (MVar)	− 4.6066	**–**	**–**	11.0444	10.9820	9.0626	10.1909	10.4334	9.6266
Voltage deviation	**1.1496**	**–**	**–**	**0.1373**	**0.1375**	**0.1377**	**0.1372**	**0.1386**	**0.1372**
Lmax	0.17233	**–**	**–**	0.1472	0.1266	0.1216	0.1100	0.1275	0.1220

Significant values are in bold.

**Table 10:** Active and reactive power balance for case 3.

Case 3	Active power balance			Reactive power balance				
Method	Load (MW)	Generation (MW)	Loss (MW)	Load (MVar)	Generation (MVar)	Compensation (MVar)	Charging (MVar)	Loss (MVar)
FCS	283.40	293.2855	9.9713	126.20	105.0151	30.4083	2.3215	11.5448
SSA	283.40	293.1394	9.8321	126.20	108.7209	25.7054	5.0744	13.3008
DCCS	283.40	293.1560	9.8538	126.20	107.1749	25.0668	5.9857	12.0274
GBO	283.40	293.2802	9.9761	126.20	105.2894	29.7292	3.4932	12.3117
NGO	283.40	292.9192	9.6126	126.20	111.5519	20.5550	4.2628	10.1697
OFDA	283.40	293.3013	9.9986	126.20	104.4034	29.7569	5.4469	13.4073
Base case	283.40	289.2225	5.8225	126.20	121.5936	0.0000	0.0000	− 4.6063

### Case-4

**Figure 28:**
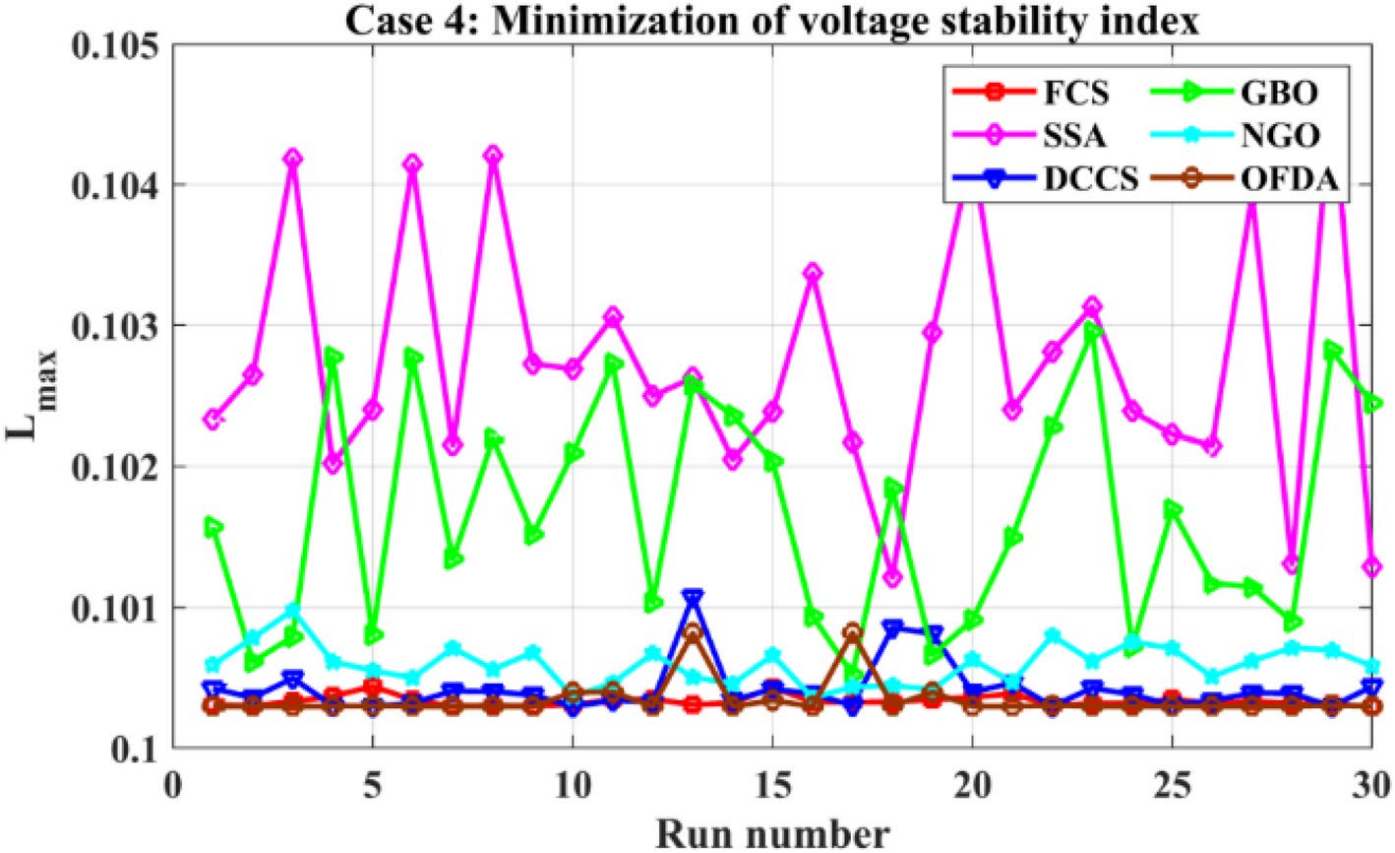
Variation of the L_max_ over the 30 runs for case 4.

**Figure 29:**
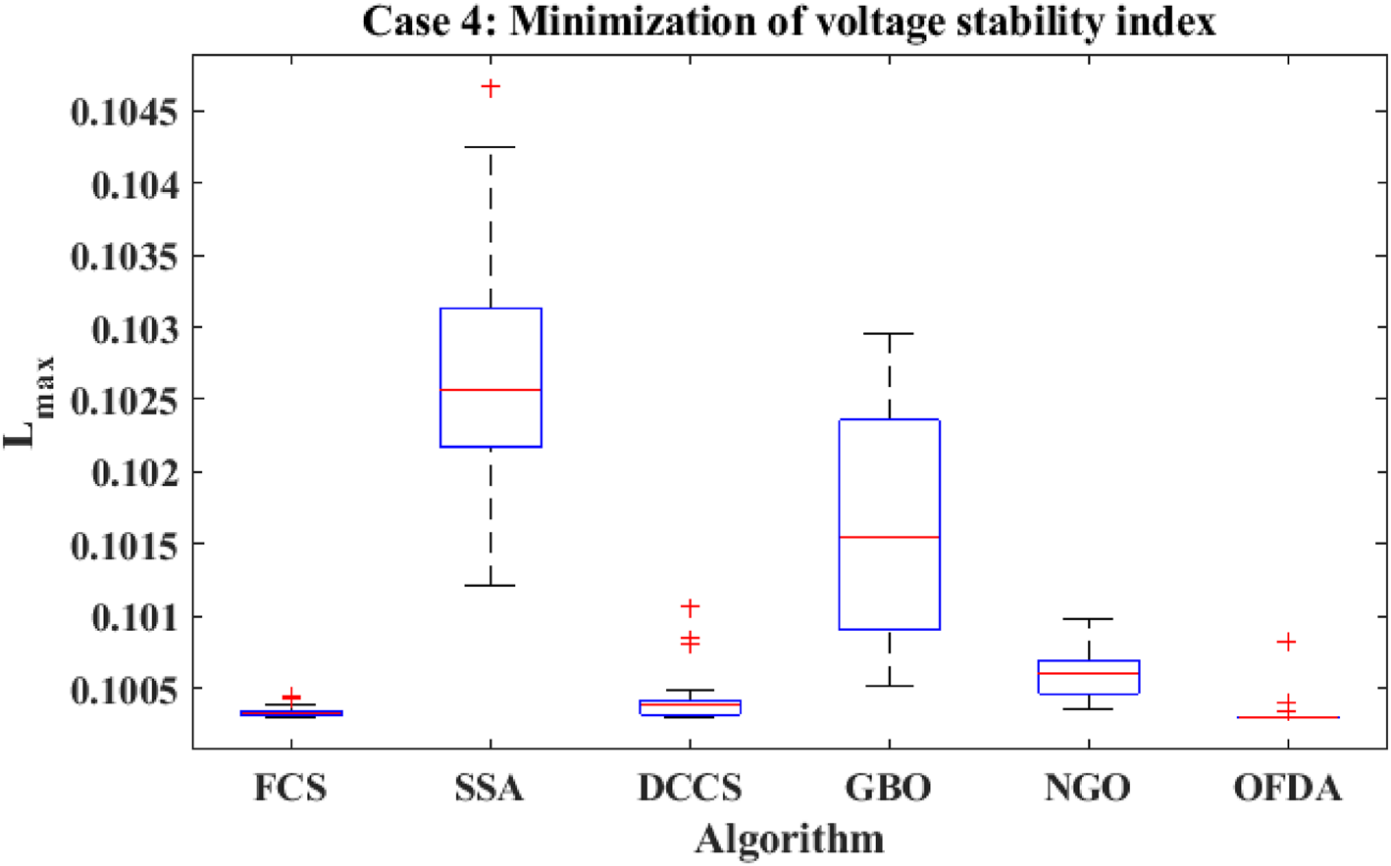
Boxplot for the results of the objective function of case 4.

**Figure 30:**
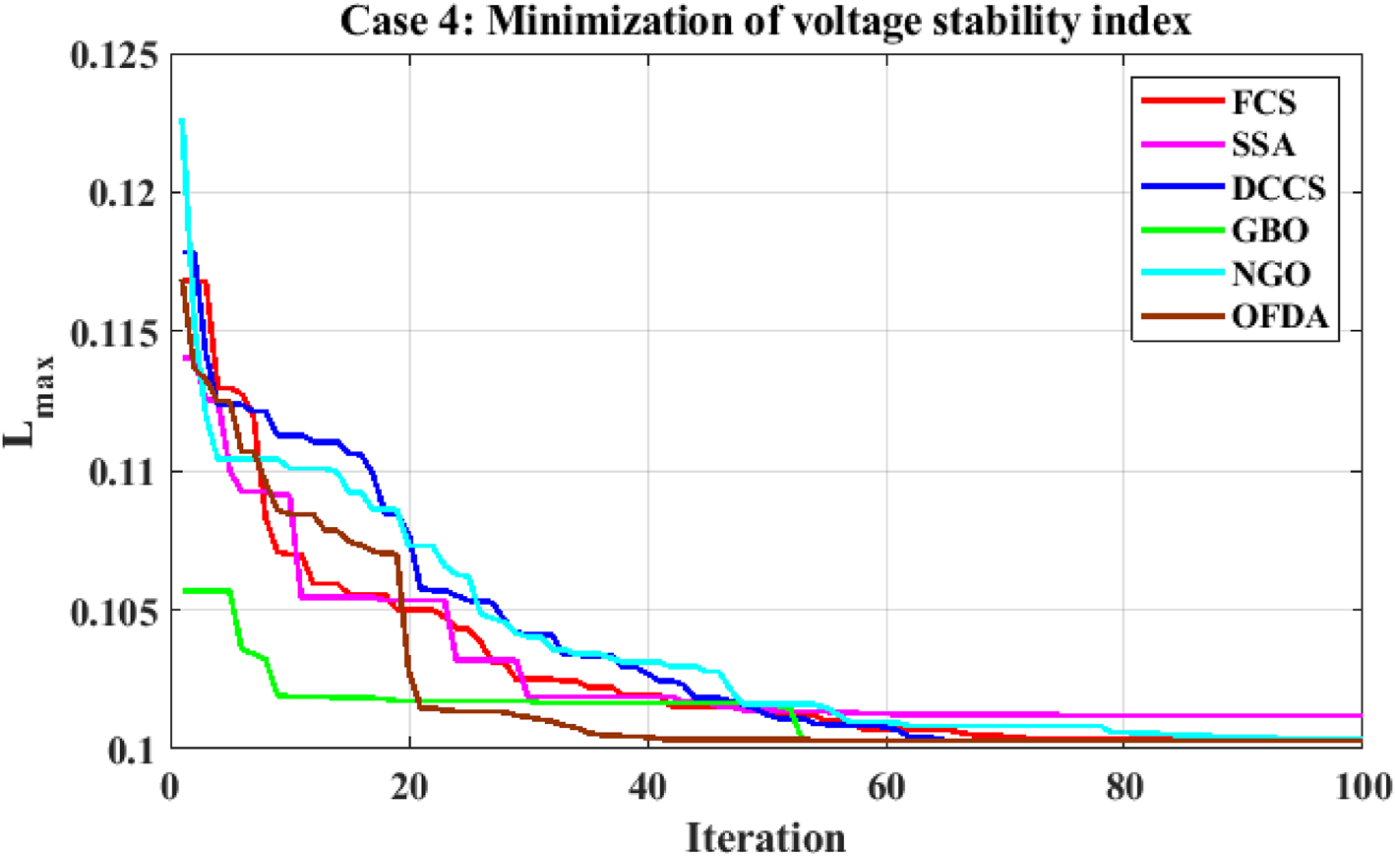
Variation of the L_max_ of case 4.

**Figure 31:**
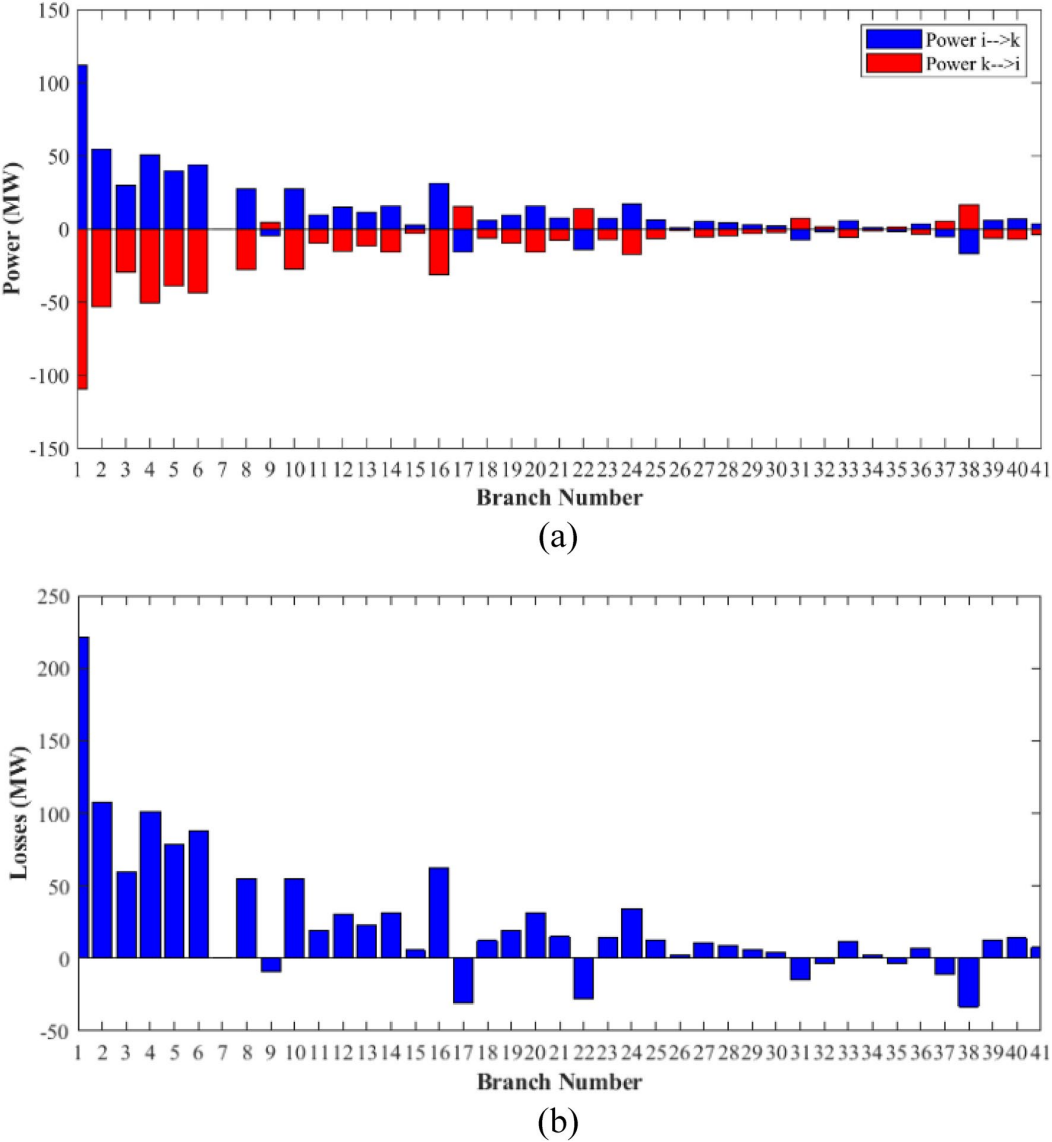
Active power flow and losses in the branches of the system for case 4.

**Figure 32:**
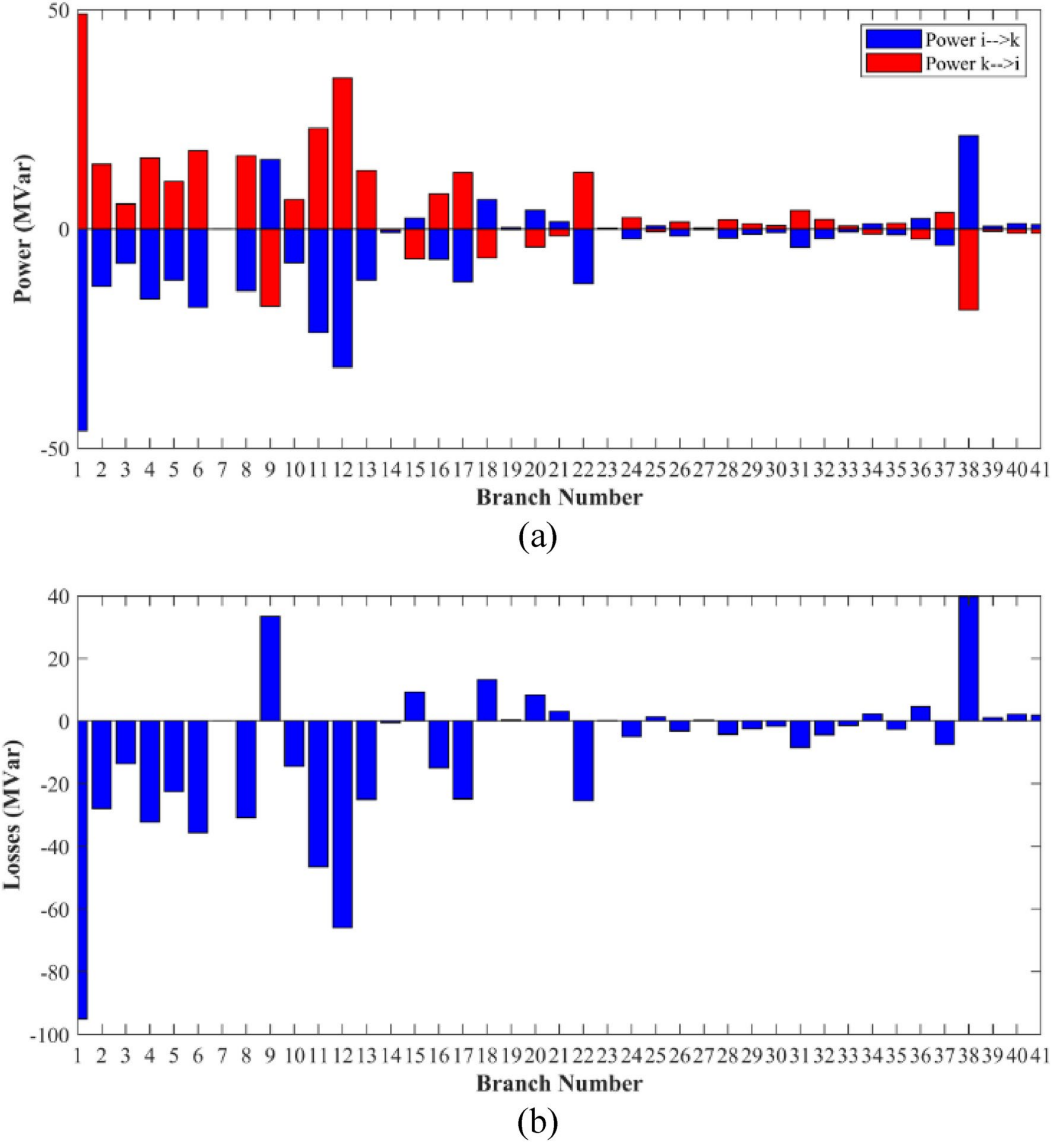
Reactive power flow and losses in the branches of the system for case 4.

**Figure 33:**
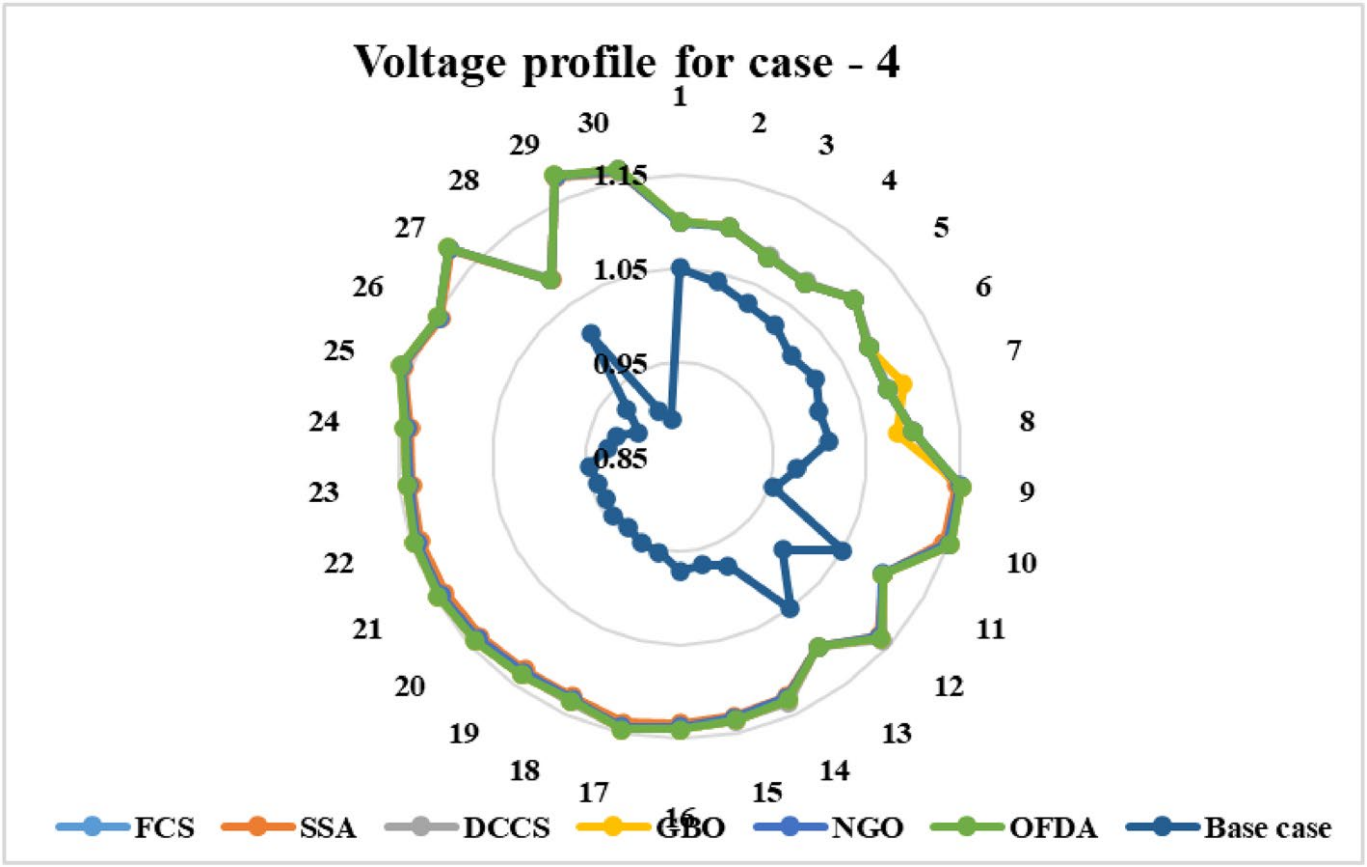
Voltage profile improvement for case 4.

The proposed algorithms have been implemented for 30 individual runs to address the optimization problem of OPF, incorporating the objective function of case 4. The obtained results of the best value of the voltage stability index L_max_ in each run is recorded an presented in the graph shown in Fig. 28. Statistical study has been conducted and the results are listed in Table 11. A boxplot based on the 30 values of the total voltage deviation is sketched in Fig. 29. Also, in this case, the GBO optimization technique provided sufficient performance compared with the others. The minimum value of the voltage stability index L_max_ obtained from GBO is 0.10052 compared to 0.1003 for FCS, 0.10121 for SSA, 0.1003 for DCCS, 0.10036 for NGO, and 0.1003 for OFDA. The variation of the voltage stability index L_max_ is provided in Fig. 30. The results of the optimization process for case 4 compared to the base case are provided in Table 12. The active power flow in the 41 branches is presented in Fig. 31a and the power losses in each branch is sketched in Fig. 31b. Similarly, the reactive power flow is presented in Fig. 32a and the reactive power losses in each branch is sketched in Fig. 32b. The impact of the optimization process on the voltage profile of the PQ buses of the system is presented in Fig. 33. Finally the active and reactive power balance based on the results of the six proposed algorithms is provided in Table 13.

**Table 11:** Statistical study foe case 4.

	Min.	Max.	Mean	SD	RMSE
FCS	0.10030	0.10044	0.10033	0.00354	0.00005
SSA	0.10121	0.10468	0.10275	0.09057	0.00177
DCCS	0.10030	0.10107	0.10042	0.01794	0.00022
GBO	0.10052	0.10295	0.10166	0.07888	0.00137
NGO	0.10036	0.10098	0.10059	0.01429	0.00028
OFDA	0.10030	0.10082	0.10034	0.01327	0.00014

**Table 12:** Optimization results for case 4.

	0	Min	Max	FCS	SSA	DCCS	GBO	NGO	OFDA
P1	99.2230	50	200	80.5485	135.257	149.869	80.5484	102.062	80.5485
P2	80	20	80	80.0000	60.0812	20.7991	80	71.5945	80.0000
P5	50	15	50	50.0000	42.0421	15.0002	50	50.0000	50.0000
P8	20	10	35	35.0000	16.7646	35.0000	35	30.0827	35.0000
P11	20	10	30	30.0000	22.5182	30.0000	30	19.6960	30.0000
P13	20	12	40	12.0000	13.3376	39.9962	12	14.9493	12.0000
V1	1.05	0.95	1.1	1.1000	1.0997	1.1000	1.1	1.0983	1.1000
V2	1.04	0.95	1.1	1.1000	1.1000	1.1000	1.1	1.0998	1.1000
V5	1.01	0.95	1.1	1.1000	1.1000	1.1000	1.1	1.1000	1.1000
V8	1.01	0.95	1.1	1.1000	1.1000	1.1000	1.1	1.1000	1.1000
V11	1.05	0.95	1.1	1.1000	1.1000	1.1000	1.1	1.0984	1.1000
V13	1.05	0.95	1.1	1.1000	1.1000	1.1000	1.1	1.1000	1.1000
T11	1.078	0.9	1.1	0.9000	0.9062	0.9000	0.9	0.9001	0.9000
T12	1.069	0.9	1.1	0.9000	0.9034	0.9000	0.9	0.9000	0.9000
T15	1.032	0.9	1.1	0.9000	0.9036	0.9000	0.9	0.9073	0.9000
T36	1.068	0.9	1.1	0.9000	0.9000	0.9000	0.9	0.9000	0.9000
QC10	0	0	5	5.0000	4.2015	4.9999	5	4.7365	5.0000
QC12	0	0	5	5.0000	3.9634	5.0000	5	4.8599	5.0000
QC15	0	0	5	5.0000	4.9744	5.0000	5	5.0000	5.0000
QC17	0	0	5	5.0000	3.1136	5.0000	5	5.0000	5.0000
QC20	0	0	5	5.0000	4.9572	5.0000	5	4.9268	5.0000
QC21	0	0	5	5.0000	4.3722	5.0000	5	4.8441	5.0000
QC23	0	0	5	5.0000	4.9862	5.0000	5	5.0000	5.0000
QC24	0	0	5	5.0000	4.7164	5.0000	5	4.9727	5.0000
QC27	0	0	5	5.0000	4.9998	5.0000	5	4.9840	5.0000
Fuel cost ($/h)	901.9516	–	–	919.743	841.465	853.446	919.743	888.967	919.743
Active power losses (MW)	5.8219	–	–	4.1518	6.6041	7.2685	4.1518	4.9885	4.1518
Reactive power losses (MVar)	− 4.6066	–	–	6.0257	11.8872	15.5802	6.0257	7.4156	6.0257
Voltage deviation	1.1496	–	–	3.2307	3.1290	3.2291	3.2307	3.1847	3.2307
Lmax	**0.17233**	–	–	**0.1003**	**0.1013**	**0.1004**	**0.1003**	**0.1006**	**0.1003**

Significant values are in bold.

**Table 13:** Active and reactive power balance for case 4.

Case-4	Active power balance			Reactive power balance				
Method	load (MW)	Generation (MW)	Loss (MW)	Load (MVar)	Generation (MVar)	Compensation (MVar)	Charging (MVar)	Loss (MVar)
FCS	283.40	287.5485	4.1518	126.20	66.1857	45.0000	21.0399	6.0257
SSA	283.40	290.0010	6.6041	126.20	78.1066	40.2848	19.6959	11.8872
DCCS	283.40	290.6649	7.2685	126.20	76.1957	44.9999	20.5845	15.5802
GBO	283.40	287.5485	4.1518	126.20	66.1857	45.0000	21.0399	6.0257
NGO	283.40	288.3853	4.9885	126.20	68.6511	44.3239	20.6406	7.4156
OFDA	283.40	287.5485	4.1518	126.20	66.1857	45.0000	21.0399	6.0257
Base case	283.40	289.2225	5.8225	126.20	121.5936	0.0000	0.0000	− 4.6063

### Case-5

**Figure 34:**
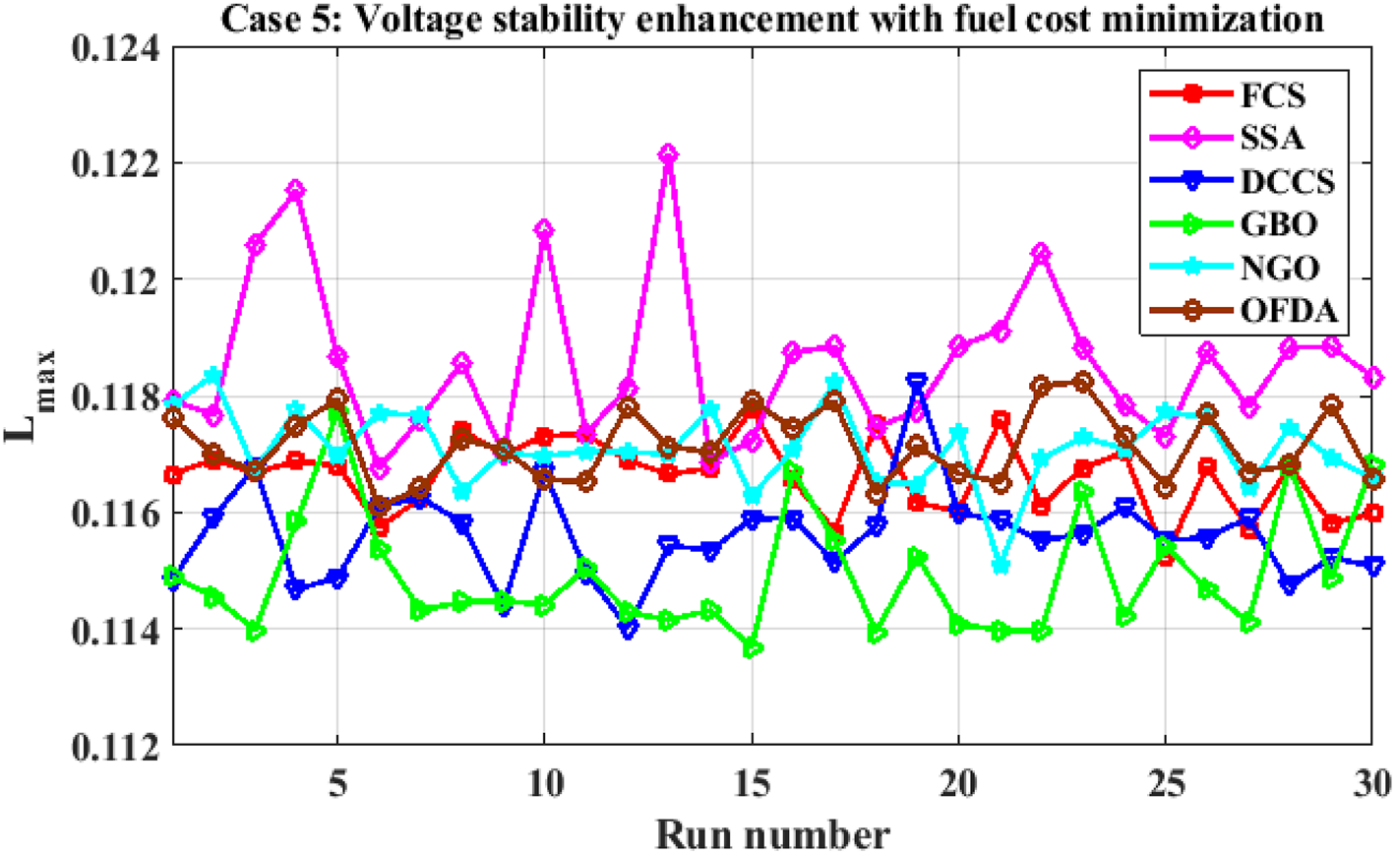
Variation of the L_max_ over the 30 runs for case 5.

**Figure 35:**
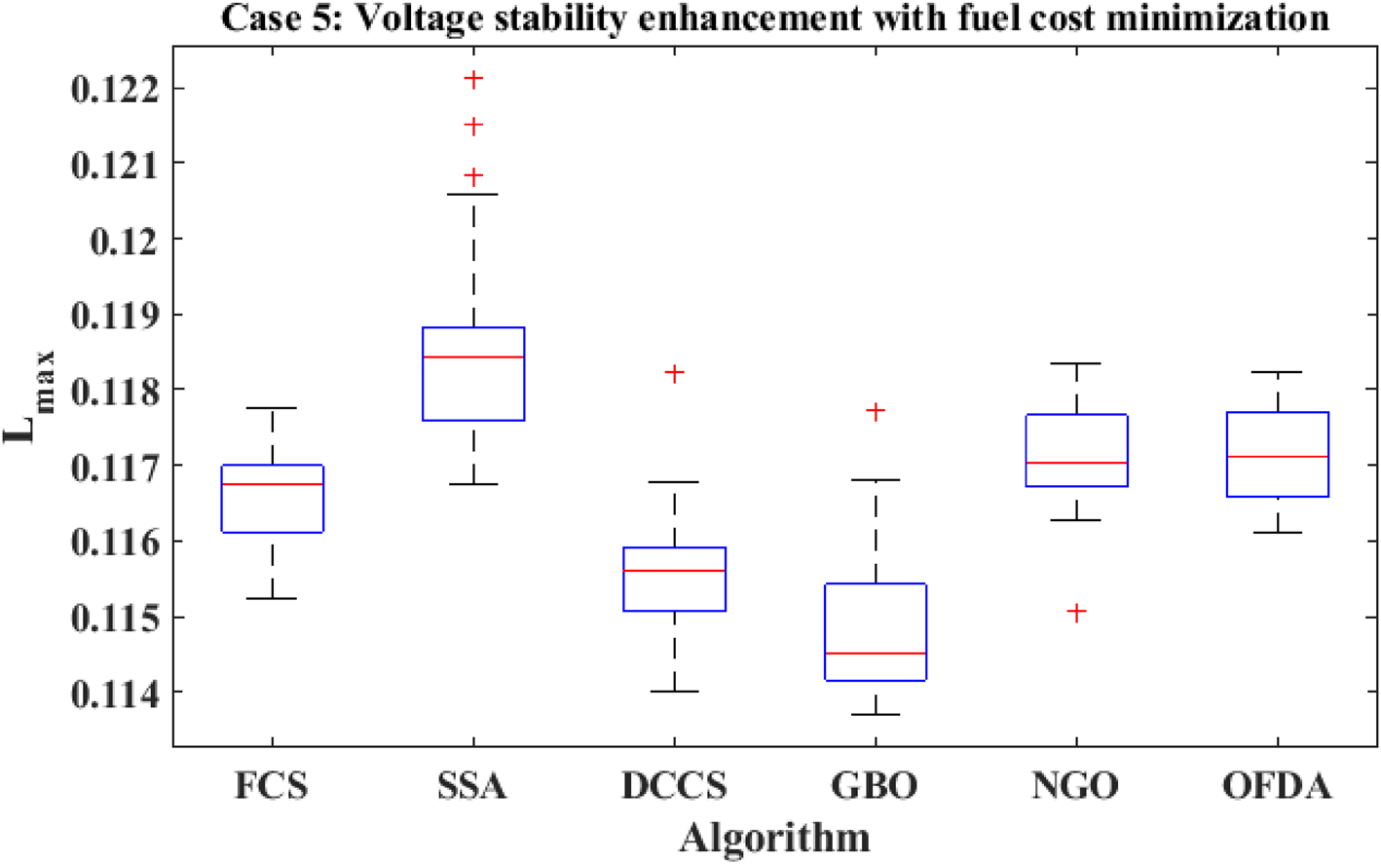
Boxplot for the results of the objective function of case 5.

**Figure 36:**
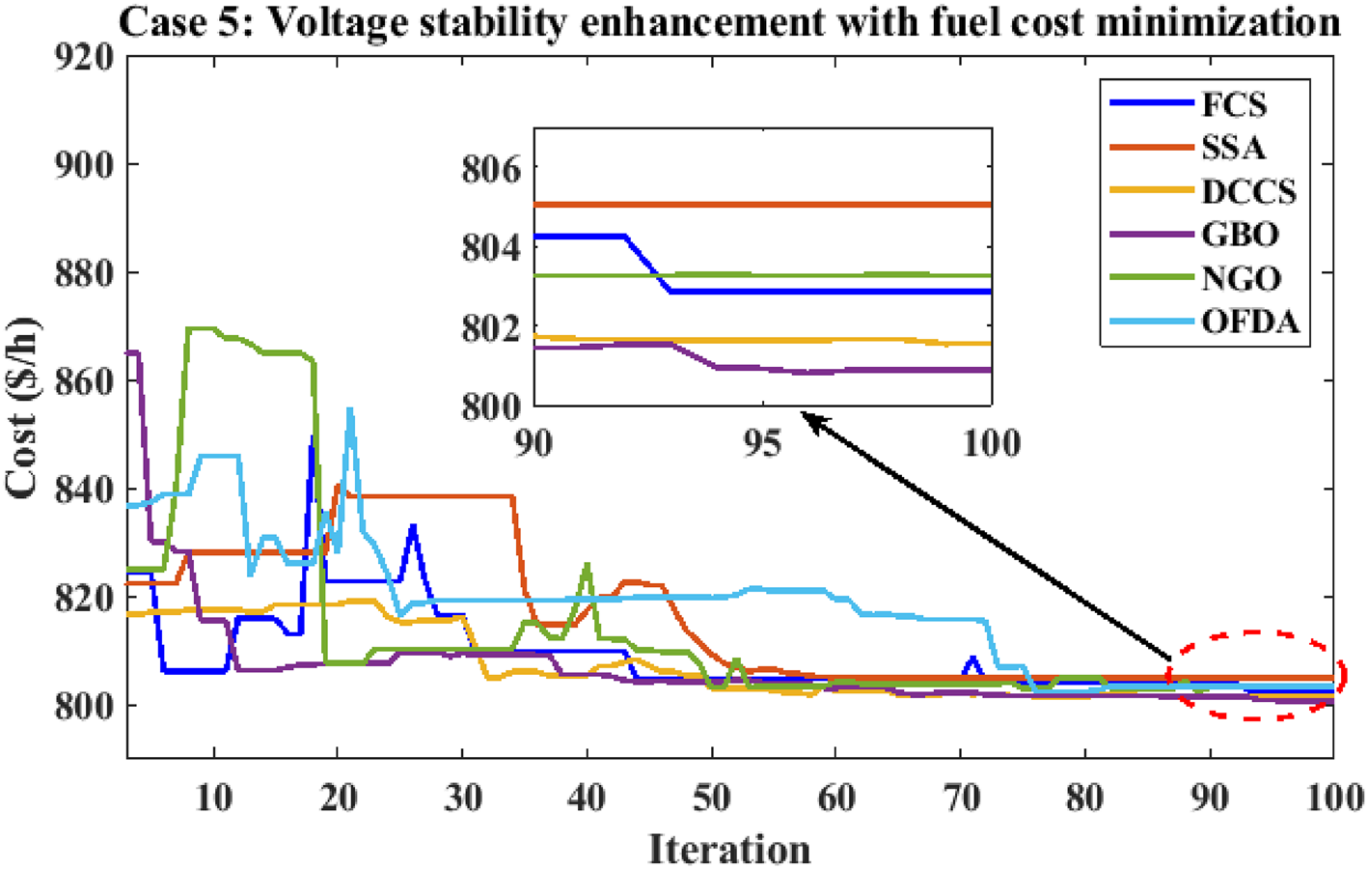
Variation of the total fuel generation cost of case 5.

**Figure 37:**
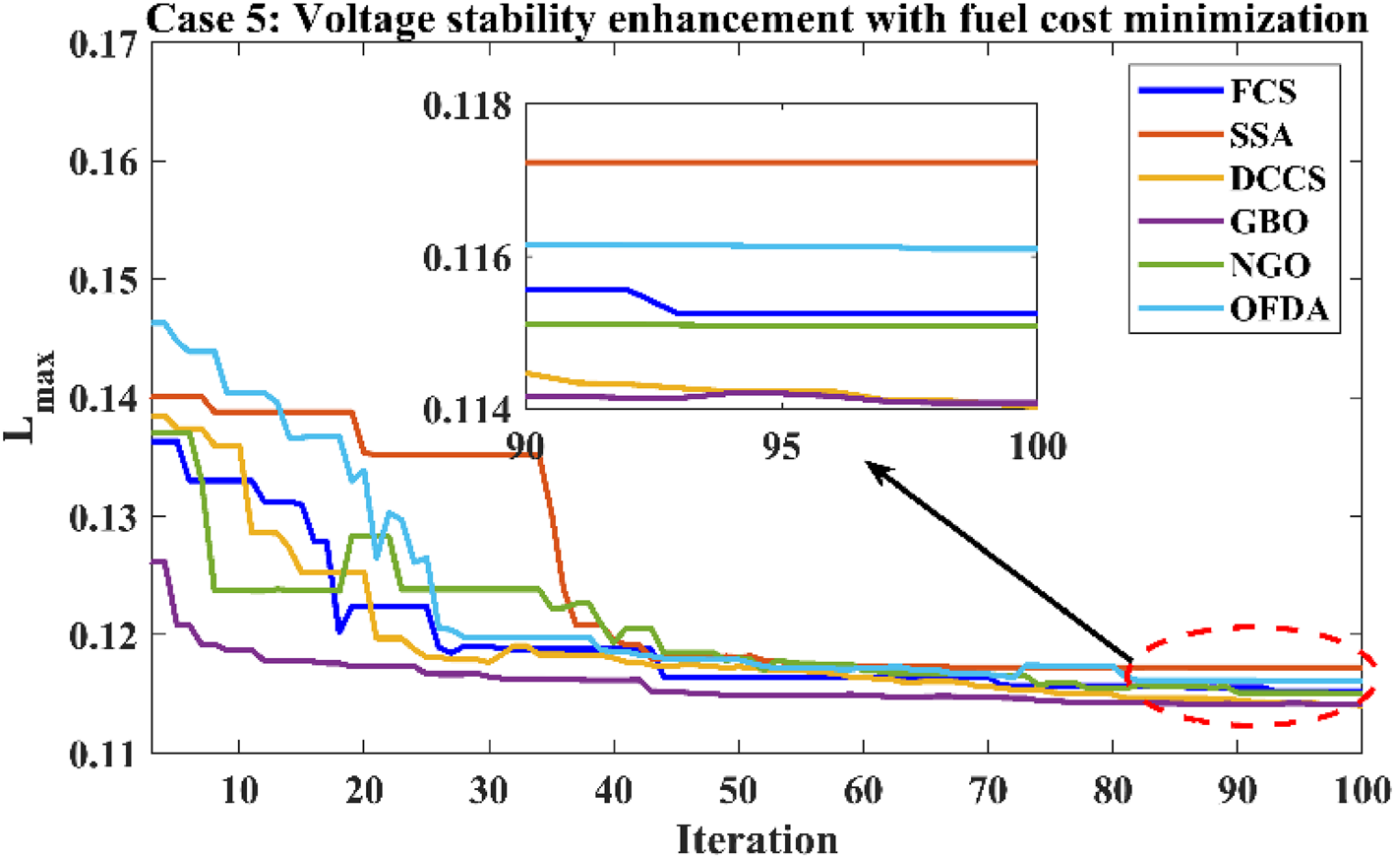
Variation of the L_max_ of case 5.

**Figure 38:**
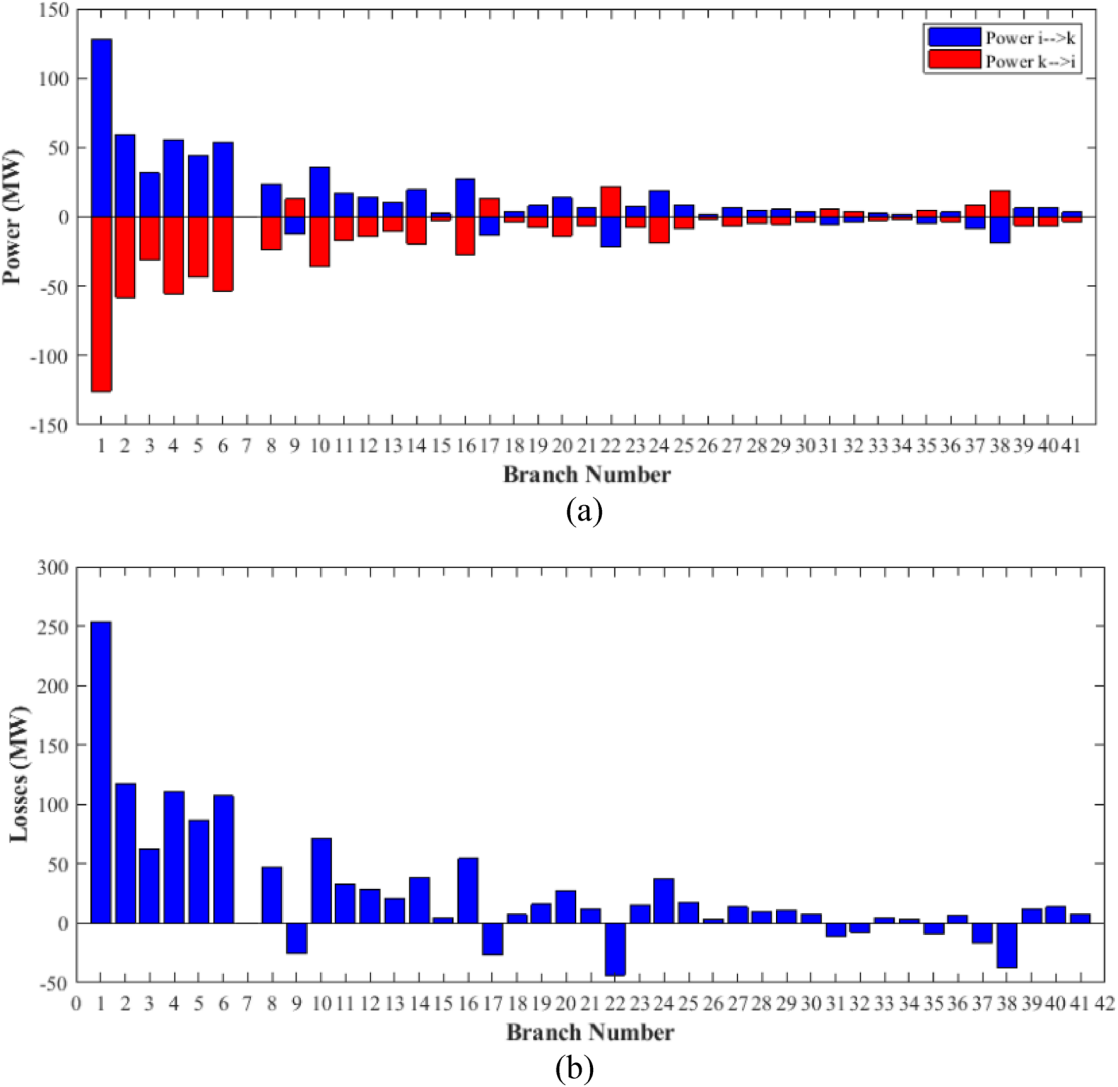
Active power flow and losses in the branches of the system for case 5.

**Figure 39:**
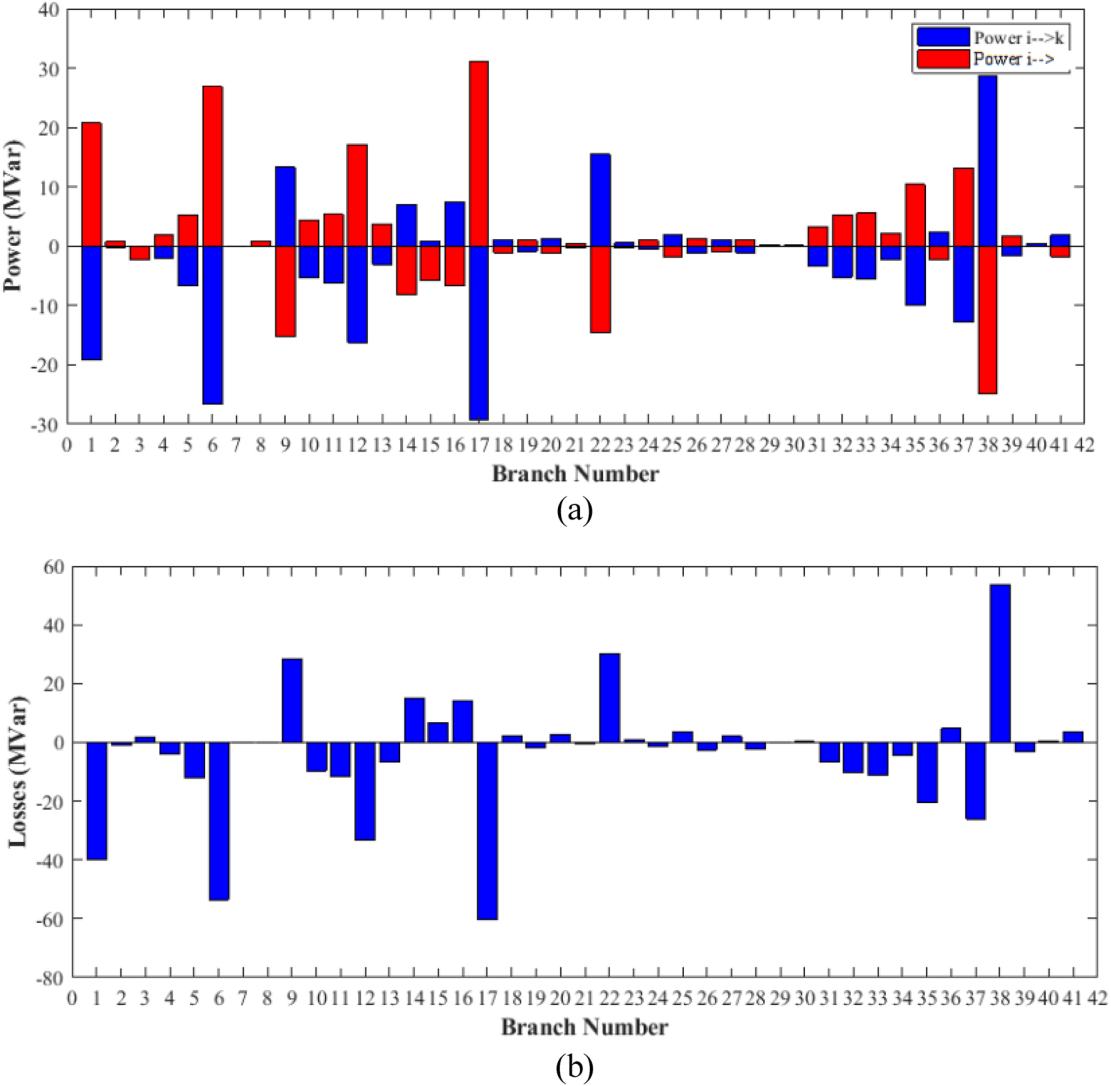
Reactive power flow and losses in the branches of the system for case 5.

**Figure 40:**
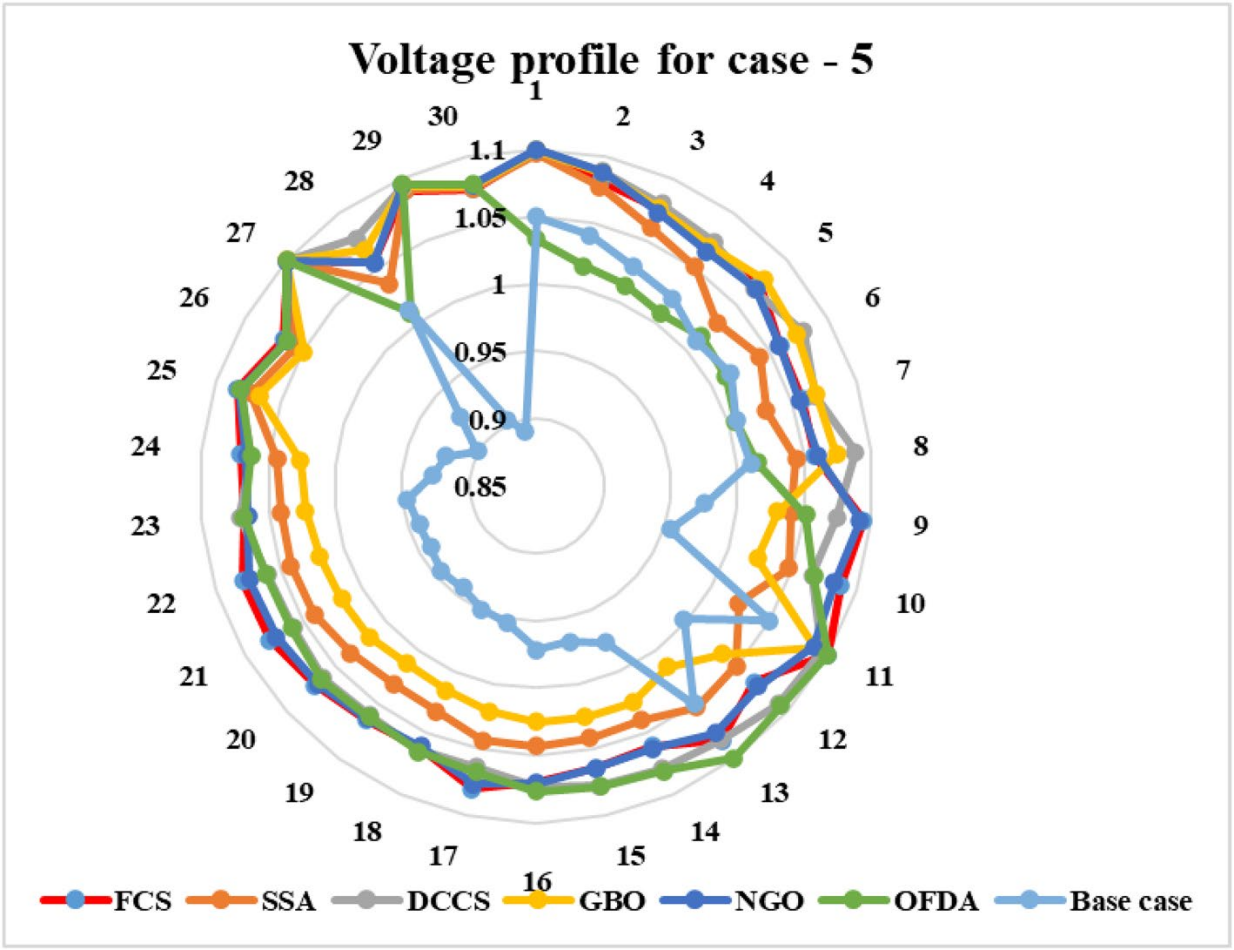
Voltage profile improvement for case 5.

The proposed algorithms have been implemented for 30 individual runs to address the optimization problem of OPF, incorporating the objective function of case 5. The obtained results of the best value of the voltage stability index in each run are recorded and presented in the graph shown in Fig. 34. Statistical study has been conducted and the results are listed in Table 14. A boxplot based on the 30 values of the voltage stability index L_max_ is sketched in Fig. 35. In this case, the GBO optimization technique provided the best performance compared to the others. The minimum value of the voltage stability index obtained from GBO is 0.11369 compared to 0.11524 for FCS, 0.11677 for SSA, 0.11402 for DCCS, 0.11508 for NGO, and 0.11610 for OFDA. The variation of the best value of the objective function over the 100 iterations of the best runs for all algorithms is presented in Fig. 36, while the variation of the voltage stability index L_max_ is provided in Fig. 37. The results of the optimization process for case 5 compared to the base case are provided in Table 15. The active power flow in the 41 branches is presented in Fig. 38a, and the power losses in each branch is sketched in Fig. 38b. Similarly, Fig. 39a presents the reactive power flow and the reactive power losses in each branch is sketched in Fig. 39b. The impact of the optimization process on the voltage profile of the system's PQ buses is presented in Fig. 40. Finally, the active and reactive power balance based on the results of the six proposed algorithms is provided in Table 16.

**Table 14:** Statistical study for case5.

	Min.	Max.	Mean	SD	RMSE
FCS	0.11524	0.11777	0.11663	0.06341	0.00152
SSA	0.11677	0.12214	0.11855	0.13633	0.00223
DCCS	0.11402	0.11822	0.11561	0.08069	0.00177
GBO	0.11369	0.11772	0.11494	0.10419	0.00162
NGO	0.11508	0.11835	0.11711	0.06625	0.00213
OFDA	0.11610	0.11824	0.11714	0.06091	0.00121

**Table 15:** Optimization results for case 5.

	0	Min	Max	FCS	SSA	DCCS	GBO	NGO	OFDA
P1	99.2230	50	200	161.767	146.648	169.293	187.564	186.450	172.864
P2	80	20	80	61.3519	48.4028	41.7717	37.1185	40.6365	51.2222
P5	50	15	50	27.1020	23.0335	27.4086	17.7762	21.5477	23.7671
P8	20	10	35	13.6537	29.3123	27.2724	15.8157	22.0451	18.1055
P11	20	10	30	10.0000	28.6487	10.0188	13.2071	10.0000	10.7688
P13	20	12	40	17.8884	14.5192	15.6692	21.7617	12.0000	16.4808
V1	1.05	0.95	1.1	1.0993	1.0967	1.1000	1.0993	1.1000	1.0329
V2	1.04	0.95	1.1	1.0816	1.0769	1.0890	1.0874	1.0884	1.0171
V5	1.01	0.95	1.1	1.0736	1.0308	1.0680	1.0785	1.0689	1.0155
V8	1.01	0.95	1.1	1.0582	1.0438	1.0884	1.0742	1.0603	1.0151
V11	1.05	0.95	1.1	1.1000	1.0237	1.0953	1.0896	1.0879	1.0998
V13	1.05	0.95	1.1	1.0838	1.0528	1.0830	1.0160	1.0766	1.1000
T11	1.078	0.9	1.1	0.9607	0.9947	1.0065	1.0881	0.9488	1.0375
T12	1.069	0.9	1.1	0.9192	0.9256	1.0663	1.0938	0.9355	0.9000
T15	1.032	0.9	1.1	1.0397	0.9923	0.9427	0.9776	0.9923	0.9000
T36	1.068	0.9	1.1	0.9428	0.9088	0.9601	0.9307	0.9448	0.9000
QC10	0	0	5	4.9663	0.6208	4.9669	4.5152	2.9579	4.9980
QC12	0	0	5	0.7346	2.9475	4.1023	2.3034	0.5289	3.0212
QC15	0	0	5	5.0000	4.6488	4.3828	4.7448	2.1836	4.9957
QC17	0	0	5	5.0000	4.3243	1.2959	4.8995	3.5535	4.4602
QC20	0	0	5	2.9350	2.3754	4.9284	4.9978	3.7882	5.0000
QC21	0	0	5	3.6721	4.7006	4.5125	4.7962	3.4416	4.9989
QC23	0	0	5	3.8852	2.1838	3.3142	0.5797	3.7950	0.0047
QC24	0	0	5	4.6851	4.9002	4.9834	4.4927	2.8157	5.0000
QC27	0	0	5	3.3958	3.2060	4.2244	4.4289	5.0000	4.9931
Fuel cost ($/h)	**901.951**	**–**	**–**	**802.844**	**805.060**	**801.550**	**800.869**	**803.265**	**803.476**
Active power losses (MW)	5.8219	**–**	**–**	8.5643	8.5777	8.1285	8.8882	7.9927	8.6669
Reactive power losses (MVar)	− 4.6066	**–**	**–**	7.4616	8.0580	4.8892	7.0128	0.1467	7.2879
Voltage deviation	1.1496	**–**	**–**	1.6699	1.5657	1.9690	1.8262	1.6888	1.6784
Lmax	**0.17233**	**–**	**–**	**0.1152**	**0.1172**	**0.1140**	**0.1136**	**0.1151**	**0.1161**

Significant values are in bold.

**Table 16:** Active and reactive power balance for case 5.

Case-5	Active power balance			Reactive power balance				
Method	Load (MW)	Generation (MW)	Losses (MW)	Load (MVar)	Generation (MVar)	Compensation (MVar)	Charging (MVar)	Losses (MVar)
FCS	283.40	291.7639	8.3646	126.20	88.6201	34.2742	9.3794	6.0736
SSA	283.40	290.5654	7.1657	126.20	90.8205	29.9073	6.3715	0.8993
DCCS	283.40	291.4337	8.0348	126.20	84.5002	36.7108	4.3557	− 0.6333
GBO	283.40	293.2435	9.8443	126.20	94.4310	35.7581	− 0.0653	3.9239
NGO	283.40	292.6801	9.2807	126.20	97.3998	28.0644	10.0211	9.2853
OFDA	283.40	293.2092	9.8094	126.20	97.7651	37.4718	15.7951	24.8320
Base case	283.40	289.2225	5.8225	126.20	121.5936	0.0000	0.0000	− 4.6063

### Case-6

**Figure 41:**
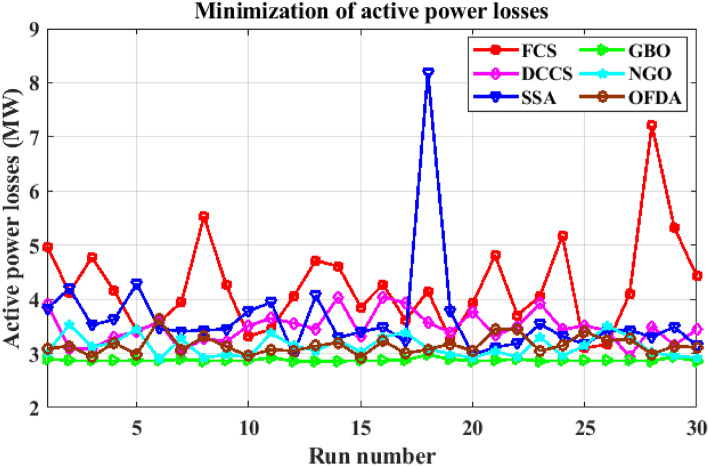
Variation of the objective function (Ploss) over the 30 runs for case 6.

**Figure 42:**
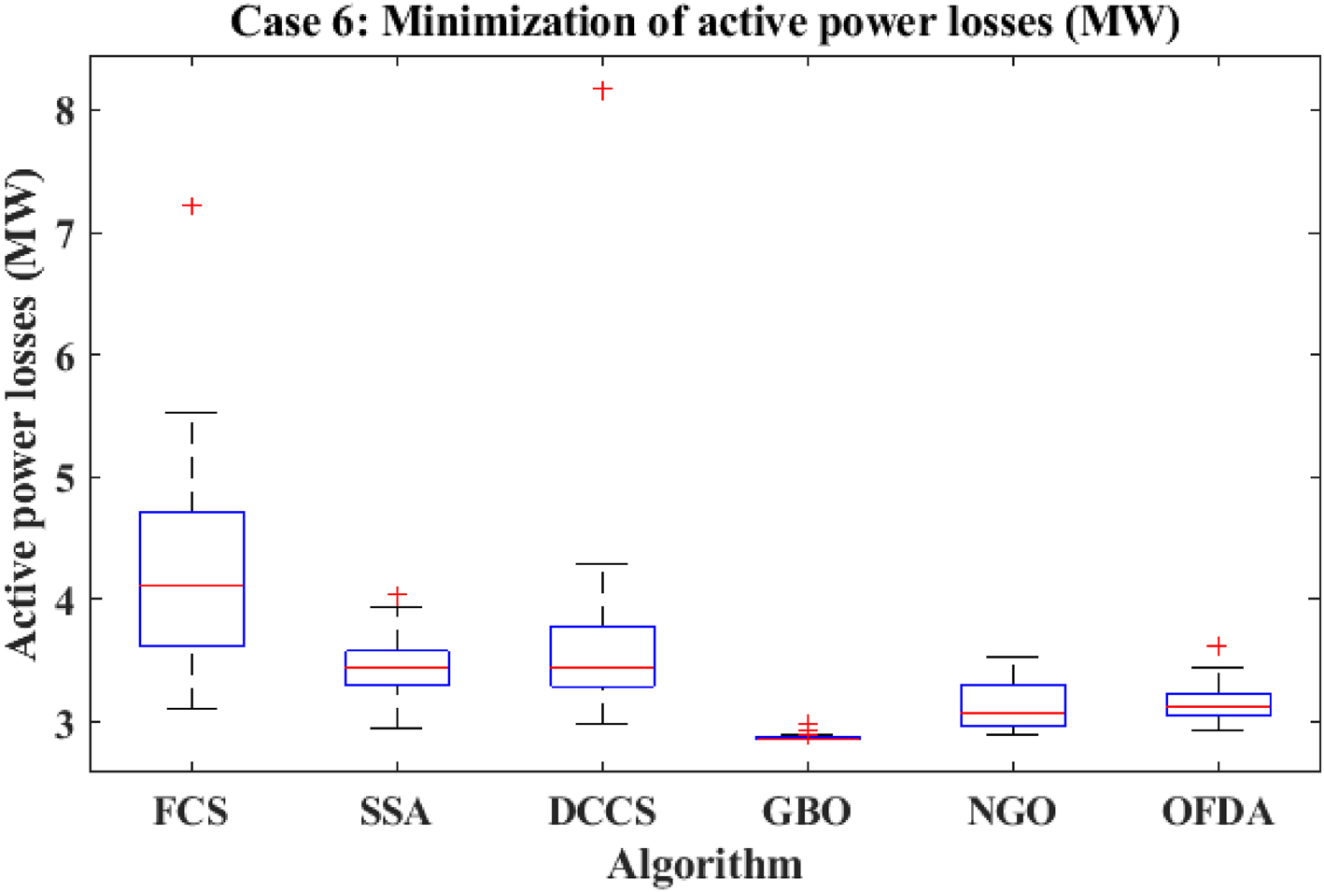
Boxplot for the results of the objective function of case 6.

**Figure 43:**
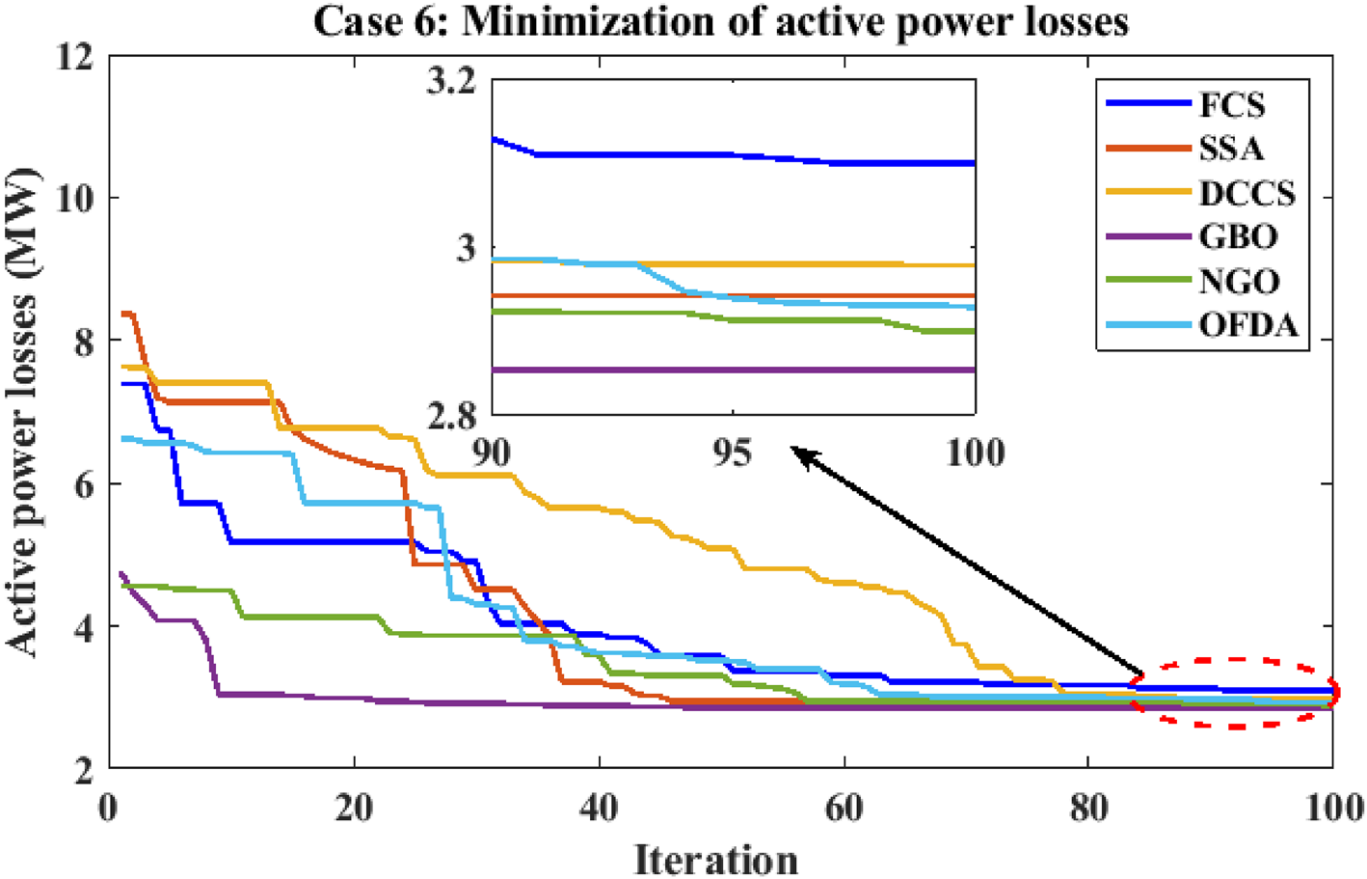
Variation of active power losses of case 6.

**Figure 44:**
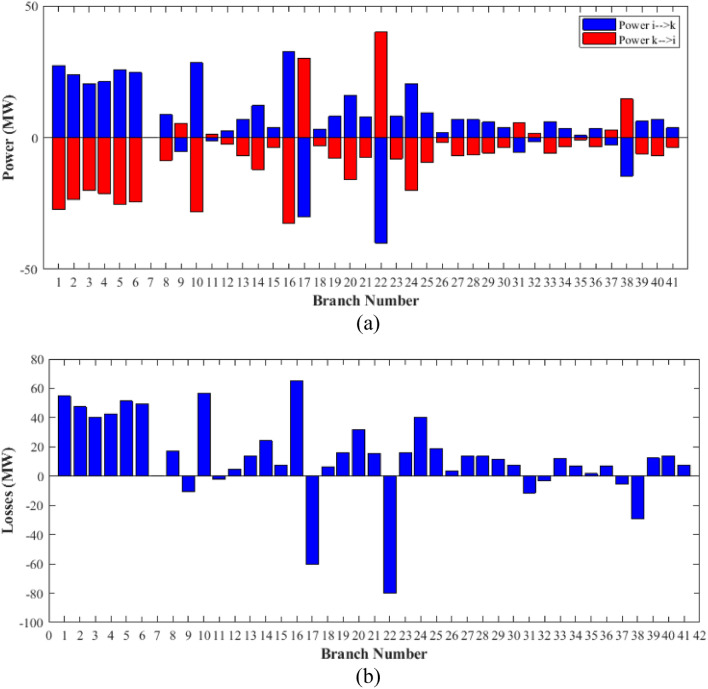
Active power flow and losses in the branches of the system for case 6.

**Figure 45:**
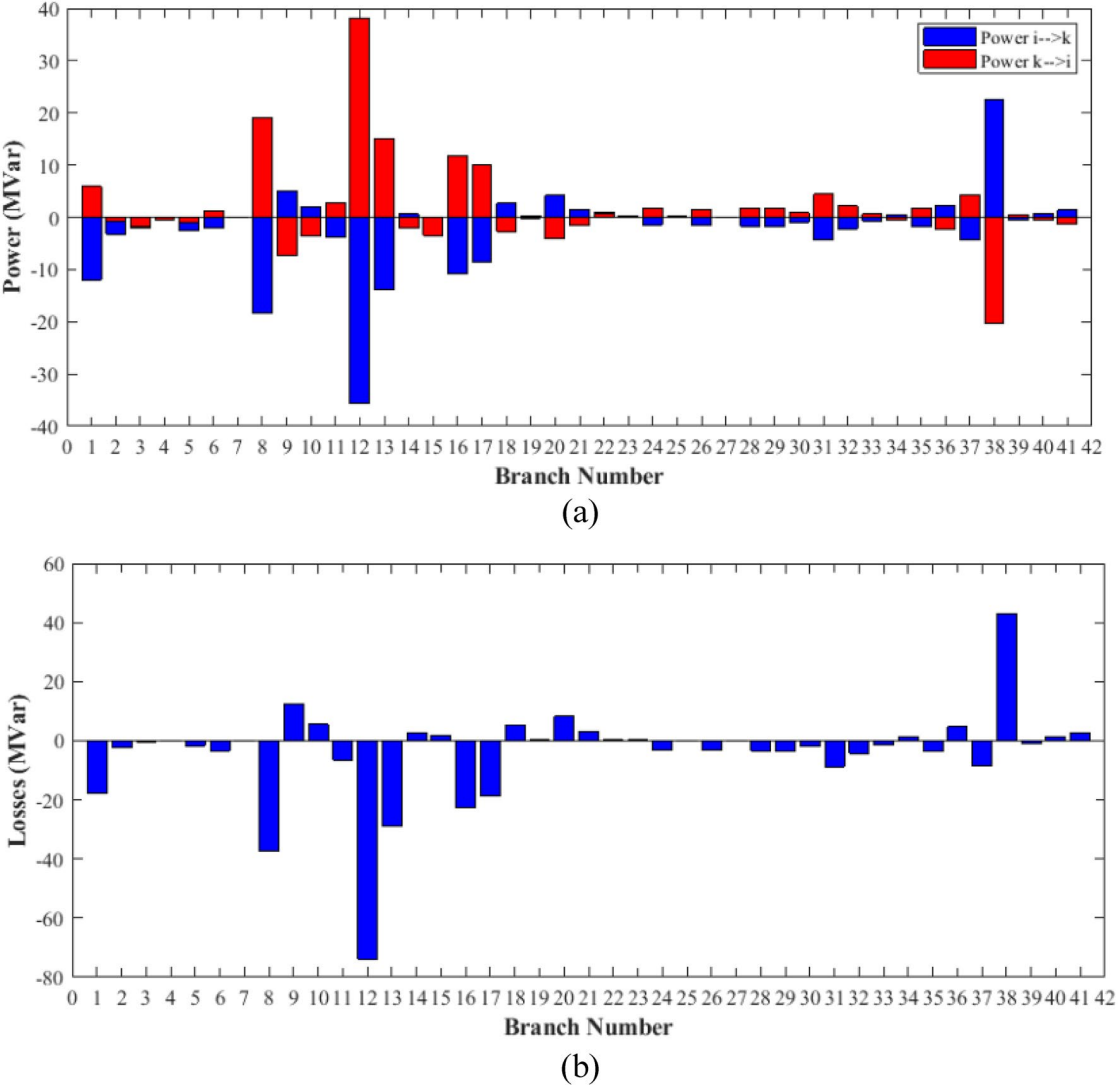
Reactive power flow and losses in the branches of the system for case 6.

**Figure 46:**
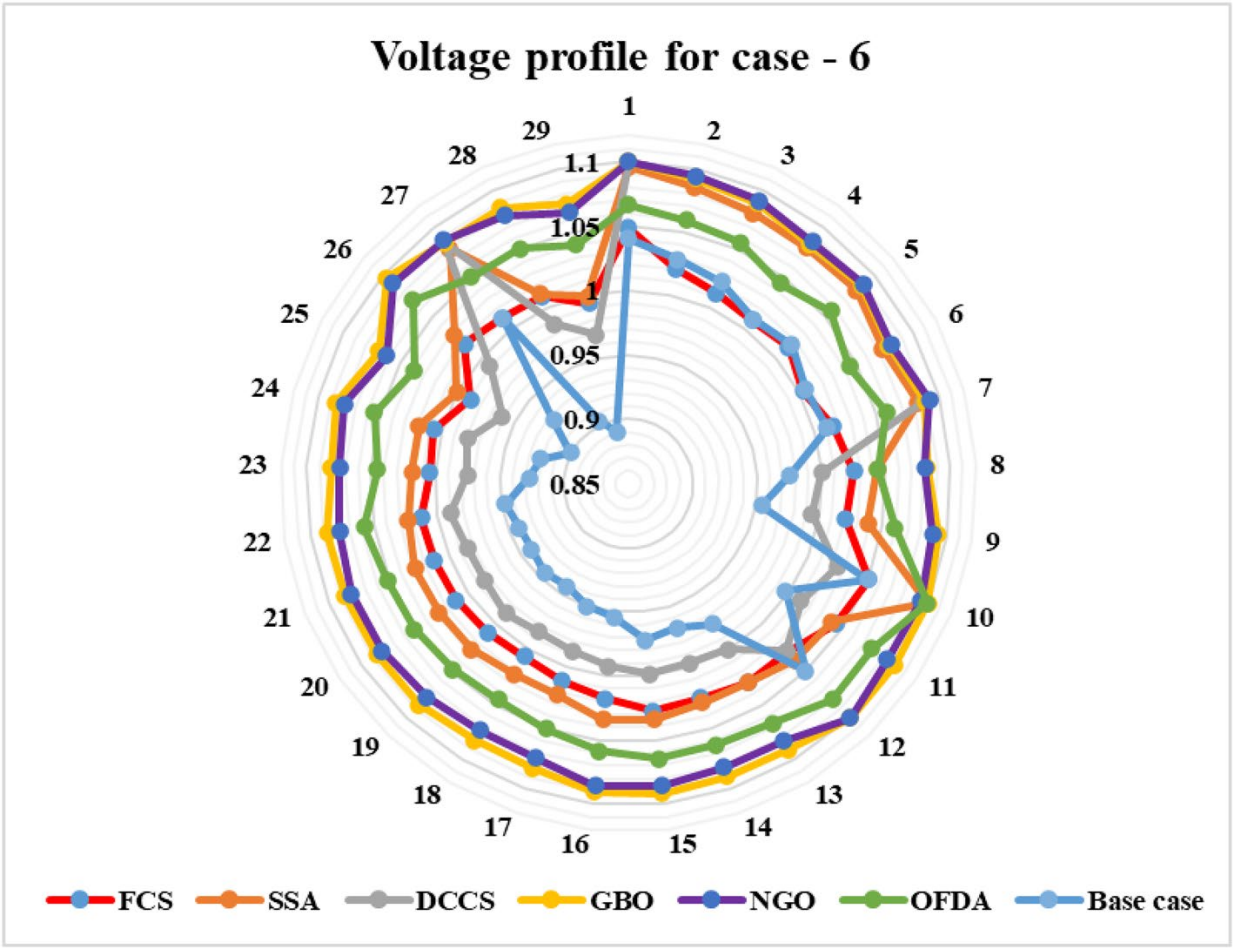
Voltage profile improvement for case 6.

The proposed algorithms have been implemented for 30 individual runs to address the optimization problem of OPF incorporating the objective function (minimization of active transmission power losses) of case 6. The obtained results of the best value of the voltage deviation in each run are recorded and presented in the graph shown in Fig. 41. Statistical study has been conducted and the results are listed in Table 17. A boxplot based on the 30 values of the total voltage deviation is sketched in Fig. 42. Also, in this case, the GBO optimization technique provided the best performance compared with the others. The minimum value of the fuel cost obtained from GBO is 2.5819 MW compared to 3.0994 MW for FCS, 2.9408 MW for SSA, 2.9773 MW for DCCS, 2.8983 MW for NGO, and 2.9273 MW for OFDA. The variation of the active power losses is provided in Fig. 43. The results of the optimization process for case 6 compared to the base case are provided in Table 18. The active power flow in the 41 branches is presented in Fig. 44a and the power losses in each branch is sketched in Fig. 44b. Similarly, the reactive power flow is presented in Fig. 45a and the reactive power losses in each branch is sketched in Fig. 45b. The impact of the optimization process on the voltage profile of the PQ buses of the system is presented in Fig. 46. Finally, the active and reactive power balance based on the results of the six proposed algorithms is provided in Table 19.

**Table 17:** Statistical results for case 6.

	Min.	Max.	Mean	SD	RMSE
FCS	3.0994	7.2122	4.2342	85.6207	1.4129
SSA	2.9408	4.0406	3.4756	29.2222	0.6071
DCCS	2.9773	8.1817	3.6463	91.8199	1.1237
GBO	2.8519	2.9768	2.8763	2.7239	0.0362
NGO	2.8983	3.5316	3.1305	19.4718	0.3009
OFDA	2.9273	3.6246	3.1513	16.4145	0.2761

**Table 18:** Optimization results for case 6.

Variable	Base	Min	Max	FCS	SSA	DCCS	GBO	NGO	OFDA
P1	99.2230	50	200	77.1859	69.6960	52.5605	51.2549	54.5824	51.5164
P2	80	20	80	76.6820	80.0000	78.9951	79.9999	76.9888	80.0000
P5	50	15	50	50.0000	50.0000	50.0000	50.0000	49.7706	50.0000
P8	20	10	35	30.3895	35.0000	34.9999	35.0000	34.9985	35.0000
P11	20	10	30	20.7948	30.0000	29.9920	30.0000	30.0000	29.9989
P13	20	12	40	32.7747	22.1384	39.9907	40.0000	39.9789	40.0000
V1	1.05	0.95	1.1	1.0530	1.1000	1.0998	1.1000	1.1000	1.0681
V2	1.04	0.95	1.1	1.0483	1.0959	1.1000	1.0999	1.1000	1.0664
V5	1.01	0.95	1.1	1.0091	1.0793	1.0825	1.0829	1.0856	1.0449
V8	1.01	0.95	1.1	1.0151	1.0833	1.0879	1.0889	1.0927	1.0579
V11	1.05	0.95	1.1	1.0510	1.0999	1.0242	1.1000	1.0944	1.0999
V13	1.05	0.95	1.1	1.0292	1.0388	1.0283	1.1000	1.1000	1.0803
T11	1.078	0.9	1.1	1.0028	1.0826	1.0988	1.0401	1.0351	1.1000
T12	1.069	0.9	1.1	0.9320	0.9725	1.0651	0.9191	0.9160	0.9000
T15	1.032	0.9	1.1	0.9050	1.0103	1.0874	0.9849	1.0013	0.9638
T36	1.068	0.9	1.1	0.9827	1.0309	1.0810	0.9733	0.9799	0.9514
QC10	0	0	5	0.0000	0.2846	0.0000	4.9993	4.0998	5.0000
QC12	0	0	5	0.5176	0.2163	4.9765	4.9984	2.4907	5.0000
QC15	0	0	5	3.5693	4.1380	3.8178	4.2140	4.8574	5.0000
QC17	0	0	5	0.9585	3.7279	5.0000	4.9998	5.0000	4.9999
QC20	0	0	5	0.0000	3.5651	4.9750	4.9693	2.7009	0.0001
QC21	0	0	5	3.7460	1.5463	4.9990	5.0000	4.9992	4.9994
QC23	0	0	5	0.5268	1.9761	5.0000	3.9377	2.0350	4.9658
QC24	0	0	5	3.4779	4.4049	0.0005	4.9984	4.9992	0.0065
QC27	0	0	5	4.2908	1.4402	4.9897	2.8943	2.7336	0.0000
Fuel cost ($/h)	901.9516	–	–	967.633	967.279	967.359	992.655	966.671	966.860
Active power losses (MW)	**5.8219**	–	–	**3.0994**	**2.9408**	**2.9773**	**2.8519**	**2.8983**	**2.9273**
Reactive power losses (MVar)	− 4.6066	–	–	− 22.9631	− 21.262	− 20.276	− 18.820	− 20.857	− 19.524
Voltage deviation	1.1496	–	–	0.8685	1.4848	1.4810	2.0447	1.7841	1.8976
Lmax	0.17233	–	–	0.1280	0.1224	0.1244	0.1155	0.1183	0.1169

Significant values are in bold.

**Table 19:** Active and reactive power balance for case 6.

Case-6	Active power balance			Reactive power balance				
method	load (MW)	Generation (MW)	Loss (MW)	Load (MVar)	Generation (MVar)	compensation (MVar)	charging (MVar)	Loss (MVar)
FCS	283.4000	287.8269	4.4269	126.2000	97.0164	17.0869	3.9466	− 8.1501
SSA	283.4000	286.8344	3.4353	126.2000	86.5572	21.2994	− 1.4657	− 19.8092
DCCS	283.4000	286.5382	3.1396	126.2000	69.7614	33.7584	0.8922	− 21.7880
GBO	283.4000	286.2547	2.8562	126.2000	62.6178	41.0112	3.7413	− 18.8297
NGO	283.4000	286.3193	2.9209	126.2000	69.8791	33.9157	2.6327	− 19.7726
OFDA	283.4000	286.5152	3.1155	126.2000	82.3822	29.9717	− 0.4359	− 14.2821
Base case	283.4000	289.2225	5.8225	126.2000	121.5936	0.0000	0.0000	− 4.6063

### Case-7

**Figure 47:**
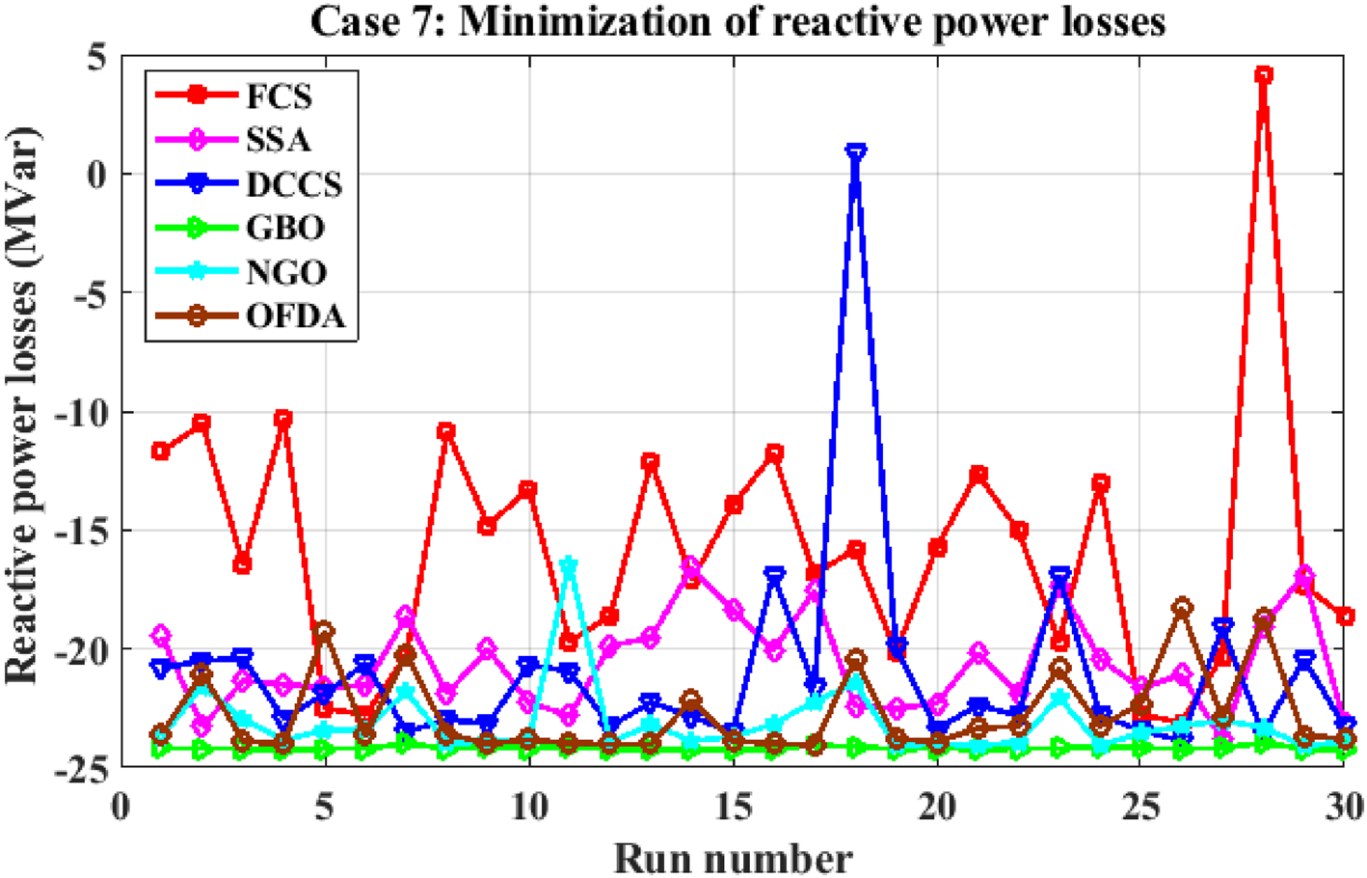
Variation of the objective function over the 30 runs for case 7.

**Figure 48:**
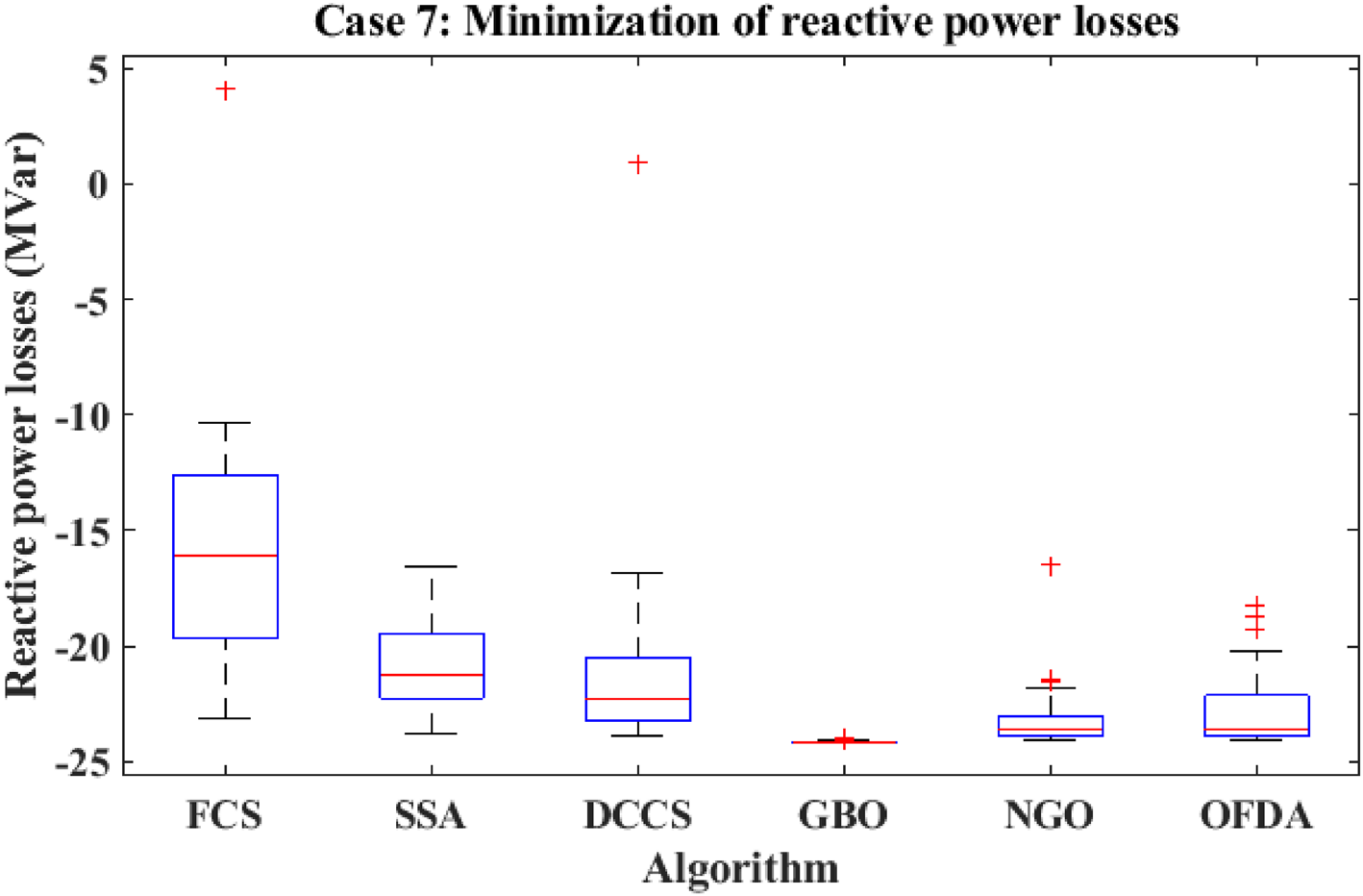
Boxplot for the results of the objective function of case 7.

**Figure 49:**
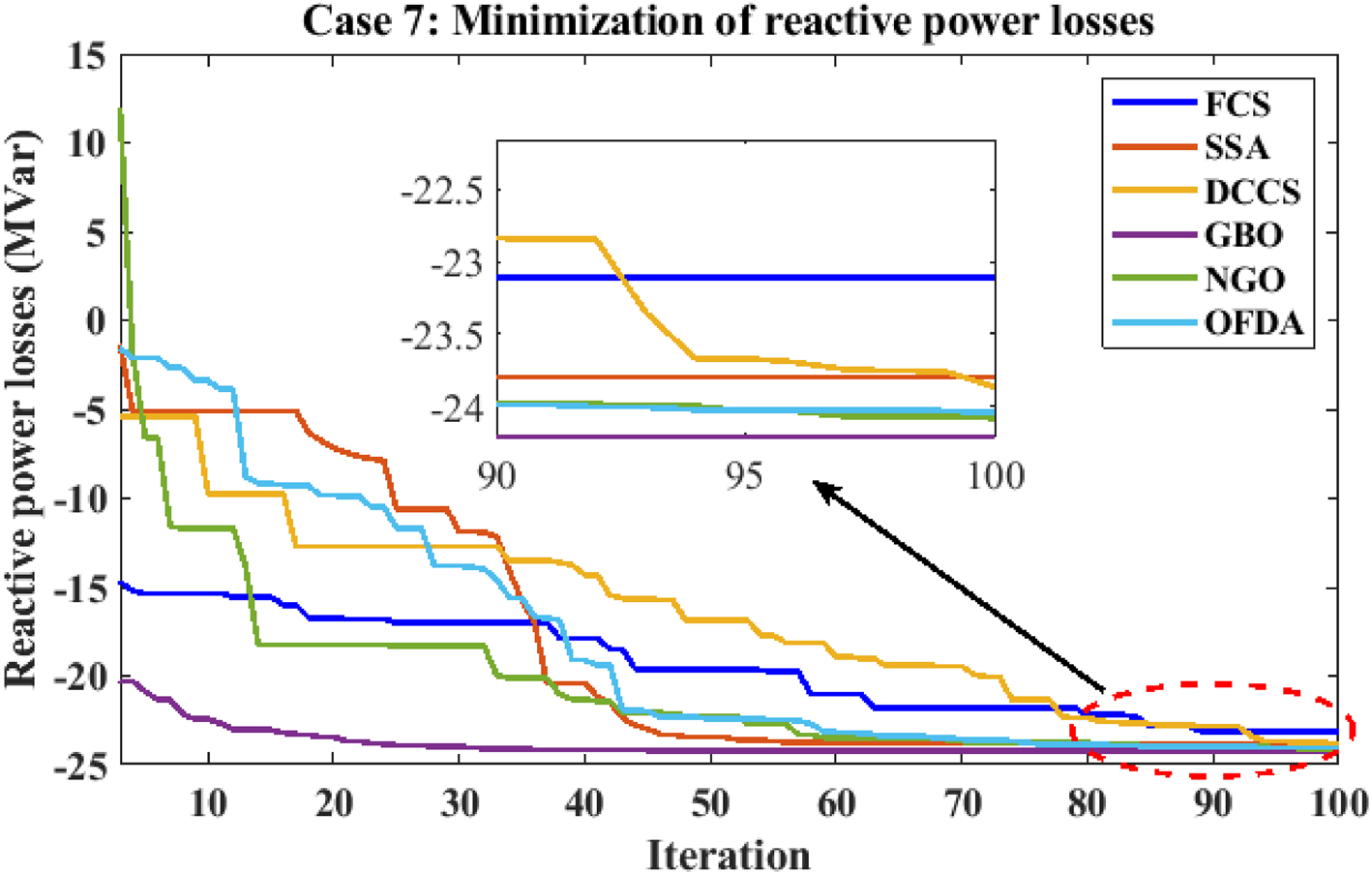
Variation of reactive power losses of case 7.

**Figure 50:**
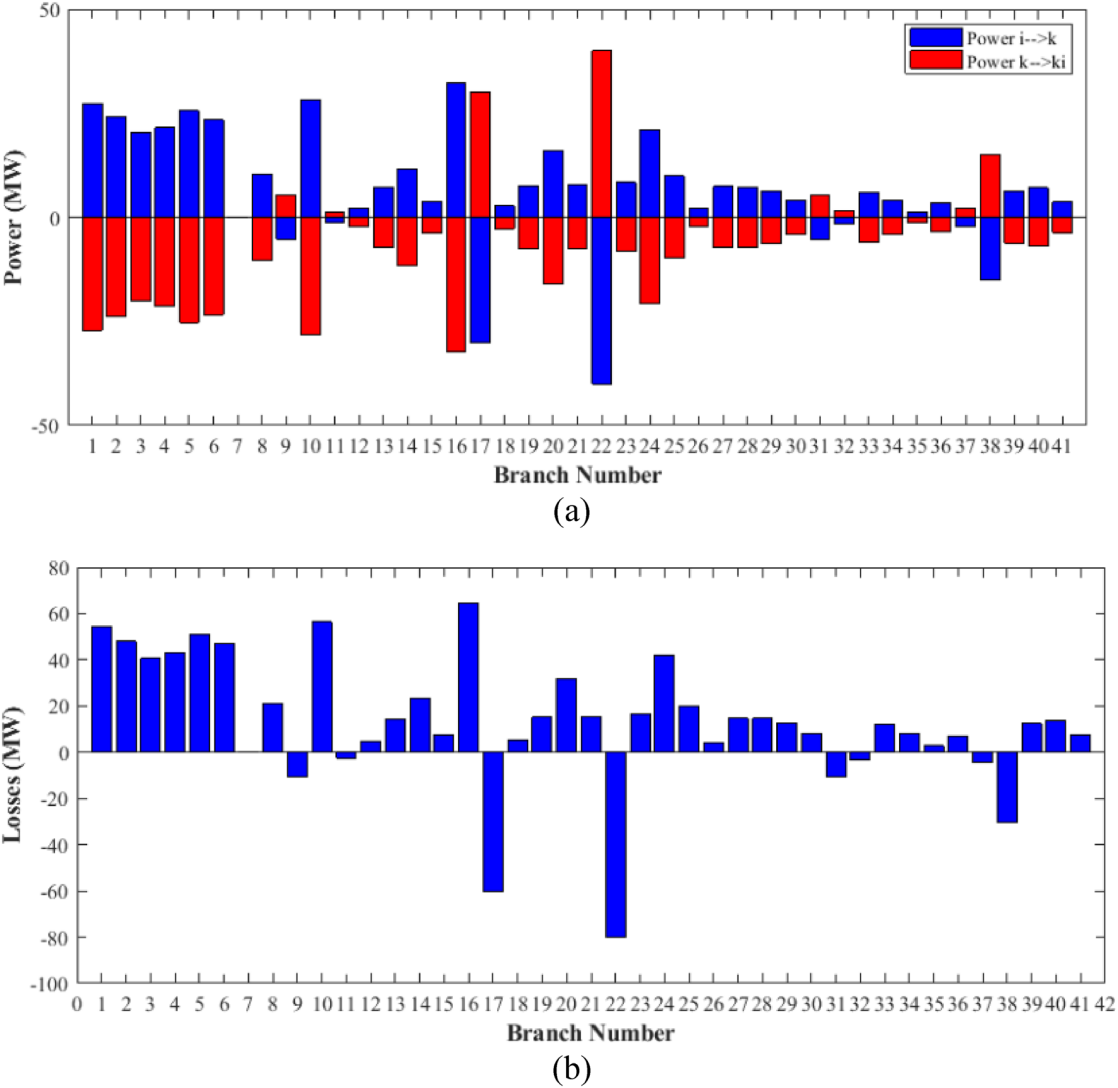
Active power flow and losses in the branches of the system for case 7.

**Figure 51:**
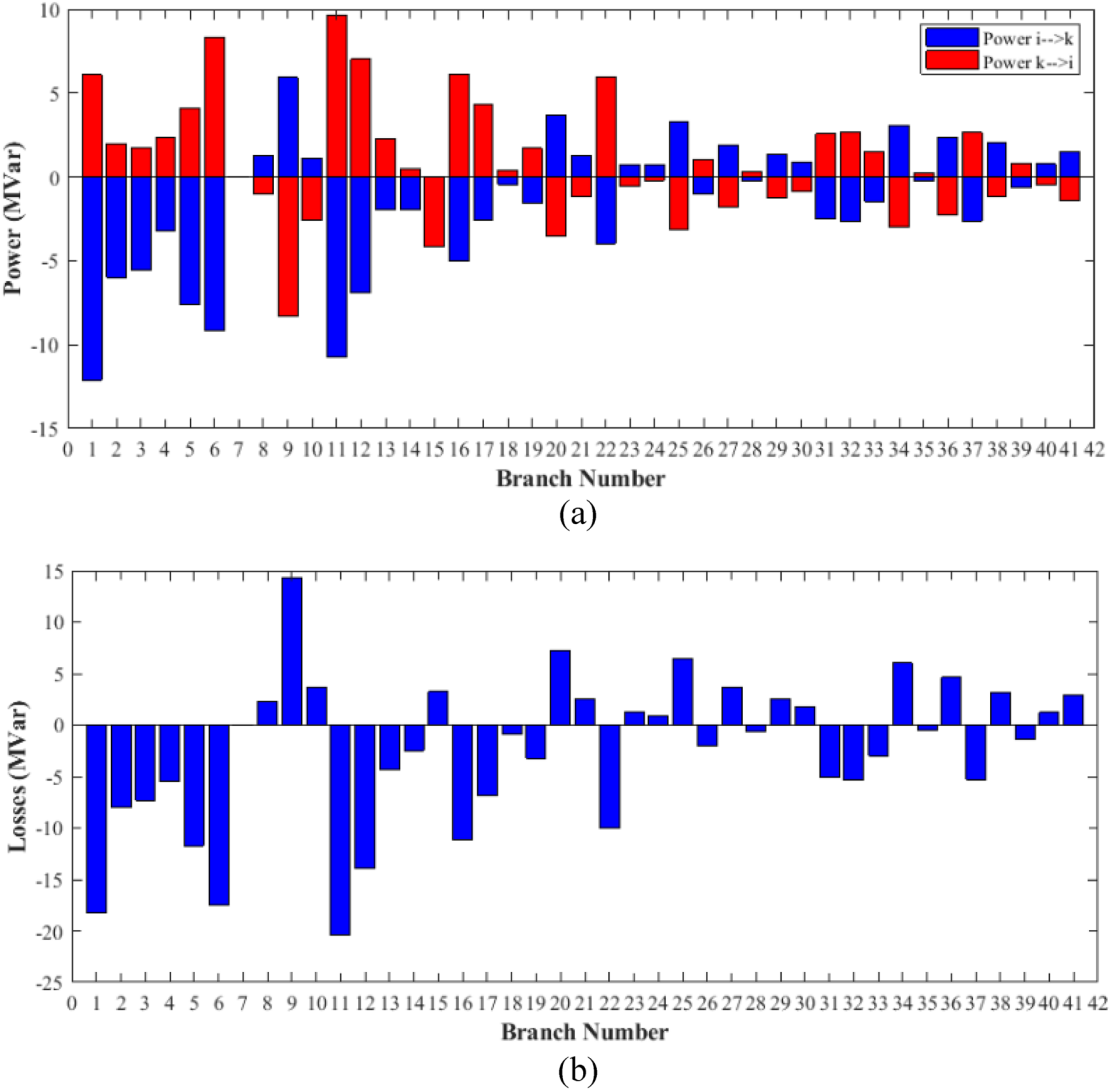
Reactive power flow and losses in the branches of the system for case 7.

**Figure 52:**
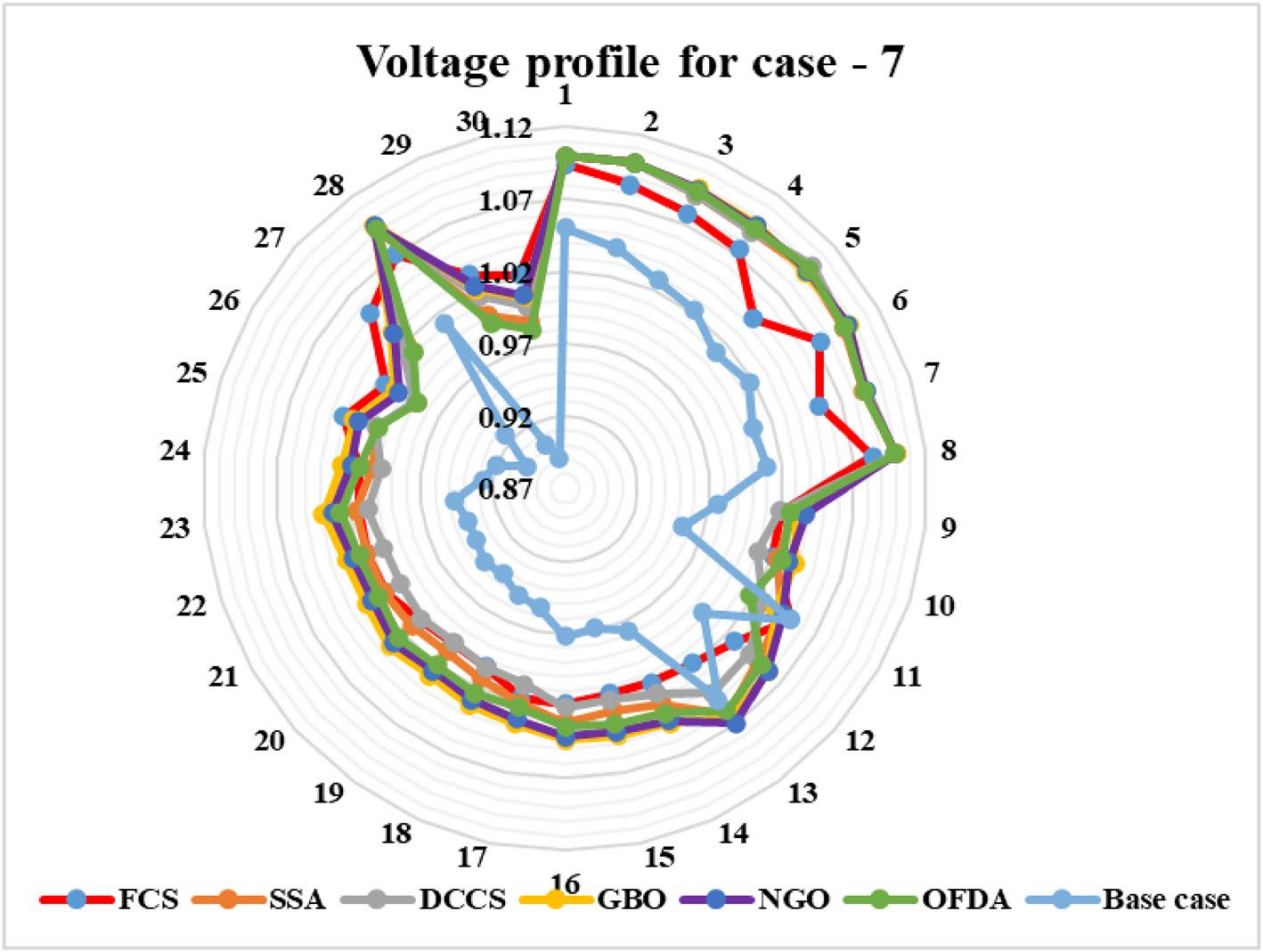
Voltage profile improvement for case 7.

The proposed algorithms have been implemented for 30 individual runs to address the optimization problem of OPF incorporating the objective function (minimization of reactive transmission power losses) of case 7. The obtained results of the best voltage deviation value in each run are recorded and presented in the graph shown in Fig. 47. Statistical study has been conducted and the results are listed in Table 20. A boxplot based on the 30 values of the total voltage deviation is sketched in Fig. 48. Also, in this case, the GBO optimization technique provided the best performance compared with the others. The minimum value of the fuel cost obtained from GBO is − 24.2129 MVar compared to − 23.1073 MVar for FCS, − 23.7994 MVar for SSA, − 23.8660 MVar for DCCS, − 24.0835 MVar for NGO, and − 24.0475 MVar for OFDA. The variation of the reactive power losses is provided in Fig. 49. The results of the optimization process for case 7 compared to the base case are provided in Table 21. The active power flow in the 41 branches is presented in Fig. 50a, and the power losses in each branch is sketched in Fig. 50b. Similarly, the reactive power flow is presented in Fig. 51a, and the reactive power losses in each branch is sketched in Fig. 51b. The impact of the optimization process on the voltage profile of the system's PQ buses is presented in Fig. 52. Finally, the active and reactive power balance based on the results of the six proposed algorithms is provided in Table 22.

**Table 20:** Statistical results for case 7.

	Min.	Max.	Mean	SD	RMSE
FCS	− 23.1073	4.1661	− 15.7889	545.5059	9.0733
SSA	− 23.7994	− 16.5901	− 20.6312	197.9806	3.7183
DCCS	− 23.8660	0.9160	− 20.9894	453.2368	5.3040
GBO	− 24.2129	− 23.9610	− 24.1669	6.7000	0.0803
NGO	− 24.0835	− 16.4180	− 23.1176	148.7195	1.7524
OFDA	− 24.0475	− 18.2653	− 22.7147	175.7975	2.1826

**Table 21:** Optimization results for case 7.

Variable	Base	Min	Max	FCS	SSA	DCCS	GBO	NGO	OFDA
P1	99.2230	50	200	83.5498	57.1191	53.0773	51.4497	52.3596	53.3713
P2	80	20	80	57.2983	79.9980	79.8466	80.0000	79.9924	78.5936
P5	50	15	50	48.7667	50.0000	49.9964	50.0000	49.9739	49.9934
P8	20	10	35	31.5137	34.3983	35.0000	35.0000	35.0000	34.9770
P11	20	10	30	26.2501	29.8079	29.2989	30.0000	29.6691	29.6437
P13	20	12	40	40.0000	35.4333	39.5211	40.0000	39.5282	39.9991
V1	1.05	0.95	1.1	1.0942	1.1000	1.1000	1.1000	1.1000	1.1000
V2	1.04	0.95	1.1	1.0849	1.1000	1.1000	1.1000	1.1000	1.1000
V5	1.01	0.95	1.1	1.0452	1.0956	1.1000	1.0930	1.0953	1.0967
V8	1.01	0.95	1.1	1.0831	1.1000	1.0999	1.1000	1.0992	1.0990
V11	1.05	0.95	1.1	1.0501	1.0427	1.0276	1.0378	1.0436	1.0174
V13	1.05	0.95	1.1	1.0190	1.0621	1.0440	1.0651	1.0703	1.0591
T11	1.078	0.9	1.1	1.0857	1.0645	1.0617	1.0792	1.0510	1.0569
T12	1.069	0.9	1.1	0.9792	1.0141	1.0995	1.0239	1.0778	1.0239
T15	1.032	0.9	1.1	1.0183	1.0105	1.0053	1.0267	1.0196	1.0250
T36	1.068	0.9	1.1	0.9772	1.0469	1.0475	1.0491	1.0525	1.0652
QC10	0	0	5	1.6986	2.6275	2.4835	5.0000	4.4342	4.7774
QC12	0	0	5	4.3218	0.9742	4.5990	4.9996	1.8969	5.0000
QC15	0	0	5	2.9399	0.0225	0.0653	5.0000	4.0170	4.9999
QC17	0	0	5	5.0000	1.1108	1.8754	5.0000	5.0000	0.0408
QC20	0	0	5	0.0000	0.2106	3.2585	4.9577	5.0000	4.9990
QC21	0	0	5	4.6207	3.5967	4.9498	5.0000	4.8784	4.9676
QC23	0	0	5	3.1802	1.7922	0.9588	5.0000	3.0479	3.6536
QC24	0	0	5	5.0000	1.2426	2.8103	5.0000	3.2761	4.9760
QC27	0	0	5	0.0622	1.3512	4.4686	3.1804	4.6388	1.5314
Fuel cost ($/h)	901.951	–	–	964.758	961.913	967.559	967.544	967.395	967.552
Active power losses (MW)	5.8219	–	–	3.2545	3.1701	3.1077	3.0523	3.0725	3.0558
Reactive power losses (MVar)	**− 4.6066**	–	–	**− 23.107**	**− 23.7994**	**− 23.8660**	**− 24.212**	**− 24.083**	**− 24.0475**
Voltage deviation	1.1496	–	–	1.0369	0.9704	0.9007	1.0688	1.0126	1.0514
Lmax	0.17233	–	–	0.1356	0.1301	0.1305	0.1273	0.1286	0.1312

Significant values are in bold.

**Table 22:** Active and reactive power balance for case 7.

Case-7	Active power losses			Reactive power losses				
Method	Load (MW)	Generation (MW)	Losses (MW)	Load (MVar)	Generation (MVar)	compensation (MVar)	charging (MVar)	Losses (MVar)
FCS	283.4000	287.3786	3.9791	126.2000	82.0827	26.8234	**− **1.3577	**− **18.6516
SSA	283.4000	286.7566	3.3582	126.2000	91.0397	12.9284	**− **0.9054	**− **23.1373
DCCS	283.4000	286.7404	3.3421	126.2000	78.2813	25.4691	**− **0.8013	**− **23.2509
GBO	283.4000	286.4497	3.0516	126.2000	59.1206	43.1377	**− **0.2706	**− **24.2124
NGO	283.4000	286.5233	3.1251	126.2000	66.0587	36.1893	0.0511	**− **23.9009
OFDA	283.4000	286.5782	3.1799	126.2000	67.8157	34.9457	26.6186	3.1799
Base case	283.4000	289.2225	5.8225	126.2000	121.5936	0.0000	0.0000	**− **4.6063

### CASE 8: IEEE 118-bus test system (minimization of the fuel generation cost)

**Figure 53:**
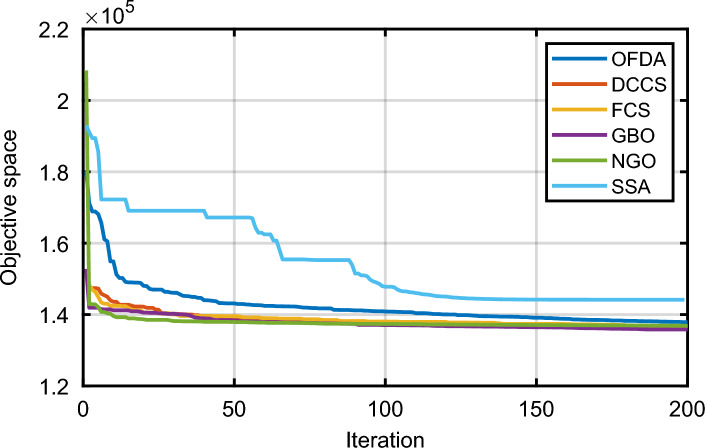
Variation of reactive power losses of case 8; IEEE 118-bus test system.

**Figure 54:**
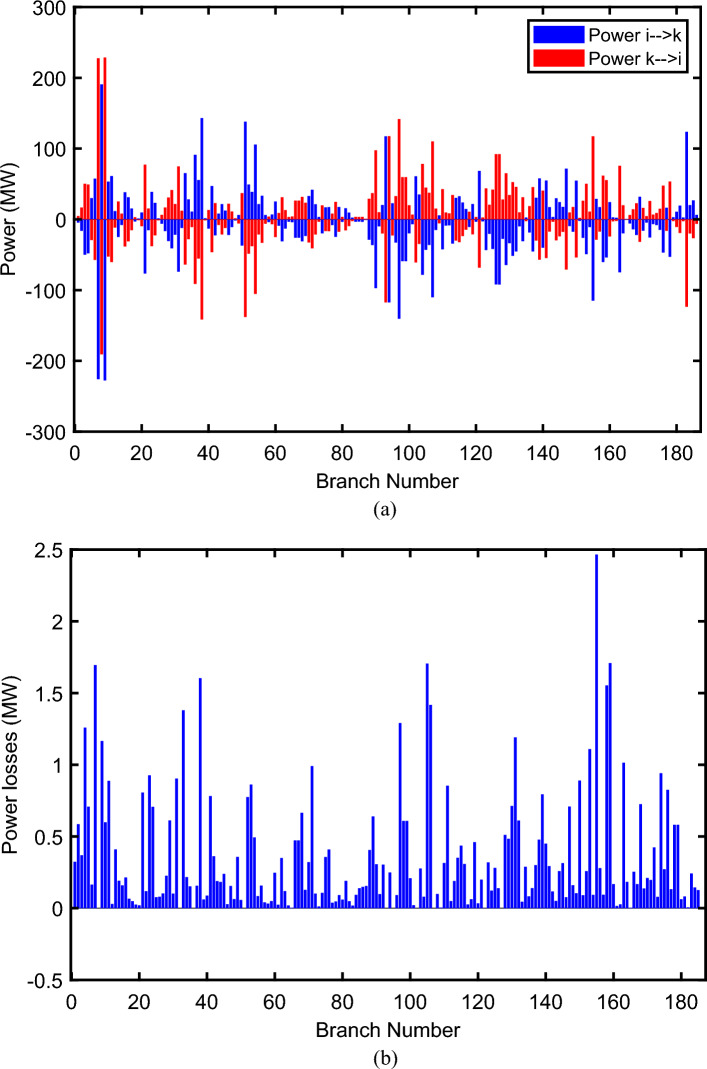
Active power flow and losses in the branches of the system for case 8; IEEE 118-bus test system.

**Figure 55:**
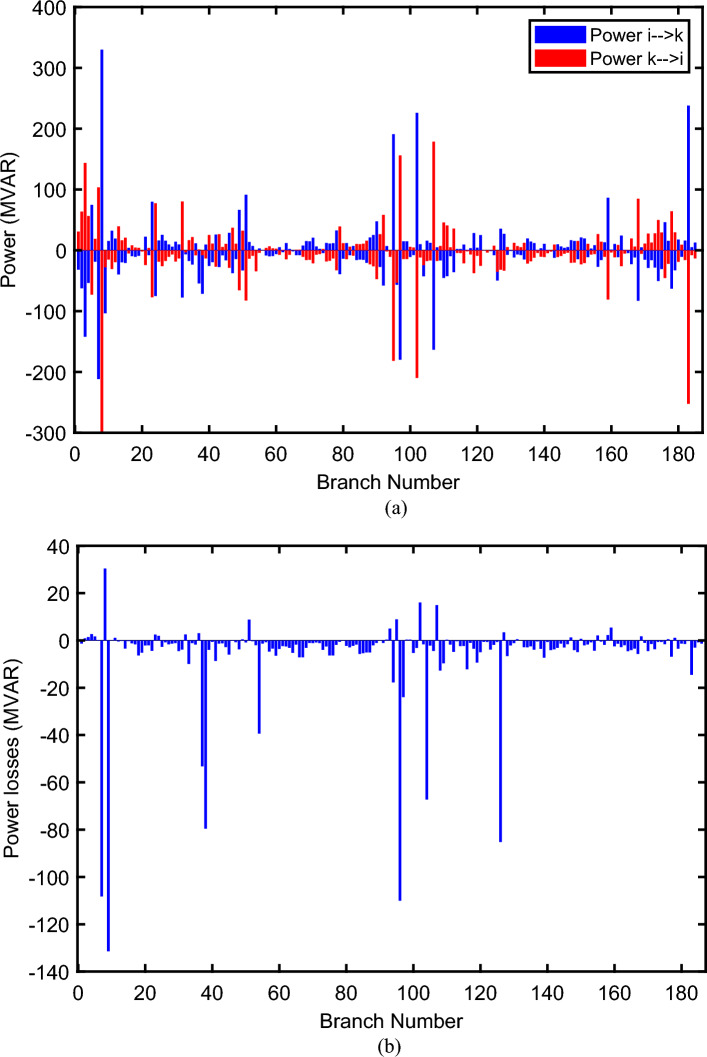
Reactive power flow and losses in the branches of the system for case 8; IEEE 118-bus test system.

**Figure 56:**
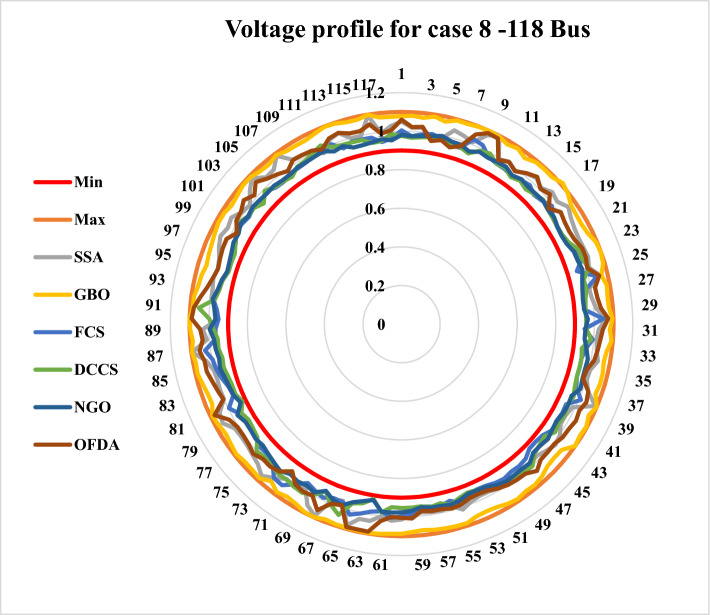
Voltage profile improvement for case 8; IEEE 118-bus test system.

This case study investigated into optimizing and minimizing fuel generation costs within an extensive electrical grid using computer simulation, specifically focusing on the IEEE 118-bus test system. This system featured 54 generators, 9 transformers with tap change capabilities, and 12 capacitors and 2 reactors for voltage and power flow regulation^48^. The primary aim was to pinpoint the most efficient control settings to reduce fuel generation costs. Simulations were carried out to assess the effectiveness of various optimization techniques (FCS, SSA, DCCS, GBO, NGO, and OFDA), with visual representations of the results presented in Figs. 53, 54, 55, 56. Figure 53, in particular, provides a comparative analysis of the convergence speed of each method towards an optimal solution. The GBO method yielded the lowest fuel cost, amounting to $135,803.19/h, outperforming other algorithms. The convergence curves of the proposed algorithms depicted in Fig. 53 underscored the robust convergence characteristics of GBO. The results conclusively demonstrated that GBO produced the most favorable outcomes, as detailed in Table 23. Table 23 outlines the optimization findings for CASE 8: IEEE 118-bus test system, including control variable values, fuel costs, voltage deviation, voltage stability index (Lmax), active power losses, and reactive power losses, comparing case 1 to the base case. The active power flow in the all branches is presented in Fig. 54a and the power losses in each branch is sketched in Fig. 54b. Similarly, the reactive power flow is presented in Fig. 55a and the reactive power losses in each branch is sketched in Fig. 55b. The impact of the optimization process on the voltage profile of the PQ buses of the system is presented in Fig. 56. Finally the active power balance based on the results of the six proposed algorithms is provided in Table 24.

**Table 23:** Optimization results for case 8, IEEE 118-bus test system.

	FCS	SSA	DCCS	GBO	NGO	OFDA
P 1	30	30.20849773	30.00103499	33.027247	32.216722	43.759878
P 2	31.21503114	42.2827314	30.00208142	30.001034	40.364545	45.459183
P 3	30	80.21268319	30.0009281	30.000019	31.373754	31.500305
P 4	36.67159491	48.48816863	30.0079558	30.071652	36.612611	32.396547
P 5	304.8405217	228.7494814	299.409708	374.89807	290.80247	287.89161
P 6	81.67098458	75.68424815	60.59247936	55.501459	71.373681	72.270407
P 7	33.32415014	30.53044299	30.29741249	30.013838	34.439467	37.327484
P 8	30.37412454	45.30894895	30.14819528	30.304168	42.93363	30.026987
P 9	30.48550124	42.6064532	30.00031533	30.031984	35.259902	30
P 10	35.6587977	31.54753325	30.1237268	30.78166	31.114536	31.724963
P 11	167.2056984	152.3481508	147.2579014	166.19237	136.12765	121.93216
P 12	194.3542829	131.0616259	231.6683308	124.20001	160.48676	180.34914
P 13	35.23433823	62.77492138	38.01895415	30.000921	31.949342	77.841795
P 14	32.1	32.1	32.12703691	32.100295	32.102384	32.1
P 15	39.36469936	41.60393373	30.18288256	30.047522	34.458918	31.5543
P 16	33.97308108	41.17627939	31.72285646	30.000588	35.233535	33.925888
P 17	33.4631064	47.20513801	33.0643674	30.000014	33.963708	30.69531
P 18	38.17599792	46.14346618	30.05424558	30.01383	33.716673	30
P 19	55.84398595	41.72399146	50.33892892	42.158953	42.214716	42.525091
P 20	35.7	42.9313123	36.06105302	35.700211	37.09738	36.659379
P 21	160.5258207	154.0083406	186.4265834	159.23599	148.21054	134.35324
P 22	48.91420234	63.32502801	44.67599963	44.463629	49.198303	55.482155
P 23	53.34014596	87.79918846	32.80779316	30.000041	51.658138	39.806978
P 24	31.80536535	90.82031852	30.4410279	30.007369	53.762847	39.596365
P 25	129.1539682	105.8067	83.45883876	85.602951	135.73404	115.40565
P 26	86.50598447	132.0609771	134.5349577	92.565003	115.54311	128.02031
P 27	35.34037184	40.48909397	39.18097115	30.000474	30.089449	33.141904
P 28	255.1494837	157.1452237	303.1138696	305.72755	283.90333	220.46355
P 29	281.4727861	152.5392763	286.5403959	303.07739	243.00761	310.98676
PGS	30	96.19247913	30.03186329	30.944449	39.318458	30
P 31	30	30.18275485	30.00817492	30.000004	34.682588	46.340053
P 33	38.13188651	40.45374246	33.22098317	30.000082	36.417912	49.881268
P 34	31.77696355	52.67549095	37.48445374	30.000063	34.142769	32.909006
P 35	30	63.54451513	30.00500258	30.003609	38.638176	43.615117
P 36	30	30.96161229	30.00015636	30.000478	34.158732	30.937087
P 37	328.5727147	241.6468347	376.3044614	363.10346	320.09243	318.44187
P 38	31.7593927	96.92977551	30.20288968	30.024439	35.530407	42.952776
P 39	31.40269091	31.20002244	31.2	31.20001	31.218104	31.2
P 40	377.7863392	225.0305002	362.0171001	409.27033	335.75561	401.81888
P 41	34.14409514	35.85736895	30.04101733	30.000121	36.305659	36.599979
P 42	30	53.92526093	30.05366965	30.004263	43.147468	39.726319
P 43	35.88097162	35.82413784	30.35215444	30.000086	30	39.524808
P 44	30	38.55668743	30	30.000029	37.081903	30.380717
P 45	166.7706869	198.315171	118.0050762	170.10331	149.15935	128.03783
P 46	42	85.06747738	44.82169299	42.000018	44.522139	42.242544
P 47	40.49506794	35.57025704	33.73079352	30.004233	38.420219	39.313405
P 48	30	71.3105953	35.88554433	30.00051	35.50375	30.406858
P 49	32.4450531	92.21462658	32.09986622	30.000021	36.990011	33.266239
P 50	30	32.61999859	30.49825091	30.000021	30.951291	31.755541
P 51	45.26384893	47.71135304	40.8000526	40.803233	42.914792	44.016222
P 52	31.83479461	51.76489995	37.44291248	35.309799	35.459012	30
P 53	35.72420901	57.0824873	30.00704161	30.004879	33.914246	31.332374
P 54	33.36611321	60.49993701	38.95198758	45.609769	30.315917	30
V1	1.004273662	0.952039204	0.975794484	1.09936	0.9810266	1.0597624
V4	0.997546979	1.040226741	0.981290231	1.0985454	0.9874708	0.9610048
V6	0.96167122	1.009897321	0.964525327	1.0992296	0.981436	0.95
V8	1.029326485	1.029212569	0.950064621	1.0991349	0.9617258	1.0557733
V10	0.978891193	1.097844802	0.99614006	1.09908	0.9717031	1.0847925
V12	0.966025365	1.014854273	0.971394944	1.0978626	0.9796776	1.0089754
V15	0.980753571	1.029628606	0.950415275	1.0968113	0.9681099	1.0379925
V18	0.991695861	0.985519616	0.964489512	1.0979516	0.9632388	0.9712686
V19	0.960122372	1.02048413	0.957456334	1.0951464	0.9666825	1.0090115
V24	0.994146792	0.984160482	0.995049078	1.0837314	0.9714193	1.0164807
V25	0.95	0.991791336	1.01406645	1.0975695	0.9802907	1.0062602
V26	1.043971497	1.024607471	0.972716671	1.0986804	0.9651179	1.0560223
V27	0.974119275	0.973271731	0.975888428	1.0986889	0.9554454	1.0215464
V31	0.95	1.011369516	0.95866085	1.0990607	0.9542608	1.0445987
V32	0.99443195	0.992183431	0.986685157	1.098482	0.9534102	1.0345862
V34	0.95	1.044260298	0.950119948	1.0991901	0.9835445	1.0170946
V36	0.95	1.026644271	0.950000343	1.0980449	0.9802388	0.983445
V40	0.953574625	1.012780652	0.969079239	1.0994563	0.9574019	1.0346484
V42	0.956526633	1.020518502	0.9737759	1.0994466	0.9544724	1.0211159
V46	0.95	1.063371603	1.001672182	1.0996385	0.9806164	1.0354635
V49	0.962254845	1.053176012	1.000299692	1.0990378	0.9799909	1.0000264
V54	0.97304698	0.99812777	0.950118788	1.0980401	0.9617614	0.9859521
V55	0.95	0.990569508	0.970970994	1.0991434	0.9704911	0.9904626
V56	0.965057322	0.995672283	0.950629078	1.0965015	0.96445	0.9808873
V59	0.988203476	1.029960845	0.95213289	1.0998386	0.9700843	1.0090019
V61	1.005040702	1.003669456	0.950001673	1.0984243	0.9782984	1.0010401
V62	0.976539364	1.023587419	0.981347853	1.0984549	0.978457	1.026559
V65	1.02299766	1.03276947	0.963139745	1.0992017	0.9573372	1.090444
V66	0.972939442	1.050619492	1.041624711	1.0947485	0.989326	0.9888073
V69	0.973117668	1.042936026	0.998984559	1.0986089	0.9837429	1.0021786
V70	0.95651271	1.033460726	1.014812136	1.0990947	0.9709839	1.0078006
V72	1.040300095	1.068612067	1.003193543	1.0989817	0.9686196	0.9506764
V73	1.036531729	1.070713782	1.006017478	1.0971826	0.9874331	0.9929408
V74	0.98569664	1.017407607	0.996563389	1.0983057	0.975293	1.0051893
V76	0.972488802	1.006542308	0.999037107	1.0790894	0.9554396	1.0215992
V77	0.955849206	1.003709475	0.950006388	1.0957765	0.9628916	1.0088057
V80	0.959221596	1.01015269	0.950047925	1.0959378	0.9739943	1.0225624
V85	0.989618105	1.035219573	0.950001336	1.0990217	0.9688851	1.0397841
V87	1.028654637	1.081412338	0.957653236	1.0981553	0.9726276	1.0570598
V89	0.965770796	1.011963379	0.976481995	1.0988748	0.9956617	1.0448624
V90	0.95	0.99137809	0.997303942	1.0996752	0.9655675	1.0896835
V91	0.961212524	0.992559966	1.055078324	1.0991078	0.9740328	1.0803192
V92	0.985182805	1.005166825	0.975813203	1.0998375	0.9879487	1.0542356
V99	0.981680435	1.054344126	0.980850001	1.0992224	0.967154	0.9755029
V100	0.97747106	0.997588723	0.993705772	1.0914954	0.9842761	1.0110403
V103	0.952777656	0.996410667	0.950042808	1.0959901	0.9766729	1.0143975
V104	0.987117168	1.000245076	0.989726512	1.0977185	0.9733511	1.055689
V105	0.962909301	1.033636471	0.968394768	1.0992992	0.9757867	1.0268212
V107	0.969848439	1.064954729	0.951971941	1.0995527	0.9678228	0.9888678
V110	0.986186726	1.08736995	0.998111905	1.0984485	0.9778641	1.0125434
V111	0.980070391	1.064859038	1.01648283	1.0994849	0.982467	0.995129
V112	1.000746003	1.07240674	0.985608411	1.0989254	0.9609044	1.0344558
V113	0.959363333	1.052679804	0.978261171	1.0952988	0.9717837	1.0434104
V116	0.978433982	1.025184756	0.951419778	1.075234	0.9544486	1.0479702
QC5	9.885821153	6.399368464	23.00207864	14.604473	7.6282153	24.811947
QC34	11.59824541	10.0996899	1.849497712	1.7742424	4.4760745	4.4194683
QC37	18.75317498	16.46138171	0.119915267	2.1236342	4.3701152	20.355924
QC44	5.863262582	14.65017076	0.70736558	9.3271734	9.0190765	7.2297207
QC45	5.479749647	17.22162196	1.780118836	1.7425522	3.3299186	22.657026
QC46	9.566035545	9.392945073	0.000834475	24.344103	12.578069	10.951006
QC48	1.593153868	16.62201694	0.72977663	0.0039744	10.410663	9.2206522
QC74	4.10608671	14.20160382	11.78005022	1.2716708	3.1570311	24.88065
QC79	1.854602284	23.76256076	0.616941778	0.0014579	10.207583	19.133327
QC82	15.17393593	1.260550559	17.47873479	0.0840153	3.4901652	2.8082702
QC83	0	2.541362414	0.910266205	10.270537	10.790201	20.711735
QC105	3.679472431	13.25169489	3.485593589	10.969319	3.9959176	24.369906
QC107	0	21.47275736	0.002354841	1.7857562	4.7632922	1.7136449
QC110	21.01033192	3.22E+00	6.43E+00	1.31E−06	1.7609552	5.7807158
Τ8-5	1.1	0.911580813	0.935927918	0.9955935	0.9285062	1.0503809
Τ26-25	0.923204432	1.066305571	0.905069126	1.0923423	0.9211049	0.9870213
τ30-Ι7	1.1	1.007119497	0.954515706	1.0993593	0.9789039	1.0662187
Τ38-37	0.997183573	0.952939129	1.004841544	1.0990938	0.9478267	0.957428
τ63-59	0.962207629	0.94660161	0.912407444	1.0984555	0.9299495	1.0863997
τ64-6Ι	1.006381785	0.929630414	1.03278757	1.048761	0.9590085	1.0756127
Τ6546	0.932124004	0.918186755	0.933421893	1.0683724	0.9011645	1.0997938
Τ68-69	0.977262648	1.055943548	0.95800589	1.0852384	0.9124949	1.0257545
Τ8Ι-80	1.035623187	1.015429732	0.9	1.0007588	0.9089533	1.0732612
Objective function	136,786.9071	144,150.6933	136,597.7483	135,803.19	136,864.92	137,782
Ploss	75.99303584	64.86057552	78.57205501	63.091982	57.997187	81.723654
VD	2.373459213	1.466437201	2.524643735	6.0049234	2.3912459	1.4362691
Lmax	0.077852815	0.061456179	0.07279492	0.0571464	0.073111	0.0663668

**Table 24:** Active power balance for case 8, IEEE 118-bus test system.

Case-7	Active power losses		
Method	Load (MW)	Generation (MW)	Losses (MW)
FCS	4317.993	4242	75.993035
SSA	4306.860	4242	64.860575
DCCS	4320.572	4242	78.572055
GBO	4305.091	4242	63.091982
NGO	4299.997	4242	57.997187
OFDA	4323.723	4242	81.723653
Base case	4375.357	4242	133.35777

## Conclusion

In this work, Fast Cuckoo Search (FCS), Salp Swarm Algorithm (SSA), Dynamic control Cuckoo search (DCCS), Gradient-Based Optimizer (GBO), Northern Goshawk Optimization (NGO), Opposition Flow Direction Algorithm (OFDA) in order to address the OPF issue are suggested. For the purpose of evaluating the performance of the suggested strategies on the IEEE 30 bus test system, seven OPF formulations, various objectives, and limitations are taken into account. The outcomes of the scenarios examined show that (i) the suggested GBO technique is highly efficient and robust when compared to other widely recognized methods, and (ii) the suggested approach can be applied to a wide range of cases with multifaceted objective functions, security constraints, restricted zones, and various test systems. Moreover, a case study to explore the optimization and minimization of fuel generation costs in a large-scale electrical grid using computer simulation, with a particular emphasis on the IEEE 118-bus test system has been presented. The results of scuch case of IEEE 118-bus test system prove also the superiority of the GBO algorithm. Given the promise and excellent qualities of the suggested GBO, it is advised that a multi-objective algorithm based on GBO be created and used to address multi-objective OPF issues.

## Data Availability

The data that support the findings of this study are available from the corresponding author upon reasonable request.
